# Addressing Clinical Limitations of Glutaminase Inhibitors: Novel Strategies for Osimertinib‐Resistant Lung Cancer by Exploiting Glutamine Metabolic Dependency

**DOI:** 10.1002/advs.202411479

**Published:** 2024-12-16

**Authors:** Jiali Huang, Xiankang Zhang, Hui Zhang, Yu Li, Huidan Huang, Zhiyu Li, Zhixia Qiu, Hongxi Wu, Dechun Huang, Xi Xu, Jinlei Bian

**Affiliations:** ^1^ Jiangsu Key Laboratory of Drug Design and Optimization Department of Medicinal Chemistry China Pharmaceutical University Nanjing Jiangsu 210009 China; ^2^ Department of Biomedical Engineering School of Engineering China Pharmaceutical University Nanjing Jiangsu 210009 China; ^3^ Center of Drug Screening & Evaluation Wannan Medical College Wuhu Anhui 241000 China

**Keywords:** drug design, glutamine, NQO1, NSCLC, Osimertinib resistance

## Abstract

Overcoming acquired resistance to Osimertinib remains a critical challenge in treating NSCLC. This research indicates that Osimertinib‐resistant cells exhibit a strong dependence on glutamine metabolism. However, targeting GLS1 shows limited anticancer effects, probably because it cannot fully block the glutamine metabolic pathway. The investigation reveals that a more effective strategy involves simultaneously inhibiting both ASCT2 and GLS1. After confirming the efficacy of this dual‐targeting approach against Osimertinib‐resistant cells in preclinical models, the potential of utilizing a broad‐spectrum glutamine metabolism antagonist is further explored to achieve superior antitumor efficacy. DON, broad‐spectrum glutamine antagonist, presents toxicity issues. Herein, the high NQO1 expression in Osimertinib‐resistant NSCLC cells is leveraged to design an NQO1‐responsive DON prodrug, 10e (LBJ‐10e). This prodrug demonstrates superior safety compared to natural DON and greater antitumor activity against resistant tumors compared to the clinical phase II drug DRP104. These findings may address the clinical limitations of GLS1 allosteric inhibitors and underscore prodrug strategies in effectively treating Osimertinib‐resistant lung cancer, providing a foundation for future clinical trials.

## Graphical abstract

Glutamine metabolism in Osimertinib‐resistant NSCLC is complex and multifaceted; therefore, a comprehensive inhibition strategy targeting multiple nodes, such as ASCT2 and GLS1, or employing broad‐spectrum inhibition is essential to prevent rapid metabolic rewiring. The NQO1‐activated DON prodrug shows significant promise in this context, underscoring its potential to achieve a favorable therapeutic index.

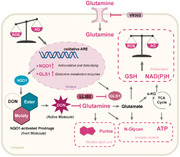

## 1 Introduction

The most recent research has uncovered that the mortality trends of cancer are predominantly shaped by lung cancer, which consistently accounts for significantly higher annual deaths compared to the combined mortality of colorectal, breast, and prostate cancer.^[^ ^1^ ^]^ Non‐small cell lung cancer (NSCLC), the most prevailing histological subtype of lung cancer, comprises ≈85% of all patients.^[^ ^2^ ^]^ Typically, surgical resection is the standard therapeutic approach for early‐stage NSCLC, while patients with advanced NSCLC predominantly receive pharmacological therapy. Epidermal growth factor receptor tyrosine kinase inhibitor (EGFR‐TKI) represents the primary choice for patients with advanced NSCLC harboring EGFR mutations. Osimertinib, an efficient and irreversible third‐generation EGFR‐TKI, is prescribed as a standard first‐line treatment for patients with advanced or metastatic NSCLC bearing EGFR mutations due to its remarkable antitumor efficacy and manageable safety profile.^[^ ^3^, ^4^, ^5^, ^6^ ^]^ However, similar to other EGFR‐TKIs, Osimertinib inevitably leads to acquired resistance, constraining its therapeutic efficacy in patients with EGFR‐mutated NSCLC.^[^ ^2^, ^7^, ^8^, ^9^ ^]^ The mechanisms underlying Osimertinib resistance are complex and varied; thus, precision medicine guidelines necessitate tailoring treatment regimens based on dynamic changes in resistance patterns, resulting in increased complexity and limitations within precision medicine strategies that ultimately contribute to further disease progression. Therefore, there is an urgent need for broad‐spectrum therapeutic alternatives to address acquired resistance in malignant NSCLC.

In our investigation, we demonstrated that Osimertinib‐resistant NSCLC cells exhibit significantly elevated energy and anabolic demands, along with exceptionally high levels of oxidation and reduction. Further analyses revealed a profound dependence on glutamine metabolism. Glutamine serves multiple roles in cellular metabolism, and glutaminase (GLS1)‐mediated hydrolysis not only provides energy and reducing equivalents but also supplies metabolic intermediates as biosynthetic precursors for the synthesis of non‐essential amino acids, nucleotides, and fatty acids.^[^ ^10^ ^]^ Consequently, we contemplated developing therapeutics targeting glutamine metabolism to refine the treatment strategy for Osimertinib‐resistant NSCLC.

Recent research has focused on allosteric inhibitors of GLS1 in tumor glutamine metabolism; however, progress has been limited and clinical trials have been halted.^[^ ^11^, ^12^ ^]^ Our studies demonstrated that both the novel macrocyclic GLS1 inhibitor LL202^[^ ^13^ ^]^ developed by our group and the clinical drug CB839^[^ ^14^ ^]^ did not elicit satisfactory anti‐tumor activity in vivo as monotherapies. Nevertheless, the present study exposed that concurrent blockade of the glutamine transporter alanine‐serine‐cysteine type 2 (ASCT2, encoded by SLC1A5) and GLS1 could generate significant anti‐tumor activity with a manageable safety profile. This dual‐target approach may elucidate the limited success of selective GLS1 inhibitors and suggests that a broad‐spectrum strategy could be more effective. 6‐diazo‐5‐oxo‐l‐norleucine (DON) is the most recognized broad‐spectrum glutamine antagonist; however, it is associated with severe toxicities.^[^ ^15^, ^16^ ^]^ To address this issue, we leveraged the high redox level of Osimertinib‐resistant cells to design antioxidant factor NAD(P)H: quinone oxidoreductase 1 (NQO1)‐responsive DON prodrug molecules, aiming to reduce toxic side effects while enhancing therapeutic potency. Our phenotype‐based prodrug molecule proved more effective than esterase‐ and aminopeptidase‐based prodrug molecule DRP104 in drug‐resistant cell models.^[^ ^17^, ^18^, ^19^, ^20^ ^]^ In addition to modulating tumor energy metabolism, NQO1‐responsive prodrug could further augment the anti‐tumor effect by enhancing the immune microenvironment and activating protective immune response.

This study offers a novel therapeutic combination for enhancing the in vivo efficacy of GLS1 allosteric inhibitors targeting glutamine metabolism. Specifically, the combination of the novel macrocyclic GLS1 inhibitor LL202 and ASCT2 inhibitor V9302 is employed to disrupt both glutamine uptake and metabolism. The identified NQO1‐responsive DON prodrugs demonstrate highly promising anti‐tumor activities both in in vitro and in vivo models of resistance. These findings present a potential therapeutic alternative for addressing the current challenge posed by Osimertinib‐resistant NSCLC in clinical settings.

## 2 Results

### 2.1 Osimertinib‐Resistant Lung Cancer Cells are Addicted to Glutamine Metabolism

**Figure 1:**
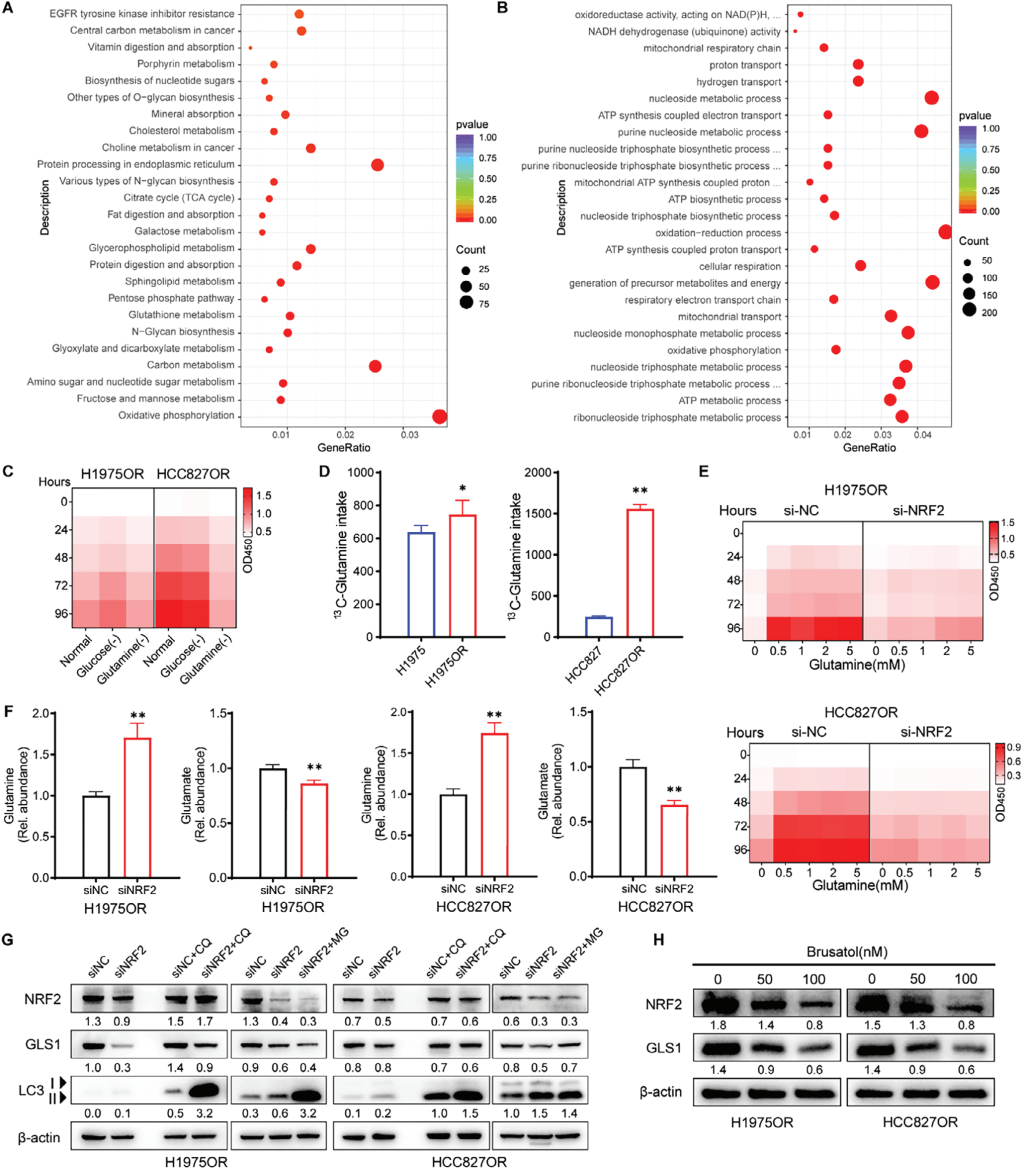
Reprogramming of glutamine metabolism in cells resistant to Osimertinib. A,B) KEGG pathway enrichment analysis (A) and GO functional enrichment analysis (B) of upregulated gene modules in HCC827OR cells (*n* = 4 per group). C,E) CCK8 assay showing cell activity in normal, glucose‐deprived, and glutamine‐deprived cultures (C), and cell activity following the knockdown of NRF2 (*E*) (*n* = per group). D,F) Quantification of glutamine uptake flux (D) and glutamine and glutamate levels post NRF2 knockdown (F) using LC‐MS/MS analysis (*n* = 5 per group). G,H) Western Blot analysis for proteins in cells treated with NRF2‐konckdown, with or without the action of MG132 and CQ (G), and proteins in cells treated with NRF2 inhibitor Brusatol (H). Data are presented as mean ± SD, calculated using two‐sided unpaired Student's *t*‐test, * *P* < 0.05 and ** *P* < 0.01.

Given the persistent challenge of secondary resistance to EGFR‐TKI in treating EGFR‐mutated NSCLC, we established drug‐resistant cell models (HCC827OR and H1975OR) for Osimertinib, as detailed in the “ Experimental Section”, to investigate targeted treatment strategies. Enrichment analysis of differentially expressed genes revealed heightened demands for synthesis and energy metabolism in resistant cells compared to parental counterparts (**Figure** **1**A,B; Figure S1A,B, Supporting Information). Subsequent deprivation experiments demonstrated that resistant cells exhibited a greater reliance on external glutamine rather than glucose for proliferation (Figure 1C; Figure S1C,D, Supporting Information). Further, glutamine uptake assays confirmed an increased capacity for ^13^C‐glutamine uptake in resistant cells (Figure 1D). Elevated levels of GLS1 and ASCT2 (SLC1A5) were also observed in resistant cells (Figure S1E,F, Supporting Information), with overexpression of these proteins linked to a higher mortality risk among lung cancer patients (Figure S1G, Supporting Information). In addition, GLS1 expression was positively correlated with EGFR expression (Figure S1H, Supporting Information), particularly within the cohort of EGFR‐mutated lung cancer patients (Figure S1I, Supporting Information). Collectively, these findings suggest a close association between reprogrammed glutamine metabolism and upregulation of key proteins in Osimertinib‐resistant cells.

Moreover, we observed a significant upregulation of NRF2 and its downstream pathways in resistant cells, which are closely associated with glutamine metabolism and acquired resistance (Figure S2A, Supporting Information),^[^ ^21^ ^]^ as well as poor prognosis in lung cancer (Figure S2B, Supporting Information). Importantly, our study revealed that NRF2 activity is not only linked to the proliferative activity of resistant cells but also to the normal expression of GLS1 protein. Genetic inhibition of NRF2 activity resulted in suppressed proliferation of resistant cell in both the glutamine‐supplemented and glutamine‐deprived conditions (Figure 1E), along with a decrease in the conversion of glutamine to glutamate (Figure 1F), suggesting that NRF2 may play a role in GLS1‐mediated glutamine hydrolysis. Further, inhibition of NRF2 activity led to the activation of cellular autophagy and significant reduction in GLS1 protein levels without notable changes at the gene level (Figure 1G,H; Figure S2C,D, Supporting Information). Further assessing autophagy flux using autophagy inhibitors and autophagy agonists, we demonstrated for the first time that activated NRF2 maintains intracellular GLS1 protein levels by inhibiting autophagy‐lysosome function (Figure 1G), and that stable GLS1 expression facilitates orderly hydrolysis of intracellular glutamine. The key regulator of glutamine metabolism c‐Myc also influences both glutamine uptake and hydrolysis by regulating ASCT2 and GLS1 expression.^[^ ^22^, ^23^ ^]^ Consequently, regulation of glutamine metabolism reprogramming by glutamine metabolism regulatory genes mainly focuses on the regulation of key proteins of the glutamine metabolic pathway. In conclusion, we propose that the dependence on glutamine exhibited by Osimertinib‐resistant cells is closely related to the functional status of their metabolic key protein.

### 2.2 Glutamine Limitation: GLS1 and ASCT2 Function Blocking Contributes to Osimertinib‐Resistant Lung Cancer Inhibition, Disrupting Energy and Redox Balance

**Figure 2:**
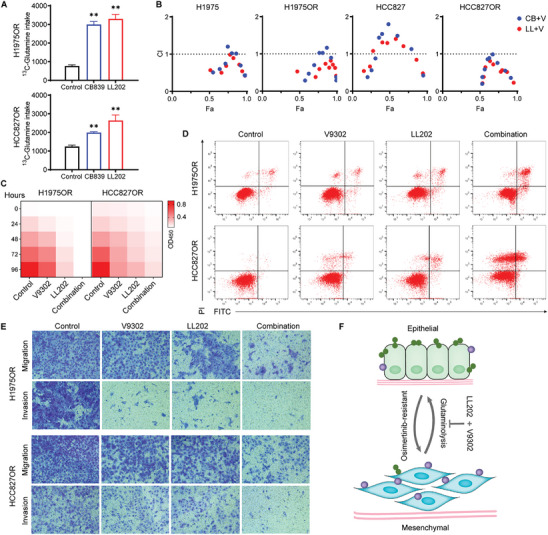
GLS1 and ASCT2 function blocking contributes Osimertinib‐resistant cells inhibition. A) Quantification of glutamine uptake subsequent to the administration of GLS1 inhibitors to drug‐resistant cells (n = 6 per group). B) Synergistic effect of GLS1 inhibitor with ASCT2 inhibitor on H1975, H1975OR, HCC827, and HCC827OR cells. CI (combination index) value was calculated as described in the Experimental Section (*n* = 10 per group). C–E) CCK8 assay (C) assessing the cellular activity, flow cytometry analysis (D) for cell apoptosis and transwell assay (scale bars, 100µm) (E) for cell migration and invasion, following V9302, LL202, and combined treatment of Osimertinib‐resistant cells (*n* = 3 per group). F) The mechanism model of GLS1 and ASCT2 dual inhibition reversing EMT in Osimertinib‐resistant cells. Data are presented as mean ± SD, calculated using two‐sided unpaired Student's *t*‐test, ** *P* < 0.01.

Subsequently, we explored the potential of targeting glutamine metabolism by modulating the function of key targets involved in this pathway to achieve a feasible anti‐tumor effect in the Osimertinib‐resistant model. First, given the limited in vivo efficacy of GLS1 inhibitors, we conducted a preliminary exploration and discovered that the exogenous glutamine uptake significantly increased following treatment with GLS1 inhibitors in resistant cells (**Figure** **2**A), leading to a certain extent of compensatory glutamine metabolism. Indeed, glutamine metabolism encompasses not only its hydrolysis via mitochondrial pathways but also the cellular uptake from the external environment. Therefore, we contemplated the potential of targeting both glutamine transport and hydrolysis. Our initial assessment of inhibitory activity confirmed that co‐targeting of GLS1 inhibitor and V9302 at a 1:1 ratio exhibited significant synergistic effects in glutamine‐dependent cells (Combination Index [CI] <1) (Table S1,2, Supporting Information). Further, the synergistic effect of LL202 (Figure S3A, Supporting Information), a novel macrolide GLS1 inhibitor developed by our group,^[^ ^13^ ^]^ combined with V9302, was significantly superior to that observed with CB839 (Figure 2B). The CI value for the LL202 group in resistant cells was below 0.6 (when Fa > 0.5) (Figure 2A; Table S3, Supporting Information), indicating strong synergy of LL202 and V9302. Similar synergistic effects were also observed in other glutamine‐dependent lung cancer cells, with the LL202 combination outperforming CB839 (Figure S3A–D, Supporting Information). Next, we assessed alterations in function and phenotype of resistant cells following treatment with LL202 and V9302 either individually or together. Compared to the monotherapy groups, combined treatment significantly suppressed cell proliferation (Figure 2C; Figure S4A–D, Supporting Information) and augmented their capacity to induce apoptosis (Figure 2D; Figure S5A,B, Supporting Information). Moreover, dual‐target inhibition markedly attenuated invasive potential of resistant cells; while, reversing epithelial‐mesenchymal transition (EMT) changes (Figure 2E,F; Figure S6A–E, Supporting Information).

**Figure 3:**
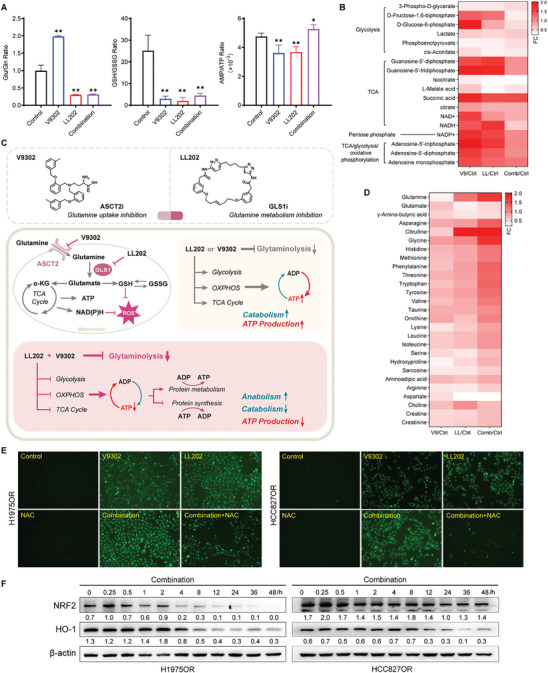
Combination therapy disrupting energy and redox balance. A,B,D) The LC/MS‐MS technique was employed to monitor alterations in metabolite levels within Osimertinib‐resistant cells following administration of combination therapy. Quantification of Glu/Gln (glutamate/glutamine) ratio, GSH/GSSG ratio, AMP/ATP ratio (A), heatmap depicting energy metabolite differences (B), and changes in free amino acids (D) (*n* = 4 per group). C) Glutamine metabolism inhibition leading to the activation of distinct metabolic pathways in both monotherapy and combination therapy. Monotherapy primarily activates energy metabolism, while combination therapy predominantly induces catabolism and suppresses anabolism. E) Fluorescence microscopic showing ROS levels of Osimertinib‐resistant cells after treating with V9302, LL202, and NAC independently or in combination was determined by DCFH‐DA staining (scale bars, 100 µm). F) Western Blot analysis for NRF2 and HO‐1 protein following time‐dependent treatment with the combination group. Data are presented as mean ± SD, calculated using two‐sided unpaired Student's *t*‐test. * *P* < 0.05 and ** *P* < 0.01.

The identification of associated metabolic changes revealed a significant inhibition of the glutamine metabolic pathway resulting from dual‐target administration (**Figure** **3**A; Figure S7D, Supporting Information). In contrast to single‐target inhibition, the glycolysis/TCA/oxidative phosphorylation pathways in the combined treatment group were not activated by stress, indicating that dual‐target inhibition concurrently disrupts energy metabolism‐related pathways (Figure 3B,C; Figure S7A,B, Supporting Information). This treatment activated the energy‐regulating switch AMPK, with an increase in the AMP/ATP ratio observed in the combined group and a decrease noted in the single‐drug group, suggesting severe energy stress induced by dual‐target inhibition in tumor cells (Figure 3C; Figure S7C, Supporting Information). Further analysis of free amino acid levels revealed a significant upregulation in the combination group (Figure 3D), implying enhanced protein degradation. These findings suggest that dual targeting induces an energy deficiency within cells, prompting activation of alternative catabolic pathways to support survival and proliferation.

Given the crucial role of glutathione (GSH), synthesized via the glutamine metabolic pathway, as a key antioxidant in maintaining redox homeostasis, we further assessed the cellular redox state. Results from fluorescence microscopy and flow cytometry demonstrated a significant increase in intracellular reactive oxygen species (ROS) following the combined treatment (Figure 3E; Figure S7E, Supporting Information). In addition, administration of the antioxidant N‐acetylcysteine (NAC) markedly reduced ROS levels in the combination group and mitigated therapy‐induced cytotoxicity (Figure S7F, Supporting Information), indicating that ROS generation contributes to the cytotoxic effects mediated by LL202 and V9302. Further, there was a significant downregulation of NRF2 and its downstream target HO‐1 following combined treatment (Figure 3F), indicating a lack of reactive activation of the antioxidant pathway. These findings indicate that dual‐target inhibition enhances protein catabolism for energy provision while decreasing NRF2 activity within cells (Figure S7G, Supporting Information). Assessments of autophagic flux using chloroquine (CQ) as an autophagy inhibitor and MG132 as an autophagy activator confirmed that dual‐target inhibition suppresses NRF2/ARE‐mediated antioxidant pathway activation through autophagy (Figure S7H, Supporting Information). In conclusion, the dual‐target inhibition of glutamine disrupts energy homeostasis, concomitantly inducing ROS production and suppressing activation of the NRF2/ARE antioxidant pathway, thereby further perturbing cellular redox balance and enhancing anti‐tumor efficacy.

### 2.3 Combination Therapy Demonstrates Remarkable Efficacy in Preclinical Models

**Figure 4:**
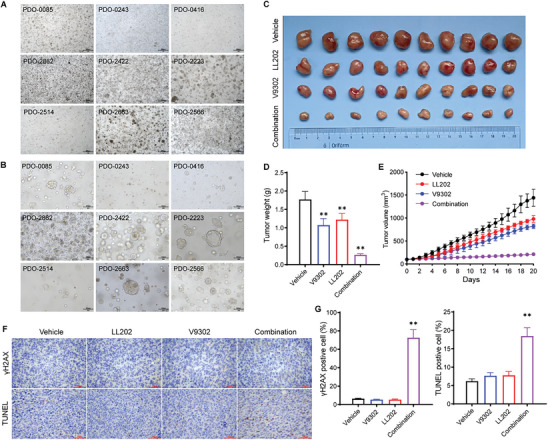
Combination therapy demonstrates superior efficacy in preclinical models. A) Microscopic showing the morphology of PDOs (scale bars, 500 µm). B) Microscopic showing the morphology of PDOs (scale bars, 100 µm). C–G) H1975OR‐tumor bearing mice were treated with normal saline (Vehicle), LL202 (30 mg kg^−1^), V9302 (30 mg kg^−1^), and LL202+V9302 (30 mg kg^−1^). Tumors collected at the conclusion of the specified treatments, representative image depicting the tumors at the end of the treatment period is presented (C); quantification of tumor weight (D) and tumor volume (E); representative images of γH2AX positive cells and TUNEL positive cells in tumors (scale bars, 50 µm) (F), and quantification of γH2AX positive cells and TUNEL positive cells (G) (*n* = 10 per group). Data are presented as mean ± SD, calculated using two‐sided unpaired Student's *t*‐test. * *P* < 0.05 and ** *P* < 0.01.

We evaluated the anti‐tumor efficacy of the combination treatment across various preclinical models, including 3D tumor cell models, patient‐derived organoid (PDO) models, and xenograft tumor models. In the resistant 3D cell model, we observed a dose‐dependent decrease in cellular activity with increasing concentrations of combination therapy (Figure S8A–C, Supporting Information). Conversely, activity decreased in 3D‐HCC827OR compared to 2D cells. To assess clinical relevance, we established nine lung cancer PDO models using tumor tissue samples from patients with sequencing information and medication history (**Figure** **4**A,B). In PDOs with EGFR‐activating mutations resistant to Osimertinib, combination therapy demonstrated greater inhibition of proliferation than Osimertinib‐resistant 3D cell model (Figure S8D,E and Table S4, Supporting Information). Similarly, it inhibited cell proliferation in an ALK‐mutant PDO model without prior drug exposure. Notably, our LL202 plus V9302 combination showed equal efficacy in a multi‐drug resistance model that included chemotherapy, targeted therapy, and immunotherapy. The H1975OR xenograft model exhibited a significant reduction in tumor, achieving an impressive 85.18% inhibition rate compared to monotherapy (Figure 4; Figure S9A,B, Supporting Information). Further, there was a substantial increase in γH2AX expression level and TUNEL‐positive cells in the combination group (Figure 4F,G). Importantly, no evidence of in vivo toxicity was observed through monitoring serum ALT/AST and BUN/Cr levels (Figure S9C, Supporting Information), as well as HE staining of vital organs (Figure S9D, Supporting Information). In summary, the combined targeted therapy demonstrated both efficacy and notable safety.

### 2.4 Glutamine Substitution: Design of NQO1‐Activatable DON Prodrugs, and the Discovery of 10e

**Figure 5:**
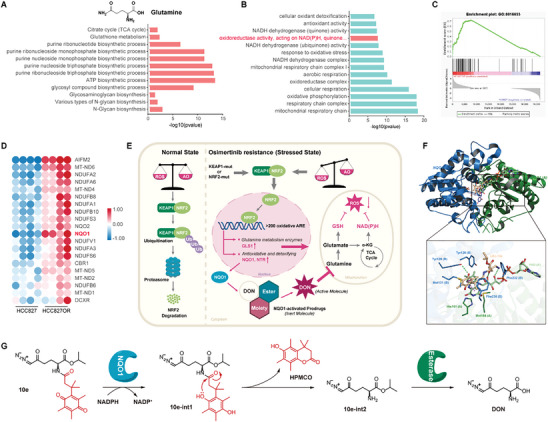
Design of NQO1‐activatable DON prodrugs. A) Enrichment analysis of pathway and process closely associated with glutamine in HCC827OR cells. B) Enrichment analysis of pathways and processes associated with redox‐reduction. C) GSEA enrichment analysis for GO:0016655 (oxidoreductase activity, acting on NAD(P)H, quinone, or similar compound as acceptor). D) Differential genes in GO:0016655 (*n* = 4 per group). E) Schematic illustration of the design strategy and the mechanism of action of prodrugs. F) Molecular docking model of NQO1 (PDB ID: 3JSX) with 10e. G) Theoretical mechanism for the liberation of the bioactive molecule DON from 10e through co‐activation of NQO1 and esterase.

The anti‐tumor effect of the LL202 plus V9302 combination treatment was unsatisfactory in the HCC827OR xenograft model (results not shown), which is characterized by high energy metabolism. Consequently, we speculate that dual‐target blockade is insufficient to inhibit in vivo metabolism in this hypermetabolic tumor model. Does the lack of efficacy indicate uncertainty regarding glutamine‐targeting strategies? Notably, the pathways and processes associated with the glutamine metabolic network are significantly enriched in this model (**Figure** **5**A), suggesting that glutamine metabolism is essential for tumor progression. Therefore, we propose shifting our antagonistic strategy toward a broad‐spectrum antagonist capable of comprehensively inhibiting glutamine metabolism. The most potent broad‐spectrum antagonist identified in current research is the natural product DON, which exhibits structural similarities to glutamine, selectively and irreversibly covalently binding to glutamine‐utilizing enzymes (GUZs). This binding release the diazo group and forms the enzyme‐DON complex, effectively inhibiting GUZs activity and disrupting the entire tumor metabolic network. Although it progressed to phase II clinical trials, its development has been suspended due to suboptimal tumor targeting and safety issues leading to gastrointestinal toxicity. As a result, recent investigations have concentrated on modifying this compound to enhance its efficacy.^[^ ^17^, ^24^ ^]^

Further enrichment analysis of the resistance model revealed a significant enhancement of its antioxidant defense system (Figure 5B). Additionally, key oxidoreductase activities were markedly elevated, including those associated with NAD(P)H and quinone receptors (Figure 5C). NQO1 is a flavoenzyme that catalyzes quinone reduction through a two‐electron mechanism in the presence of NAD(P)H, exerting detoxifying or bioactivating effects. While NQO1 is constitutively expressed at low levels in normal tissues, it is overexpressed in tumor tissues and particularly activated in Osimertinib‐resistant cells (Figure 5D; Figure S2A, Supporting Information). To enhance the safety and efficacy of DON, we synthesized a novel series of NQO1‐activatable prodrugs for targeting by leveraging the differential expression between tumor and normal tissues (Figure 5E). Trimethyl lock, a common NQO1 substrate,^[^ ^25^ ^]^ was used as a protecting group conjugated with the amino moiety of DON. The carboxyl moiety of DON, meanwhile, was shielded by esterification, which is similar to DRP104. By modifying substituents on benzoquinone and introducing diverse ester linkages, we obtained eight NQO1‐responsive DON prodrugs.

The synthesis and characterization of the designed NQO1‐activatable DON prodrugs (10a–10h) are illustrated in Scheme S1, Supporting Information. The catalytic efficacy of prodrugs is detailed in Table S5, Supporting Information. Prodrug 10e exhibited favorable metabolic stability and accelerated NQO1‐mediated reduction, leading to its selection for further assessment. Docking results indicated that 10e specifically bound to the shallow catalytic pocket, which consisted of Met154 and His161 from one monomer and Tyr126, Tyr128, Met131, Phe232, and Phe236 from the opposing NQO1, with the benzoquinone aligned parallel to the cofactor FAD, forming π–π stacking interactions between both benzene and pyrimidine diketones within the ternary condensed ring of FAD (Figure 5F). Notably, the carbonyl group of benzoquinone served as a H‐bond acceptor to contact with Tyr126; while, the ketone and terminal diazo group interacted with FAD through H‐bonds, providing further insights into the reactivity of 10e toward NQO1. Subsequently, HPLC analysis was employed to assess the release of prodrug 10e in the presence of NQO1 and cofactor NADPH; it was observed that nearly 80% of 10e was depleted within just 10 min when exposed to NQO1; while, remaining stable in buffer at pH 7.4 in its absence (Figure S10A, Supporting Information).

Table S5, Supporting Information presents the stability of the highly metabolized site (plasma) and the toxicity site (intestine). Under experimental conditions, compound 10e demonstrates considerable stability in human plasma and intestine, with over 90% of the tested molecules remaining after 1 h. In rodent plasma, rich in esterase,^[^ ^17^ ^]^ the ester moiety of the prodrug undergoes hydrolysis into carboxylic acids. However, the resulting metabolic intermediate, 10e‐int, remains stable in plasma for over an hour (Figure S10B, Supporting Information). DON is detected only after further co‐incubation with NQO1 and NADPH, showing a time‐dependent increase and reaching its maximum release after 2 h (Figure S10C, Supporting Information). These results indicate that NQO1‐activatable DON prodrugs exhibit minimal metabolism in both plasma and the gastrointestinal tract, suggesting a favorable safety profile due to reduced conversion to DON. Further, in vivo acute toxicity experiments reveal no significant changes in liver transaminase levels and jejunal histology in the 10e group compared to DON treatment (Figure S11A–C, Supporting Information), indicating superior in vivo safety of 10e relative to DON.

### 2.5 Tumor Targeting and Anti‐Tumor Activity of 10e

To evaluate the tumor‐targeting properties of 10e, we assessed its ability to permeate and cleave to DON in tumor cells using tumor‐plasma model system. 10e exhibited similar DON partitioning and biotransformation profiles as parental DON drug in tumor cells; while causing minimal release of DON in human plasma (Figure S12A, Supporting Information). Further, the distribution ratio of 10e between tumor cells and plasma was significantly higher than that of DON itself. Importantly, in the in vivo tumor model, 10e released a greater amount of DON in tumor tissue than plasma (Figure S12B, Supporting Information). Metabolite identification revealed that 10e was directly reduced in tumor cells to form lactone and DON isopropyl ester (10e‐int2), which was subsequently hydrolyzed to form DON (Figure 5C; Figure S12C, Supporting Information).

**Figure 6:**
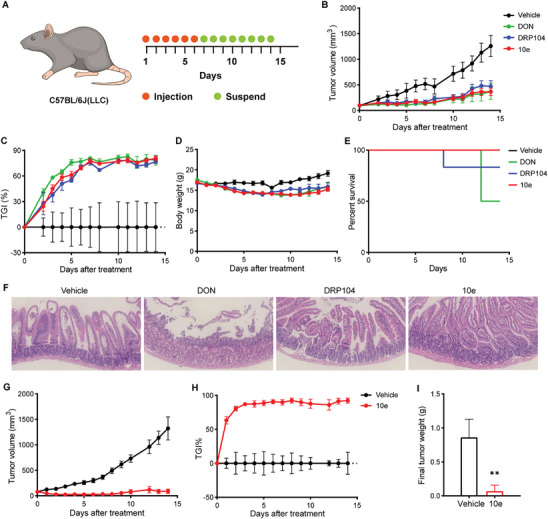
In vivo evaluation of the antitumor efficacy and safety profile of optimized prodrug 10e. A–F) Comparison of the efficacy and toxicity of equimolar DON, DRP104, and 10e (0.5 mg kg^−1^ DON equiv.) in LLC tumor‐bearing mice. C57BL/6J mice were inoculated with 5 × 10^5^ cells, group administration according to the time points shown in the schematic diagram (A), quantification of tumor volume (B), TGI (C), body weight (D), and percent survival (E) during the specified treatments period, and HE staining of the intestines (original magnification, 20×) (F) at the conclusion of the specified treatments (*n* = 6 per group). G–I) The efficacy and toxicity of 10e (0.5 mg kg^−1^ DON equiv.) in MC38 tumor‐bearing mice. Quantification of tumor volume (G) and TGI (H) during the specified treatments period and tumor weight (I) at the conclusion of the specified treatments (*n* = 5 per group). Data are presented as mean ± SD, calculated using two‐sided unpaired Student's *t*‐test. * *P* < 0.05 and ** *P* < 0.01. Data in (E) were calculated using log‐rank test.

Next, we conducted a comparative analysis of the efficacy and safety of DON, DRP104, and 10e in C57BL/6J mice bearing LLC tumors. The mice received daily injections of vehicle, DON (0.5 mg kg^−1^), DRP104 (0.5 mg kg^−1^ DON equiv.), and 10e (0.5 mg kg^−1^ DON equiv.), respectively. Following 6 days of continuous subcutaneous administration (**Figure** **6**A), 10e exhibited comparable antitumor efficacy to both DON and DRP104. Further, this efficacy was sustained even after discontinuation (Figure 6B,C; Figure S13A,C, Supporting Information). Although all groups experienced weight loss during treatment, the mice in the DRP104 and 10e groups showed significant recovery to normal levels during the discontinuation phase (Figure 6D; Figure S13B, Supporting Information). In addition, gastrointestinal histological results indicated improvements in both the 10e group and the DRP104 group compared to the DON group (Figure 6F). Notably, three mice in the DON group died on day 12 of treatment, and one mouse in the DRP104 group died on day 8 of treatment; conversely, all mice in the 10e group survived until the experiment's endpoint (Figure 6E). Further, in a murine model of MC38 tumor, 10e (0.5 mg kg^−1^ DON equiv.) demonstrated superior antitumor efficacy (Figure 6G–I; Figure S13D–H, Supporting Information). After 6 days of continuous administration, tumor inhibition rate reached up to 90% with persistent anti‐tumor effects observed until experimental endpoints following cessation of administration. Despite initial weight loss during treatment, it returned to baseline levels at later stages (Figure S13G,H, Supporting Information); while, gastroenteromics analysis indicated negligible systemic toxicity associated with 10e throughout treatment (Figure S13F, Supporting Information). These findings suggest that the in vivo antitumor efficacy of 10e is comparable to DRP104 and DON but exhibits a significantly improved safety profile relative to DON.

### 2.6 Prominent Anti‐Tumor Efficacy of 10e in Immunodeficiency Models with NQO1 Hyperactivation

**Figure 7:**
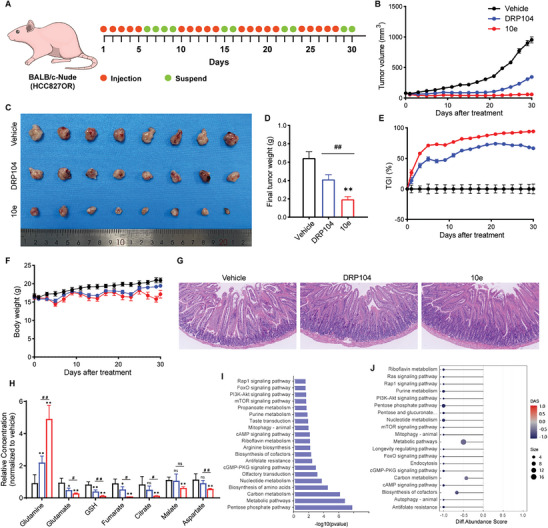
Comparison of the efficacy, toxicity, and metabolism/flow of equimolar DRP104 and 10e in the NQO1‐activated hypermetabolic tumor model. A–G) Comparison of the efficacy and toxicity of equimolar DRP104 and 10e (0.4 mg kg^−1^ DON equiv.) in HCC827OR tumor‐bearing mice. BALB/c‐nude mice were inoculated with 5 × 10^6^ cells, group administration was carried out in accordance with the time points shown in the schematic diagram (A); tumors collected at the conclusion of the specified treatments, representative image depicting the tumors at the end of the treatment period is presented (C); quantification of tumor volume (B); TGI (E); and body weight (F) throughout the specified treatments period, as well as measurement of final tumor weight (D) and HE staining of the intestines (original magnification, 20×) (G) conducted at the conclusion of the specified treatments (*n* = 8 per group). H–J) The LC‐MS/MS technique was employed to monitor alterations in metabolite levels within HCC827OR tumors at the conclusion of DRP104 and 10e treatment. Comparison of glutamine metabolism and TCA cycle components (H), KEGG enrichment pathway map (10e vs. DRP104) (I), differential abundance score (DAS) plots of all enriched metabolic pathways (The DAS represents the comprehensive change in all metabolites within the metabolic pathway. A score of 1 indicates up‐regulation of all identified metabolites in the pathway, while a score of −1 indicates down‐regulation) (10e vs. DRP104) (J) (*n* = 4 per group). Data are presented as mean ± SD, calculated using two‐sided unpaired Student's *t*‐test. * *P* < 0.05, ** *P* < 0.01, ^#^ *P* < 0.05, ^##^ *P* < 0.01, ns not significant, and *P* > 0.05.

We subsequently assessed the anti‐tumor efficacy of 10e in the NQO1‐hyperactivated HCC827OR transplantation tumor model. In the subcutaneous transplantation tumor model, 10e (0.4 mg kg^−1^ DON equiv.) and DRP104 (0.4 mg kg^−1^ DON equiv.) were administered via subcutaneous injection for 5 days per cycle over 4 cycles (**Figure** **7**A). During the initial treatment cycle, all mice experienced ≈15% weight loss. In subsequent cycles, weight loss did not exceed 10%, and the mice recovered to above normal levels during withdrawal periods (Figure 7F; Figure S14A, Supporting Information). Gastroenteromics also showed normal morphology (Figure 7G). These findings indicate minimal systemic toxicity in immunodeficient mice across both treatment cohorts. At the experimental endpoint, the tumor suppression rate in the 10e group reached 94.25%, significantly higher than the 66.48% observed in DRP014 group (Figure 7E). In addition, the relative tumor volume (RTV) was 0.805 in the 10e group compared to 4.421 in the DRP104 group, demonstrating markedly superior inhibition of tumor growth by 10e (Figure 7B–E; Figure S14B, Supporting Information). What's more, significant tumor growth was detected during the fourth treatment cycle in the DRP104 group; while, no substantial change was observed in the 10e group, suggesting that DRP104 may lead to earlier development of resistance. These results confirm the prominent anti‐tumor efficacy of 10e.

Subsequently, we examined the effects of DRP104 and 10e treatments on tumor metabolism. Both treatments significantly suppressed tumor glutamine metabolism, as evidenced by increased glutamine accumulation and decreased levels of metabolites such as glutamate and GSH, along with reduced levels of TCA cycle intermediates derived from glutamine including fumarate, citrate, aspartate, and malate (Figure 7H). Notably, 10e induced more pronounced alterations in these metabolite levels compared to DRP104. A comprehensive analysis of other key energy metabolites and a differential analysis of the detected metabolites were performed (Figure 7I,J; Figure S15A–G, Supporting Information). Treatment with DRP104 resulted in the downregulation of certain energy metabolite (Figure S15A, Supporting Information); while, 10e led to downregulation of a greater number of energy metabolites (Figure S15B, Supporting Information), with significant differences observed in the differential metabolite profiles between 10e and DRP104 groups (Figure S15C, Supporting Information). In addition, KEGG differential enrichment analysis revealed that compared to DRP104, 10e induced more extensive downregulation of metabolic pathways, including amino acid metabolism, nucleotide metabolism, and carbon metabolism, as well as signaling pathways such as mTOR and PI3K‐AKT (Figure 7I,J; Figure S15D‐G, Supporting Information). These results demonstrate that 10e exerts broad inhibitory effects on tumor metabolism, underscoring its anti‐tumor advantages, particularly in highly metabolic tumors.

### 2.7 Compound 10e Promotes Anti‐Tumor Microenvironment in Immunosuppressive/Immunoinvasive Tumors

Through a comprehensive investigation of the HCC827OR model, we observed a reduction in the activation and positive regulation of the immune response (Figure S16A, Supporting Information), as well as the absence of PD‐L1 immune checkpoint expression (Figure S16C,D, Supporting Information). Co‐culture experiments with immune cells revealed that PD‐L1‐deficient HCC827OR cells induced immune cell infiltration, but without detectable tumor reactivity or significant tumor cell apoptosis in infiltrated immune cells (Figures S17A–D and S18A–C, Supporting Information), indicating an immunologically “cold” microenvironment and challenging prospects for immunotherapy.^[^ ^26^, ^27^ ^]^ Further, there was enhanced regulation of M2‐type macrophage activation (Figure S16B, Supporting Information),^[^ ^28^ ^]^ leading to a significant shift from M0 to M2 macrophages and promoting a pro‐tumor environment (Figure S19A, Supporting Information).

**Figure 8:**
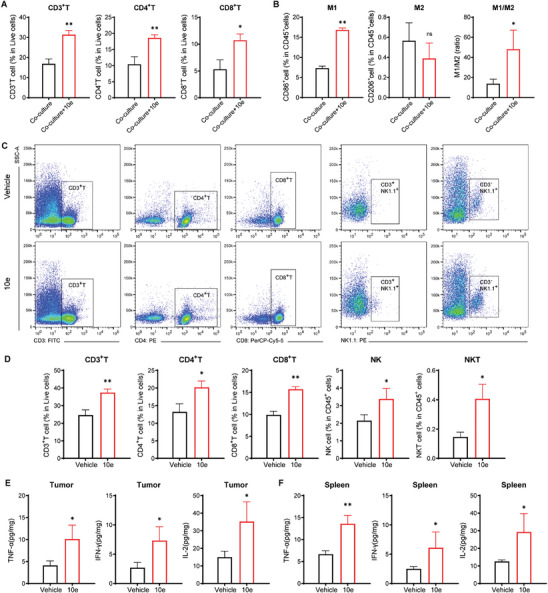
10e treatment promotes an anti‐tumor microenvironment in immunosuppressive/ immunoinvasive tumors. A,B) Flow cytometry analysis was performed to identify immune cell subpopulations following co‐culture of HCC827OR cells with immune cells, and quantification of cytotoxic T cells (CD3^+^T, CD4^+^T, and CD8^+^T) (A) and macrophages (M1, M2, and M1/M2 ratio) (B) (*n* = 3 per group). C,D) MC38‐bearing mice treated with 10e (0.5 mg kg^−1^ DON equiv., subcutaneously, 6 days), representative flow cytometry plots (C), and data charts (D) showing CD3^+^T, CD4^+^T, CD8^+^T, NK, and NKT cells subsets and ratios (*n* = 3 per group). E,F) The ELISA assay was employed to monitor alterations in key cytokines within tumors (E) and spleens (F) of MC38 tumor‐bearing mice following treatment with 10e (*n*  =  4 per group). Data are presented as mean ± SD, calculated using two‐sided unpaired Student's *t*‐test, * *P* < 0.05, ** *P* < 0.01, ns not significant, and *P* > 0.05.

Next, we investigated the potential of 10e to modulate the suppressive immune microenvironment. Following 10e treatment in a co‐culture system, HCC827OR cell morphology exhibited significant alterations, including compromised cell membrane integrity and irregular shape (Figure S18A,B, Supporting Information). Analysis of lysogenic mediators in the co‐culture environment revealed a marked increase in levels of anti‐tumor cytokines IL‐2, IL‐12, IFN‐γ, and TNF‐α, accompanied by significant down‐regulation of pro‐tumor cytokines IL‐4, IL‐6, and IL‐10 (Figure S18C, Supporting Information). Further, treatment with 10e induced activation of cytotoxic T lymphocytes such as CD3^+^T, CD4^+^T, and CD8^+^T cells (**Figure** **8**A; Figure S20A, Supporting Information). Evaluation of tumor‐associated macrophages based on M1 (CD86^hi^iNOS^hi^), M2 (CD206^hi^ARG1^hi^) typing, as well as M1/M2 ratios (Figure 8B; Figures S19A and S20B, Supporting Information) demonstrated that 10e inhibited the polarization of M2‐type macrophages; while, promoting their transformation into M1‐type macrophages. Notably, there was a significant up‐regulation of HLA‐I (HLA‐A, HLA‐B, and HLA‐C) antigens following exposure to 10e (Figure S19B, Supporting Information), which is crucial given that loss of HLA class I genotype can render tumors unresponsive to immunotherapies due to evasion from immunosurveillance mechanisms.^[^ ^29^ ^]^

To further elucidate the immunoregulatory mechanism of 10e in immune‐infiltrating tumors, we conducted immunocyte typing analysis on MC38 mice treated with 10e (0.5 mg kg^−1^ DON equiv.). Consistent with the findings regarding 10e in immunosuppressive tumors, a significant disparity in immunocyte typing was observed between treated and untreated mice. Flow cytometry results revealed that 10e treatment markedly elevated the proportion of cytotoxic killer immune cells, including CD3^+^T, CD4^+^T, CD8^+^T, NKT, and NK (Figure 8C,D), and demonstrated heightened proliferative activity of CD4^+^T, CD8^+^T, and NKT in the treated group (Figure S21A,B, Supporting Information). Notably, a substantial upregulation of effector memory T cell subsets within the population of CD8^+^ memory T cells was also observed (Figure S22A,B, Supporting Information). In contrast to central memory T cells primarily situated in lymphoid organs, effector memory T cells are predominantly located in peripheral tissues and can promptly generate effector cytokines upon antigenic stimulation. ELISA results demonstrated a significant increase in the levels of anti‐tumor cytokines IFN‐γ, TNF‐α, and IL‐2 following treatment with 10e (Figure 8E,F). Further, no notable alteration was observed in immunosuppressive regulatory T (Treg) cell populations (Figure S22C, Supporting Information); while, there was a marked reduction in pro‐tumor myeloid‐derived suppressor cell (MDSC) content (Figure S22D, Supporting Information), suggesting suppression of pro‐tumor immune response. In summary, the aforementioned results indicate that 10e can substantially enhance the tumor immune microenvironment and bolster anti‐tumor immune response.

## 3 Discussion

Our findings imply that targeting glutamine metabolism may provide a viable therapeutic alternative for Osimertinib‐resistant NSCLC. Conventionally, strategies to address acquired resistance in NSCLC have focused on precision medicine, tailoring treatment regimens based on the evolving nature of resistance. While somewhat effective, this strategy frequently disregards the metabolic adaptations of cancer cells and fails to tackle the fundamental cause of the issue of addressing acquired resistance. Indeed, glutamine assumes a crucial role in the metabolism of malignant tumors, influencing tumor progression through interactions with the tumor microenvironment and contributing to drug resistance and recurrence.^[^ ^30^, ^31^, ^32^ ^]^

Our research manifestly demonstrated that the Osimertinib‐resistant NSCLC model manifests an exceedingly high anabolic activity and energy demands, presenting a distinct reliance on glutamine metabolism. We also detected the upregulation of GLS1 and ASCT2, crucial proteins in glutamine metabolism, within the models. Further, we debuted the presentation that NRF2,^[^ ^21^, ^32^ ^]^ a regulator intimately correlated with glutamine metabolism, can modulate glutamine metabolism by restraining autophagic lysosomal function to preserve GLS1 protein levels. This finding aligns with the regulatory mechanism of c‐Myc, which governs glutamine uptake and hydrolysis through modulation of ASCT2 and GLS1 expression levels.^[^ ^22^, ^23^ ^]^ Our findings accentuated that the glutamine dependence in Osimertinib‐resistant cells is intimately affiliated with the functional status of proteins associated with the glutamine metabolic pathway. Consequently, we initially contemplated targeting glutamine metabolic pathway‐related targets.

It is a given that recent studies on tumor glutamine metabolism have concentrated on the development of GLS1 allosteric inhibitors, to which our research group has contributed several works.^[^ ^13^, ^33^ ^]^ These inhibitors exhibit high selectivity and safety and reveal promising antitumor activities in vitro; however, achieving limited progress in vivo and in the clinical setting. Therefore, the current study investigates the inconsistent activities of GLS1 inhibitors observed in in vitro and in vivo studies and proposes a novel approach: simultaneous targeting of tumor glutamine transport (ASCT2) and glutamine metabolism (GLS1). We hypothesize that this dual‐targeting strategy might generate a synergistic effect, significantly enhancing anti‐tumor efficacy (1 + 1 > 2). Our initial investigations confirm the synergistic benefits of combining LL202 and V9302 over the combination of CB839 and V9302, as evidenced by cellular activity assays and CI values. Through comprehensive evaluations both in vitro and preclinical models, we determine that the LL202 and V9302 combination effectively inhibits tumor cell proliferation, promotes apoptosis, reduces invasiveness, and induces energy depletion along with oxidative stress. This combination therapy also proves effective across various drug‐resistant organoid models, including those resistant to targeted therapy, immunotherapy, and chemotherapy. Moreover, in Osimertinib‐resistant transplanted tumor models, this combination not only demonstrated significant efficacy but also maintained an excellent safety profile. Consequently, our study validates and substantiates the feasibility of this novel dual‐targeting approach to enhance the in vivo efficacy of GLS1 allosteric inhibitors.

Based on the concept of enhanced efficacy via dual targeting of GLS1 and ASCT2, we further explored if comprehensive inhibition of all GUZs or broad‐spectrum glutamine metabolism antagonists would surpass the efficacy of selective GLS1 inhibitors in a clinical context. Distinct from the DON prodrug design concept of DRP104 relying on Cathepsin L hydrolysis,^[^ ^17^, ^18^, ^19^, ^20^ ^]^ this study further proposed to capitalize on the elevated redox level in the oxidative reduction within Osimertinib‐resistant cell lines and designed the antioxidant factor NQO1‐responsive DON prodrug molecules. This design concept aimed to leverage the unique metabolic and genetic milieu of cancer cells to enhance therapeutic precision and efficacy. Through a comprehensive assessment of stability, tumor targeting, and safety, we identified the optimal NQO1‐responsive DON prodrug, 10e, a molecule that demonstrated potent antitumor activity in diverse ex vivo and in vivo scenarios and exceeded the efficacy of the clinical phase II drug, DRP104, in the Osimertinib‐resistant malignant tumor model. Given the potent antitumor effect and the vigorous metabolism of Osimertinib‐resistant cells in tumors, we further evaluated its impact on tumor metabolism. The results indicated that 10e not only disrupted the glutamine metabolic network but also partially influenced glucose and lipid metabolism in tumor tissues, and the effect was more prominent than that of DRP104. Hence, our results imply that 10e holds a therapeutic advantage over DRP104 in hypermetabolic tumors.

The interactions between tumor cells and their microenvironment are of critical significance for the cancer phenotype, disease progression, and therapeutic response.^[^ ^34^, ^35^ ^]^ DON and its prodrugs, JHU083 and DRP104, have been demonstrated to be efficient in improving the tumor microenvironment, thereby generating superior antitumor activity.^[^ ^16^, ^17^, ^24^ ^]^ Our findings also suggest that 10e activates cytotoxic immune cells and elicits a protective immune response. Currently, the modulation of the tumor immune microenvironment by DON prodrugs is mainly centered on immune‐infiltrating tumor models,^[^ ^17^, ^24^ ^]^ while in this investigation, we not only explored immune‐infiltrating tumors but also concentrated on the effect of 10e on the Osimertinib‐resistant model of an immunologically “cold” microenvironment. Metabolic reprogramming, coupled with PD‐L1 deletion, elevated levels of IL‐6, and extensive infiltration of tumor‐associated macrophages results in a cold immune phenotype in Osimertinib‐resistant models, which complicates the efficacy of immunotherapy.^[^ ^26^, ^36^, ^37^, ^38^, ^39^ ^]^ Interestingly, our results reveal that 10e not only promotes the activation of cytotoxic killer immune cells in this tumor microenvironment but also reverses the infiltration proportion of M2/M1‐type macrophages. Thus, our research herein could manifest that 10e notably ameliorates the “cold” immune microenvironment and potentiates the anti‐tumor immune response, thereby potentially resolving the immunotherapy conundrum.

## 4 Conclusion

In summary, our findings reinforce the concept of targeting rewired metabolism in cancer, particularly in addressing Osimertinib‐resistant NSCLC. The compound 10e demonstrates significant promise in the context, highlighting the potential to achieve a favorable therapeutic index. Given the complexity of glutamine metabolism in cancer, a comprehensive inhibition strategy that targets multiple nodes, such as ASCT2 and GLS1, or a broad inhibition is essential to prevent rapid metabolic rewiring. Mechanism‐based prodrug design holds the potential to expand the therapeutic window and enhance the clinical application of anti‐tumor drugs targeting tumor glutamine metabolism.

## 5 Experimental Section

### Chemical Synthesis

Details of the synthesis of the compounds and their intermediates are elaborated in the Supporting Information.

### Reagents

LL202, 10e were obtained from our laboratory with a purity > 98%. DON (HY‐108357), DRP104 (HY‐132832), CB‐839 (HY‐12248), V9302 (HY‐112683), NAC (HY‐B0215), Brusatol (HY‐19543), Chloroquine (HY‐17589A), Osimertinib (HY‐15772), MG‐132 (HY‐13259), L‐Glutamine (HY‐N0390), L‐Glutamic acid (HY‐14608), and GSH (HY‐D0187) were obtained from MCE. ^13^C‐L‐Glutamine (IR‐73028) was obtained from Shanghai Zzbio. L‐Glutamine solution (R27053) was obtained from Shanghai yuanye Bio‐Technology. NADPH (BD123569) was obtained from Shanghai Bide Pharmatech.

### Cell Culture

16HBE, A549, H596, H1299, PC9, H1975, HCC827, LLC, and MC38 cells were purchased from the National Collection of Authenticated Cell Cultures. H1975OR and HCC827OR were self‐induced Osimertinib‐resistant cells in the authors’ laboratory from H1975 and HCC827 by increasing initial concentration(IC_50_ value) gradually. All cells were cultured under standard culture conditions (37 °C, 5% CO_2_) in the culture medium recommended by the ATCC. None of the cells were contaminated by mycoplasma.

### Tumor Cell‐Immune Cell Co‐Culture

With respect to T‐cell activation, Anti‐CD3 antibody was diluted in sterile PBS (1 µg mL^−1^), and the diluted antibody was introduced into a T25 culture flask. The flasks were incubated at 5% CO_2_, 37 °C for 2 h. Then, the anti‐CD3 solution was aspirated, and the Jurkat T cells were added to the CD3‐activated flasks concurrently with 5 µg mL^−1^ of anti‐CD28; the Jurkat T cells were incubated at 5% CO_2_, 37 °C for 48–72 h.

The tumor cells were inoculated in 6‐well plates overnight, and subsequently, co‐cultured directly with pre‐activated Jurkat T cells at a 1:1 ratio. After 72 h, the tumor cells were collected and analyzed for apoptosis using Annexin V‐FITC/PI in a flow cytometer. Pre‐inoculated tumor cells were directly co‐cultured with pre‐activated Jurkat T cells at a 1:1 ratio for 72 h (treated or untreated with 10e), following which, the cell morphology was observed under the microscope (Nikon ECLIPSE Ts2). Eventually, T cells were collected and qPCR was carried out to detect the levels of relevant cytokines, respectively.

Fresh mouse spleens were harvested in an aseptic setting, adding 5 mL of sterile PBS, ground, and filtered. The cells were centrifuged at 1000 rpm for 5 min at 4 °C, the supernatant was disposed of, and the cells were resuspended in 2–5 mL of pre‐chilled 1× RBC lysate for 5 min. Then, the cells were resuspended in 10 mL PBS, centrifuged at 1000 rpm for 5 min at 4 °C, after which the cells were re‐suspended in PRMI‐1640 medium. Next, the suspended splenocytes were directly co‐cultured with pre‐inoculated tumor cells. After 72h of culturing (treated or untreated with 10e), splenocytes were collected and assessed by flow cytometry (FACSCelesta) for immune cell typing.

THP‐1 cells were inoculated into 6‐well plates and treated with 100 ng mL^−1^ PMA. The cells were incubated at 5% CO_2_ and 37 °C for 24 h. After incubation, the supernatant was discarded and replaced with PRMI‐1640 medium. Once the cells reached ≈80% confluence, culture chambers were placed in the 6‐well plates, and tumor cells were added to the upper layer. The culture was continued for 48–72 h with or without 10e treatment. Subsequently, macrophages from the lower layer were collected. qPCR was employed to detect macrophage markers and cytokines, and flow cytometry (FACSCelesta) was utilized to analyze macrophage typing.

### Western Blot

Cell samples were lyzed using RIPA buffer supplemented with protein phosphatase inhibitor and protease inhibitor. The lysates were separated by SDS‐PAGE and transferred to polyvinylidene fluoride (PVDF) membrane. The membrane was blocked with 5% nonfat milk, and then, incubated with diluted primary antibodies at 4 °C for 12 h and washed with 1× TBST; the membrane was incubated with a secondary antibody for 1 h at room temperature. The immune response bands were visualized using enhanced chemiluminescent reagents (Tanon). The primary antibodies used are listed in Table S7, Supporting Information.

### Quantitative Real‐Time PCR

Total ribonucleic acid (RNA) was extracted from cells using RNA isolater Total RNA Extraction Reagent (Vazyme) according to the manufacturer's instructions. RNA concentration and purity were measured by One Drop spectrophotometer (Wuyi Technology) to ensure the quality of RNA. RNA was reverse transcribed into complementary deoxyribonucleic acid (cDNA) using the Hiscript Q RT SuperMix for qPCR (+gDNA wiper) Kit (Vazyme). Quantitative analysis was performed using Taq pro Universal SYBR qPCR Master Mix Kit (Vazyme) and QuantStudio 3 real‐time PCR instrument (Thermo Fisher Scientific). Relative mRNA levels were standardized to the β‐actin mRNA levels. The primer sequence information is listed in Table S8, Supporting Information.

### Transcriptomics

Total RNA samples were extracted using an RNA Extraction Kit (Takara) according to the manufacturer's instructions. Total amounts and integrity of RNA were assessed using the RNA Nano 6000 Assay Kit of the Bioanalyzer 2100 system (Agilent Technologies). Libraries were sequenced on an Illumina HiSeq 6000 platform. After quality control of raw reads, the clean reads were mapped to the human genome using default parameters. Differentially expressed gene (DEG)–normalized read counts (fragments per kilobase of exon per million [FPKM]) were calculated using feature Count (v1.5.0‐p3). The clusterProfiler R package (3.8.1) was used to test the statistical enrichment of differentially expressed genes in the Reactome pathway, Kyoto Encyclopedia of Genes and Genomes (KEGG) pathways, and Gene Ontology (GO) pathway. KEGG pathways and GO were annotated using the KEGG pathway database (http://www.genome.jp/kegg/) and GO Database (http://www.geneontology.org/), respectively.

### Liquid Chromatography–Mass Spectrometry/Mass Spectrometry (LC‐MS/MS)

LC‐MS/MS system (SHIMADZU) was used to measure the ^13^C‐L‐glutamine uptake content and metabolite content in cells and tumor tissue. The aqueous phase was 0.1% formic acid solution, and the organic phase was acetonitrile. Cells were washed with ice‐cold PBS, repeatedly freeze‐thawed with pure water, and lyzed by sonication to prepare cell samples for amino acid quantification. ^13^C‐L‐glutamine was diluted with assay buffer solution (137 mm NaCl, 5.1 mm KCl, 0.77 mm KH_2_PO_4_, 0.71 mm MgSO_4_, 1.1 mm CaCl_2_, 10 mm D‐glucose, and 10 mm HEPES, PH = 6.5) to a final concentration of 10 µm. The cells were washed with buffer solution (hot) for three times, incubated with ^13^C‐L‐glutamine at 37 °C for 15 min, then washed with buffer solution (cold) for three times, repeatedly freeze‐thawed with pure water, and lyzed by sonication to prepare cell samples for ^13^C‐L‐glutamine uptake assays. The collected tumor cells were weighed to the same mass and homogenized in the medium of methanol: water (80:20) to extract the metabolites, and stored at −80 °C for more than 2 h to precipitate the proteins, followed by centrifugation at 4 °C at 12 000 rpm for 10 min to isolate the supernatant. After drying, 50% acetonitrile was added to re‐dissolve for metabolite content assays. The samples were extracted with acetonitrile, centrifuged, injected with Shim‐pack Scepter Diol‐HILIC‐120 (2.1 × 100 mm, 1.9µm, SHIMADZU), and monitored in positive ion mode on a triple quadrupole 8045 mass spectrometer. Protein concentrations were determined by processing parallel groups to standardize amino acids content.

### Flow Cytometry Analysis (FACS)

For the analysis of apoptosis, cells were seeded in six‐well plates and incubated for 12–24 h at 5% CO_2_, 37 °C. The compounds were prepared into a master mix of 10^4^ µm with DMSO, and then, diluted to an appropriate concentration with medium containing 10% FBS. This allowed the drugs to stimulate the cells for 24–48 h. Cells and cell supernatants were collected and washed by centrifugation at 1800 rpm, 4 °C for 5 min. Then, 100 µL of 1× Binding Buffer was added for a single‐cell suspension. After that, 5µL of Annexin V‐FITC and 5 µL of PI Staining Solution were added to the sample tubes, incubated at room temperature away from light for 10 min, and after incubation, 400 µL 1× Binding Buffer was added. The stained samples were detected by flow cytometry (FACSCelesta) within 1 h.

For the analysis of immune cells, MC38‐bearing mice were administered subcutaneously for 6 days when the tumor size reached ≈400 mm^3^. The tumors were collected within 1 h after the final dose. Isolated tumor cells and spleen were washed with PBS; 4 mL of digestive solution was added to the tumor samples and digested at 37 °C, 100 rpm for 25 min. After digestion, the tumor cells were ground and washed with PBS and centrifuged at 2000 rpm for 5 min; the spleen samples were ground and added to erythrocyte lysate, washed with 4 mL PBS, and centrifuged at 2000 rpm for 5 min. After that, the tumor samples were resuspended with 5 mL 40% Percoll and slowly added to 70% Percoll, followed by centrifugation at 20 °C, 2000 rpm for 20 min to obtain single nucleated cells. Then, tumor and spleen mixed samples were blocked with Mouse FcR blocking reagent, and the antibodies were added to stain samples at 4 °C for 30 min in the dark. The samples to be membrane‐broken were washed with PBS and centrifuged at 2000 rpm for 5 min, and the cells were resuspended with fix/perm membrane‐broken solution, and the membrane was broken at 4 °C for 45 min. After membrane‐braking, the cells were washed and resuspended with perm/wash membrane‐broken solution, and then, the corresponding antibodies were added and the cells were incubated at 4 °C for 30 min in the dark. After incubation, the cells were washed by adding perm/wash, resuspended with PBS. All of the stained samples were detected by flow cytometry (FACSCelesta). The antibodies of flow cytometry analysis used are listed in Table S9, Supporting Information.

### Enzyme Linked‐Immunosorbent Assay (ELISA)

The antigenicity of all samples was evaluated using Polyclonal Antibodies Quantification Kits purchased from DAKEWE Biotech Co., Ltd., China. To each well, 100 µL of standard solutions and samples were added and incubated with 50 µL Biotinylated Antibody at 37 °C for 90 min. The wells were then emptied and washed four times with 300 µL 1× Washing Buffer, followed by 100 µL of the Streptavidin‐HRP solution, incubation at 37 °C for 30 min in the dark, and washing with 1× Washing Buffer four times. Then, 100 µL of TMB was added and incubated at 37 °C for 20 min in the dark. The reaction was stopped by adding 100 µL of stop solution into each well, and the absorbance was measured simultaneously at measurement wavelength 450 nm and reference wavelength 630 nm by microplate reader (Molecular Devices).

### Organoids Establishment and Drug Sensitivity Test

Patient‐derived fresh tumor tissues were further cultured into PDO models. H1975OR and HCC827OR cells were collected, diluted with Matrigel (Corning), and seeded with gel drops (10 µL, 3000 cells) for 3D culture. After the cells could proliferate normally and stably passaged, the 3D model was successfully constructed. The organoids (PDOs and 3D models) were photographed and recorded. Organoids were dissociated into homogeneous cell masses, resuspended, mixed with Matrigel at a ratio of 2:3 and seeded into 384‐well plates (10 µL, 2000 cells per well). After gelation, medium was added to culture cells for 48 h, then treated with drugs for 96 h. Cell viability was measured using the CellTiter‐Glo Viability Assay Kit (Promega) according to the manufacturer's instructions. The basic information of organoids is listed in Table S4, Supporting Information.

### In Vivo Experiments

For single‐dose acute toxicity test, the C57BL/6J (6–7 weeks) mice were divided into four groups containing Vehicle, DON (6 mg kg^−1^, s.c.), 10e (6 mg kg^−1^ DON equiv., s.c.), and 10e (12 mg kg^−1^ DON equiv., s.c.). All the agents were administered by subcutaneous injection for one time, and then the survival situation was observed.

For LLC subcutaneous tumor model, ≈5 × 10^5^ LLC cells suspended in PBS (100 µL) were injected into the right axilla of C57BL/6J mice (6–7 weeks). After the tumors grew to ≈100 mm^3^, all the mice were randomized into four groups (six mice for each group) containing Vehicle (normal saline, s.c.), DON (0.5 mg kg^−1^, s.c.), DRP104 (0.5 mg kg^−1^ DON equiv., s.c.), and 10e (0.5 mg kg^−1^ DON equiv., s.c.), and all the agents were continuously administered for 6 days on 8 days off by subcutaneous injection.

For MC38 subcutaneous tumor model, ≈3 ×10^5^ MC38 cells suspended in PBS (100 µL) were injected into the right axilla of C57BL/6J mice (6–7 weeks). After the tumors grew to ≈100 mm3, all the mice were randomized into two groups (five mice for each group) containing Vehicle (normal saline, s.c.) and 10e (0.5 mg kg^−1^ DON equiv., s.c.), and all agents were continuously administered for 6 days on 8 days off by subcutaneous injection.

For H1975OR subcutaneous tumor model, ≈1 × 10^7^ H1975OR cells suspended in PBS (100 µL) were injected into the right axilla of BALB/c‐Nude mice (5–6 weeks). After the tumors grew to ≈100 mm^3^, all the mice were randomized into four groups (ten mice for each group) containing Vehicle (normal saline, s.c.), LL202 (30 mg kg^−1^, s.c.), V9302 (30 mg kg^−1^, s.c.) and LL202+V9302 (30 mg kg^−1^, s.c.), and all agents were continuously administered for 20 days by subcutaneous injection.

For HCC827OR subcutaneous tumor model, ≈5 × 10^6^ HCC827OR cells suspended in PBS (100 µL) was injected into the right axilla of BALB/c‐Nude mice (5–6 weeks). After the tumors grew to ≈100 mm^3^, all the mice were randomized into three groups (eight mice for each group) containing Vehicle (normal saline, s.c.), DRP104 (0.4 mg kg^−1^ DON equiv., s.c.) and 10e (0.4 mg kg^−1^ DON equiv., s.c.), and all agents were continuously administered for 5 days on 4 days off for first cycle, and continuously administered for 5 days on 2 days off for the next three cycles by subcutaneous injection.

DRP104 and 10e were dissolved in 5% DMSO and 0.9% saline solution. DON was diluted to the appropriate concentration with 0.9% saline solution. The tumor growth and body weights were measured and recorded every day. As the endpoint of treatment, mice were sacrificed for collecting tumor and gastrointestinal tissue, and washed with PBS. The gastrointestinal tissue was taken and fixed by adding 4% paraformaldehyde, and tumors were weighed and photographed after water removal. Then, samples were rapidly frozen in liquid nitrogen and stored in −80 °C for histology and metabolomics. The relevant formula was calculated as:

(1) $$\mathrm{Tumor}\;\mathrm{volume}\;\left(\mathrm{TV},\;mm^{3}\right)=\mathrm{length}\;\left(\mathrm{mm}\right)\times \frac{\mathrm{width}\;{\left(\mathrm{mm}\right)}^{2}}{2}$$

(2) $$\begin{array}{ccc} & & \mathrm{Tumor}\;\mathrm{growth}\;\mathrm{inhibition}\;\left(\mathrm{TGI},\;\%\right)\\ & & =\left(1-\frac{TV_{\mathrm{Treatment}\_\mathrm{DayN}}\;-\;TV_{\mathrm{Treatment}\_\mathrm{Day}0}}{TV_{\mathrm{Vehicle}\_\mathrm{DayN}}\;-\;TV_{\mathrm{Vehicle}\_\mathrm{Day}0}}\right)\;\times 100\% \end{array}$$

TV_Treatment_DayN_: tumor volume on the day of administration for the treatment group, TV_Treatment_Day0_: tumor volume at the beginning of the experiment for treatment group, TV_Vehicle_DayN_: tumor volume on the day of the experiment for vehicle group, and TV_Vehicle_Day0_: tumor volume at the beginning of the experiment for vehicle group.

All animal experiments were performed according to protocols approved by the Animal Ethics Committee of China Pharmaceutical University (Approval No.2023‐10‐018, 2024‐05‐024, 2024‐07‐016).

### Histology

For Immunohistochemistry (IHC), the heart, liver, spleen, lung, kidney, and tumor of mice were removed quickly and fixed with 4% paraformaldehyde for 12 h. The tissue sections were stained using UltraSensitiveTM SP (Mouse/Rabbit) IHC Kit (Maxim) and anti‐γH2AX antibody (Santa Cruz Biotechnology) according to manufacturer's instructions. Tissue sections were stained using the TUNEL Apoptosis Detection Kit (Keygen Biotech) according to the manufacturer's instructions. Histological sections were scanned using the Olympus inverted phase contrast microscope (Olympus Corporation) at 400× field of view.

For hematoxylin‐eosin staining (H&E), animal models were executed as mentioned above and dissected surgically for the evaluation of possible pathological changes. The collection of gastrointestinal tissue was fixed in 10% buffered formalin, dehydrated in ethanol, embedded in paraffin, and then stained with hematoxylin and eosin. The pathological changes were captured with a Nikon 80i optical microscope.

### Statistical Analysis

The results are presented as the mean ± SD. Differences between groups were analyzed for statistical significance using *t*‐test analysis in GraphPad Prism (Version 8.3.0, GraphPad Software Inc., LLC). Statistical significance was accepted with *P* < 0.05, *: *P* < 0.05, **: *P* < 0.01, ^#^: *P* < 0.05, ^##^: *P* < 0.01, and ns: no significant difference. The data analysis was conducted by using GraphPad Prism and SPSS (Version 25, IBM SPSS Software Statistics Inc., LLC).

## Conflict of Interest

The authors declare no conflict of interest.

## Author Contributions

J.H. and J.B. conceived the project. J.H., X.Z., and H.Z. carried out the biological experiments and interpreted data. J.H. and Y.L. synthesized, purified, and characterized compounds. H.H., Z.Q., H.W., and X.X. were involved in directing the experiment. Z.L. and D.H. oversaw and provided insights and materials. J.H. and J.B. wrote the manuscript with input from all authors. Z.L., D.H., and J.B. provided supervision.

## Supporting information



Supporting Information

## Data Availability

The data that support the findings of this study are available from the corresponding author upon reasonable request.

## References

[1] R. L. Siegel , A. N. Giaquinto , A. Jemal , CA ‐ Cancer J. Clin. 2024, 74, 12. 38230766 10.3322/caac.21820

[2] K. Fu , F. Xie , F. Wang , L. Fu , J. Hematol. Oncol. 2022, 15, 173. 36482474 10.1186/s13045-022-01391-4 PMC9733018

[3] J. C. Soria , Y. Ohe , J. Vansteenkiste , T. Reungwetwattana , B. Chewaskulyong , K. H. Lee , A. Dechaphunkul , F. Imamura , N. Nogami , T. Kurata , I. Okamoto , C. Zhou , B. C. Cho , Y. Cheng , E. K. Cho , P. J. Voon , D. Planchard , W. C. Su , J. E. Gray , S. M. Lee , R. Hodge , M. Marotti , Y. Rukazenkov , S. S. Ramalingam , F. Investigators , N. Engl. J. Med. 2018, 378, 113.29151359

[4] J. Remon , C. E. Steuer , S. S. Ramalingam , E. Felip , Ann. Oncol. 2018, 29, i20. 29462255 10.1093/annonc/mdx704

[5] S. S. Ramalingam , J. C. Yang , C. K. Lee , T. Kurata , D. W. Kim , T. John , N. Nogami , Y. Ohe , H. Mann , Y. Rukazenkov , S. Ghiorghiu , D. Stetson , A. Markovets , J. C. Barrett , K. S. Thress , P. A. Janne , J. Clin. Oncol. 2018, 36, 841. 28841389 10.1200/JCO.2017.74.7576

[6] S. S. Ramalingam , J. Vansteenkiste , D. Planchard , B. C. Cho , J. E. Gray , Y. Ohe , C. Zhou , T. Reungwetwattana , Y. Cheng , B. Chewaskulyong , R. Shah , M. Cobo , K. H. Lee , P. Cheema , M. Tiseo , T. John , M. C. Lin , F. Imamura , T. Kurata , A. Todd , R. Hodge , M. Saggese , Y. Rukazenkov , J. C. Soria , F. Investigators , N. Engl. J. Med. 2020, 382, 41. 31751012 10.1056/NEJMoa1913662

[7] D. Westover , J. Zugazagoitia , B. C. Cho , C. M. Lovly , L. Paz‐Ares , Ann. Oncol. 2018, 29, i10. 29462254 10.1093/annonc/mdx703 PMC6454547

[8] B. C. Cho , D. W. Kim , A. I. Spira , J. E. Gomez , E. B. Haura , S. W. Kim , R. E. Sanborn , E. K. Cho , K. H. Lee , A. Minchom , J. S. Lee , J. Y. Han , M. Nagasaka , J. K. Sabari , S. I. Ou , P. Lorenzini , J. M. Bauml , J. C. Curtin , A. Roshak , G. Gao , J. Xie , M. Thayu , R. E. Knoblauch , K. Park , Nat. Med. 2023, 29, 2577. 37710001 10.1038/s41591-023-02554-7 PMC10579096

[9] J. K. Rotow , J. K. Lee , R. W. Madison , G. R. Oxnard , P. A. Janne , A. B. Schrock , J. Thorac. Oncol. 2024, 19, 227. 37806383 10.1016/j.jtho.2023.09.1453

[10] S. Wu , T. Fukumoto , J. Lin , T. Nacarelli , Y. Wang , D. Ong , H. Liu , N. Fatkhutdinov , J. A. Zundell , S. Karakashev , W. Zhou , L. E. Schwartz , H. Y. Tang , R. Drapkin , Q. Liu , D. G. Huntsman , A. V. Kossenkov , D. W. Speicher , Z. T. Schug , C. Van Dang , R. Zhang , Nat. Cancer 2021, 2, 189. 34085048 10.1038/s43018-020-00160-x PMC8168620

[11] P. Lee , D. Malik , N. Perkons , P. Huangyang , S. Khare , S. Rhoades , Y. Y. Gong , M. Burrows , J. M. Finan , I. Nissim , T. P. F. Gade , A. M. Weljie , M. C. Simon , Nat. Commun. 2020, 11, 498. 31980651 10.1038/s41467-020-14374-1 PMC6981153

[12] B. Jiang , J. Zhang , G. Zhao , M. Liu , J. Hu , F. Lin , J. Wang , W. Zhao , H. Ma , C. Zhang , C. Wu , L. Yao , Q. Liu , X. Chen , Y. Cao , Y. Zheng , C. Zhang , A. Han , D. Lin , Q. Li , Mol. Cell 2022, 82, 1821. 35381197 10.1016/j.molcel.2022.03.016

[13] X. Xu , J. Wang , M. Wang , X. Yuan , L. Li , C. Zhang , H. Huang , T. Jing , C. Wang , C. Tong , L. Zhou , Y. Meng , P. Xu , J. Kou , Z. Qiu , Z. Li , J. Bian , J. Med. Chem. 2021, 64, 4588. 33792311 10.1021/acs.jmedchem.0c02044

[14] Y. Zhao , X. Feng , Y. Chen , J. E. Selfridge , S. Gorityala , Z. Du , J. M. Wang , Y. Hao , G. Cioffi , R. A. Conlon , J. S. Barnholtz‐Sloan , J. Saltzman , S. S. Krishnamurthi , S. Vinayak , M. Veigl , Y. Xu , D. L. Bajor , S. D. Markowitz , N. J. Meropol , J. R. Eads , Z. Wang , Cancer Res. 2020, 80, 4815. 32907836 10.1158/0008-5472.CAN-20-0600 PMC7642187

[15] A. Rahman , F. P. Smith , P. T. Luc , P. V. Woolley , Invest. New Drugs 1985, 3, 369. 4086244 10.1007/BF00170760

[16] R. Pillai , T. Papagiannakopoulous , Cancer Res. 2024, 84, 349. 38117482 10.1158/0008-5472.CAN-23-3954

[17] R. Rais , K. M. Lemberg , L. Tenora , M. L. Arwood , A. Pal , J. Alt , Y. Wu , J. Lam , J. M. H. Aguilar , L. Zhao , D. E. Peters , C. Tallon , R. Pandey , A. G. Thomas , R. P. Dash , T. Seiwert , P. Majer , R. D. Leone , J. D. Powell , B. S. Slusher , Sci. Adv. 2022, 8, eabq5925. 36383674 10.1126/sciadv.abq5925 PMC9668306

[18] R. Pillai , S. E. LeBoeuf , Y. Hao , C. New , J. L. E. Blum , A. Rashidfarrokhi , S. M. Huang , C. Bahamon , W. L. Wu , B. Karadal‐Ferrena , A. Herrera , E. Ivanova , M. Cross , J. P. Bossowski , H. Ding , M. Hayashi , S. Rajalingam , T. Karakousi , V. I. Sayin , K. M. Khanna , K. K. Wong , R. Wild , A. Tsirigos , J. T. Poirier , C. M. Rudin , S. M. Davidson , S. B. Koralov , T. Papagiannakopoulos , Sci. Adv. 2024, 10, eadm9859. 38536921 10.1126/sciadv.adm9859 PMC10971495

[19] J. Encarnacion‐Rosado , A. S. W. Sohn , D. E. Biancur , E. Y. Lin , V. Osorio‐Vasquez , T. Rodrick , D. Gonzalez‐Baerga , E. Zhao , Y. Yokoyama , D. M. Simeone , D. R. Jones , S. J. Parker , R. Wild , A. C. Kimmelman , Nat. Cancer 2024, 5, 85. 37814010 10.1038/s43018-023-00647-3 PMC10824664

[20] M. V. Recouvreux , S. F. Grenier , Y. Zhang , E. Esparza , G. Lambies , C. M. Galapate , S. Maganti , K. Duong‐Polk , D. Bhullar , R. Naeem , D. A. Scott , A. M. Lowy , H. Tiriac , C. Commisso , Nat. Cancer 2024, 5, 100. 37814011 10.1038/s43018-023-00649-1 PMC10956382

[21] Y. Mitsuishi , K. Taguchi , Y. Kawatani , T. Shibata , T. Nukiwa , H. Aburatani , M. Yamamoto , H. Motohashi , Cancer Cell 2012, 22, 66. 22789539 10.1016/j.ccr.2012.05.016

[22] R. Liu , Y. Li , L. Tian , H. Shi , J. Wang , Y. Liang , B. Sun , S. Wang , M. Zhou , L. Wu , J. Nie , B. Lin , S. Tang , Y. Zhang , G. Wang , C. Zhang , J. Han , B. Xu , L. Liu , K. Gong , T. Zheng , Cancer Lett. 2019, 443, 34. 30503555 10.1016/j.canlet.2018.11.030

[23] A. Mendez‐Lucas , W. Lin , P. C. Driscoll , N. Legrave , L. Novellasdemunt , C. Xie , M. Charles , Z. Wilson , N. P. Jones , S. Rayport , M. Rodriguez‐Justo , V. Li , J. I. MacRae , N. Hay , X. Chen , M. Yuneva , Nat. Metab. 2020, 2, 335. 32694609 10.1038/s42255-020-0195-8 PMC7436715

[24] R. D. Leone , L. Zhao , J. M. Englert , I. M. Sun , M. H. Oh , I. H. Sun , M. L. Arwood , I. A. Bettencourt , C. H. Patel , J. Wen , A. Tam , R. L. Blosser , E. Prchalova , J. Alt , R. Rais , B. S. Slusher , J. D. Powell , Science 2019, 366, 1013. 31699883 10.1126/science.aav2588 PMC7023461

[25] S. Son , M. Won , O. Green , N. Hananya , A. Sharma , Y. Jeon , J. H. Kwak , J. L. Sessler , D. Shabat , J. S. Kim , Angew. Chem., Int. Ed. 2019, 58, 1739. 10.1002/anie.201813032 30561862

[26] S. Aujla , C. Aloe , A. Vannitamby , S. Hendry , K. Rangamuwa , H. Wang , R. Vlahos , S. Selemidis , T. Leong , D. Steinfort , S. Bozinovski , J. Thorac. Oncol. 2022, 17, 675. 35124252 10.1016/j.jtho.2022.01.013

[27] L. Z. Zhang , J. G. Yang , G. L. Chen , Q. H. Xie , Q. Y. Fu , H. F. Xia , Y. C. Li , J. Huang , Y. Li , M. Wu , H. M. Liu , F. B. Wang , K. Z. Yi , H. G. Jiang , F. X. Zhou , W. Wang , Z. L. Yu , W. Zhang , Y. H. Zhong , Z. Bian , H. Y. Yang , B. Liu , G. Chen , Nat. Commun. 2024, 15, 3884. 38719909 10.1038/s41467-024-48200-9 PMC11079016

[28] L. Liu , D. Ge , L. Ma , J. Mei , S. Liu , Q. Zhang , F. Ren , H. Liao , Q. Pu , T. Wang , Z. You , J. Thorac. Oncol. 2012, 7, 1091. 22534817 10.1097/JTO.0b013e3182542752 PMC3378786

[29] D. Chowell , L. G. T. Morris , C. M. Grigg , J. K. Weber , R. M. Samstein , V. Makarov , F. Kuo , S. M. Kendall , D. Requena , N. Riaz , B. Greenbaum , J. Carroll , E. Garon , D. M. Hyman , A. Zehir , D. Solit , M. Berger , R. Zhou , N. A. Rizvi , T. A. Chan , Science 2018, 359, 582. 29217585 10.1126/science.aao4572 PMC6057471

[30] T. Bertero , W. M. Oldham , E. M. Grasset , I. Bourget , E. Boulter , S. Pisano , P. Hofman , F. Bellvert , G. Meneguzzi , D. V. Bulavin , S. Estrach , C. C. Feral , S. Y. Chan , A. Bozec , C. Gaggioli , Cell Metab. 2019, 29, 124. 30293773 10.1016/j.cmet.2018.09.012 PMC6432652

[31] A. A. Osman , E. Arslan , M. Bartels , C. Michikawa , A. Lindemann , K. Tomczak , W. Yu , V. Sandulache , W. Ma , L. Shen , J. Wang , A. K. Singh , M. J. Frederick , N. D. Spencer , J. Kovacs , T. Heffernan , W. F. Symmans , K. Rai , J. N. Myers , Clin. Cancer Res. 2023, 29, 1344. 36689560 10.1158/1078-0432.CCR-22-2747 PMC10068451

[32] E. B. Blatt , R. J. DeBerardinis , Sci. Adv. 2024, 10, eado7808. 38536918 10.1126/sciadv.ado7808 PMC10971402

[33] X. Xu , X. Chang , J. Huang , D. Zhang , M. Wang , T. Jing , Y. Zhuang , J. Kou , Z. Qiu , J. Wang , Z. Li , J. Bian , Eur. J. Med. Chem. 2022, 236, 114337. 35428013 10.1016/j.ejmech.2022.114337

[34] K. E. de Visser , J. A. Joyce , Cancer Cell 2023, 41, 374. 36917948 10.1016/j.ccell.2023.02.016

[35] V. E. Baracos , L. Martin , M. Korc , D. C. Guttridge , K. C. H. Fearon , Nat. Rev. Dis. Primers 2018, 4, 17105. 29345251 10.1038/nrdp.2017.105

[36] A. Keegan , B. Ricciuti , P. Garden , L. Cohen , R. Nishihara , A. Adeni , C. Paweletz , J. Supplee , P. A. Janne , M. Severgnini , M. M. Awad , D. R. Walt , J. Immunother. Cancer 2020, 8, 000678. 10.1136/jitc-2020-000678 PMC7537334 33020238

[37] A. R. Naqash , J. D. McCallen , E. Mi , S. Iivanainen , M. A. Marie , D. Gramenitskaya , J. Clark , J. P. Koivunen , S. Macherla , S. Jonnalagadda , S. Polsani , R. A. Jiwani , M. Hafiz , M. Muzaffar , L. Brunetti , C. R. G. Stroud , P. R. Walker , K. Wang , Y. Chung , E. Ruppin , S. H. Lee , L. V. Yang , D. J. Pinato , J. S. Lee , A. Cortellini , J. Immunother. Cancer 2023, 11. 10.1136/jitc-2023-007310 PMC10603340 37852738

[38] E. N. Arner , J. C. Rathmell , Cancer Cell 2023, 41, 421. 36801000 10.1016/j.ccell.2023.01.009 PMC10023409

[39] J. Liu , Y. Bai , Y. Li , X. Li , K. Luo , EBioMedicine 2024, 107, 105301. 39178747 10.1016/j.ebiom.2024.105301 PMC11388279

