# Supplementary material for: Addressing Clinical Limitations of Glutaminase Inhibitors: Novel Strategies for Osimertinib‐Resistant Lung Cancer by Exploiting Glutamine Metabolic Dependency
Source: Adv Sci (Weinh). 2024 Dec 16;12(6):2411479. doi: 10.1002/advs.202411479 (PMC11809341; doi:10.1002/advs.202411479)

Supporting Information

Addressing Clinical Limitations of Glutaminase Inhibitors: Novel Strategies for Osimertinib-Resistant Lung Cancer by Exploiting Glutamine Metabolic Dependency

*Jiali Huang, Xiankang Zhang, Hui Zhang, Yu Li, Huidan Huang, Zhiyu Li*, Zhixia Qiu, Hongxi Wu, Dechun Huang*, Xi Xu*, Jinlei Bian**

**Experimental Section**

1.Chemistry

1.1 General chemistry methods

Relevant solvents were obtained from Shanghai Lingfeng Chemical Reagents Co., Ltd or Sinopharm Chemical Reagent Co., Ltd. and used without further purification. The positive control JHU083 and DRP104 was purchased from GLPBIO. The experimental animals were purchased from Beijing Vital River Laboratory Animal Technology Co., Ltd. The high-performance liquid chromatography (HPLC) grade methanol, catalysts and other reagents from commercial sources were used as received. Thin-layer chromatography (TLC) was performed on 0.25 mm silica gel plates with a fluorescent indicator (GF254) and visualized under UV light. With tetramethylsilane (TMS) as an internal standard, the 1H and 13C NMR spectra were measured at 300 and 75 MHz, respectively. The samples were dissolved in deuterated solvent. The chemical shifts are given in δ-scale, the coupling constants J are given in Hz. HRMS spectra data was acquired on Agilent technologies 6520 Accurate-Mass Q-TOF LC/MS instruments. Each compound was purified via silica gel (60 Å, 100-300 mesh) column chromatography. The purity of compounds subjected to biological tests was over 95%, which were determined by HPLC (Shimadzu Labsolutions) equipped with the Agilent C18 column (4.6 mm × 150 mm, 5 μm) using gradient elution (mobile phase: A phase = H2O, B phase = MeOH) at a flow rate of 1 mL/min. (Gradient, 0.01 to 2 min, A/B, keep 90/10, 2 to 12 min, A/B from 90/10 to 5/95, 12 to 20 min, A/B, keep 5/95, 20 to 21 min, A/B from 5/95 to 90/10, 21 to 25 min, A/B, keep 90/10.)

1.2 Synthetic procedures

*1.2.1 Ethyl 5-oxopyrrolidine-2-carboxylate (****2a****).*

**1a** (2.00 g, 15.5 mmol) and TsOH (0.27 g, 1.55 mmol) were dissolved in 40 mL of ethanol and the reaction mixture was stirred at reflux temperature for 18 h. After the reaction was finished, the solvent was evaporated and the residue was redissolved in DCM. The organic phase was washed sequentially with saturated NaHCO_3_ and H_2_O three times. The combined water phase was collected and extracted with DCM again. The combined organic layers were dried over anhydrous Na_2_SO_4_, filtered, and evaporated to afford **2a** as a white solid at 1.92 g, yield 83.3%. HRMS (ESI^+^): m/z (M + H)^+^ calculated for C_7_H_12_NO_3_^+^, 157.0739; found, 157.0732.

*1.2.2 Isopropyl 5-oxopyrrolidine-2-carboxylate (****2b****).*

**1a** (10.00 g, 77.5 mmol) and TsOH (1.35 g, 7.55 mmol) were dissolved in 200 mL of isopropanol and the reaction mixture was stirred at reflux temperature for 18 h. Then the reaction solvent was evaporated and the residue was added into 300 mL of ethyl acetate. The organic phase was washed sequentially with saturated NaHCO_3_ and H_2_O three times. The combined water phase was collected and extracted with ethyl acetate again. The combined organic layers were dried over anhydrous Na_2_SO_4_, filtered, and concentrated in vacuo. The residue was recrystallized by hexane to give **2b** as a white solid at 11.03 g, yield 85.0%. ^1^H NMR (300 MHz, CDCl_3_): *δ* 6.54 (s, 1H), 5.12−5.06 (m, 1H), 4.26−4.22 (m, 1H), 2.51−2.37 (m, 3H), 2.28−2.23 (m, 1H), 1.30 (d, 6H, *J* = 6.0 Hz) ppm. HRMS (ESI^+^): m/z (M + H)^+^ calculated for C_8_H_14_NO_3_^+^, 172.0968; found, 172.1002.

*1.2.3 Cyclohexyl-5-oxopyrrolidine-2-carboxylate (****2c****).*

**1a** (1.00 g, 7.74 mmol) and cyclohexanol (0.93 g, 9.29 mmol) were dissolved in 20 mL of DCM and the reaction mixture was stirred at room temperature (24 °C) for 10 min. Then EDCI (1.78 g, 9.29 mmol) and DMAP (0.10 g, 0.77 mmol) were added and the mixture was stirred at room temperature for 3 h. The solvent was concentrated and the residue was poured into 100 mL of saturated NaHCO_3_ prior to being extracted with ethyl acetate three times. The combined organic layers were washed with water two times, dried over anhydrous Na_2_SO_4_, filtered, and evaporated to afford **2c** as a white solid at 1.34 g, yield 82.0%. The crude product was directly used in the next step without further purification. ^1^H NMR (300 MHz, CDCl_3_): *δ* 6.28 (s, 1H), 4.91−4.85 (m, 1H), 4.30−4.25 (m, 1H), 2.57−2.39 (m, 3H), 2.33−2.23 (m, 1H), 1.91−1.89 (m, 3H), 1.79−1.77 (m, 2H), 1.77−1.31 (m, 6H) ppm. HRMS (ESI^+^) m/z (M + H)^+^ calculated for C_11_H_18_NO_3_^+^, 212.1281; found, 212.1263.

*1.2.4 3-Phenylpropyl 5-oxopyrrolidine-2-carboxylate (****2d****).*

As described in the preparation of **2c**, the intermediate **2d** was prepared by using phenylpropanol instead of cyclohexanol, and the yield was 73.0%. The crude product was directly used in the next step without further purification. HRMS (ESI^+^) m/z (M + H) ^+^ calculated for C_14_H_18_NO_3_^+^, 248.1281; found, 248.1273.

*1.2.5 1-(9H-Fluoren-9-ylmethyl)-2-ethyl-5-oxopyrrolidine-1,2-dicarboxylate (****3a****).*

**2a** (3.00 g, 14.21 mmol) was dissolved in 30 mL of anhydrous THF. After cooling to −78 °C, LiHMDS (1 M solution in THF, 13.50 mmol) was added slowly and the reaction was stirred for 20 min. Then the solution of Fmoc-Cl (4.77 g, 18.47 mmol) in dry THF (40 mL) was added dropwise into the mixture. After the reaction was completed, the pre-cooling saturated NH_4_Cl was added and the mixture was extracted with ethyl acetate. The combined organic layers were dried over anhydrous Na_2_SO_4_, filtered, and evaporated. The crude product was purified by silica gel chromatography to afford **3a** as a white solid at 5.83 g, yield 82.3%. ^1^H NMR (300 MHz, CDCl_3_): *δ* 7.85−7.76 (m, 4H), 7.50−7.40 (m, 4H), 4.74−4.70 (m, 2H), 4.63−4.46 (m, 2H), 4.39−4.23 (m, 2H), 2.85−2.64 (m, 2H), 2.62−2.14 (m, 2H), 1.33 (t, 3H, *J* = 12.0 Hz) ppm. HRMS (ESI^+^): m/z (M + Na)^+^ calculated for C_22_H_21_NO_5_Na^+^, 402.1312; found, 402.1344.

*1.2.6 1-((9H-Fluoren-9-yl)-methyl)-2-isopropyl)-5-oxopyrrolidine-1,2-dicarboxylate* (***3b***).

As described in the preparation of **3a**, the intermediate **3b** was prepared by using **2b** instead of **2a**, and the yield was 83.6%. ^1^H NMR (300 MHz, CDCl_3_): *δ* 7.82−7.75 (m, 4H), 7.48−7.37 (m, 4H), 5.16−5.08 (m, 1H), 4.71−4.67 (m, 1H), 4.71−4.57 (m, 1H), 4.57−4.43 (m, 2H), 4.37−4.32 (m, 1H), 2.79−2.56 (m, 2H), 2.51−2.40 (m, 1H), 2.19−2.10 (m, 1H), 1.30 (d, 3H, *J* = 6.0 Hz), 1.28 (d, 3H, *J* = 6.0 Hz) ppm. HRMS (ESI^+^): m/z (M + Na)^+^ calculated for C_23_H_23_NO_5_Na^+^, 416.1468; found, 416.1462.

*1.2.7 (1-((9H-fluoren-9-yl)-methyl)-2-cyclohexyl-5-oxopyrrolidine-1,2-dicarboxylate (****3c****).*

As described in the preparation of **3a**, the intermediate **3c** was prepared by using **2c** instead of **2a**, and the yield was 82.3%. HRMS (ESI^+^): m/z (M + Na)^+^ calculated for C_26_H_27_NO_5_Na^+^, 456.1781; found, 456.1772.

*1.2.8 (1-((9H-fluoren-9-yl)-methyl)-2-cyclohexyl-5-oxopyrrolidine-1,2-dicarboxylate (****3d****).*

As described in the preparation of **3a**, the intermediate **3d** was prepared by using **2d** instead of **2a**, and the yield was 78.5%. HRMS (ESI^+^): m/z (M + Na)^+^ calculated for C_29_H_27_NO_5_Na^+^, 492.1781; found, 492.1773.

*1.2.9 Ethyl-2-((((9H-fluoren-9-yl)-methoxy)-carbonyl)-amino)-6-diazo-5-oxohexanoate (****4a****).*

Trimethylsilyl diazomethane (2 M solution in ether, 3.2 mL, 6.34 mmol) was dissolved in 30 mL of anhydrous THF. After cooling to −110 °C, *n-*butyllithium (2.5 M solution in hexane, 2.6 mL, 6.49 mmol) was added slowly and the mixture was stirred for 3 min. Then **3a** (2.2 g, 5.28 mmol) dissolved in 10 mL of anhydrous THF was added into the mixture dropwise, controlling the temperature below −110 °C . After the reaction was completed, the pre-cooling saturated NH_4_Cl was added and the mixture was extracted with ethyl acetate (3×50 mL). The combined organic layers were dried over anhydrous Na_2_SO_4_, filtered, and concentrated. The crude product was purified by silica gel chromatography to afford **4a** as a white solid at 0.61 g, yield 25.0%. ^1^H NMR (300 MHz, CDCl_3_): *δ* 7.82−7.80 (m, 2H), 7.65−7.60 (m, 2H), 7.47−7.34 (m, 4H), 5.62−5.59 (m, 1H), 5.31 (s, 1H), 4.45−4.39 (m, 2H), 4.29−4.24 (m, 2H), 2.47 (s, 2H), 2.29−2.27 (m, 1H), 2.09−2.05 (m,1H), 1.33 (t, 3H, *J* = 12.0 Hz) ppm. HRMS (ESI^+^): m/z (M + Na)^+^ calculated for C_23_H_23_NO_5_Na^+^, 444.1530; found, 444.1536.

*1.2.10 Isopropyl-2-((((9H-fluoren-9-yl)-methoxy)-carbonyl)-amino)-6-diazo-5-oxohexano- ate (***4b***).*

As described in the preparation of **4a**, the intermediate **4b** was prepared by using **3b** instead of **3a**, and the yield was 20.0%. ^1^H NMR (300 MHz, DMSO-*d_6_*): *δ* 7.85−7.83 (m, 2H), 7.72−7.65 (m, 2H), 7.39−7.25 (m, 4H), 4.88−4.30 (m, 1H), 4.32−4.15 (m, 3H), 3.98−3.89 (m, 1H), 2.36 (s, 2H), 1.98−1.86 (m, 1H), 1.81−1.68 (m, 1H), 1.13 (d, 3H, *J* = 3.0 Hz), 1.11 (d, 3H, *J* =3.0 Hz) ppm. HRMS (ESI^+^): m/z (M + Na)^+^ calculated for C_24_H_25_NO_5_Na^+^, 458.1687; found, 458.1680.

*1.2.11 2-(((9H-fluoren-9-yl)-methoxy)-carbonyl)-amino)-6-diazo-5-oxohexanoate (****4c****).*

As described in the preparation of **4a**, the intermediate **4c** was prepared by using **3c** instead of **3a**, and the yield was 21.0%. ^1^H NMR (300 MHz, DMSO-*d_6_*): *δ* 7.97−7.95 (m, 2H), 7.83−7.76 (m, 2H), 7.51−7.46 (m, 2H), 7.42−7.37 (m, 2H), 4.74 (s, 1H), 4.39−4.29 (m, 3H), 4.13−4.01 (m, 1H), 2.91 (s, 1H), 2.47 (s, 1H), 2.10−2.01 (m, 1H), 1.91−1.69 (m, 5H), 1.46−1.21 (m, 7H) ppm. HRMS (ESI^+^): m/z (M + Na)^+^ calculated for C_27_H_29_N_3_O_5_Na^+^, 475.2107; found, 475.2111.

*1.2.12 3-Phenylpropyl-2-((((9H-fluoren-9-yl)-methoxy)-carbonyl)-amino)-6-diazo-5-oxohexanoate (****4d****).*

As described in the preparation of **4a**, the intermediate **4d** was prepared by using **3d** instead of **3a**, and the yield was 18.0%. HRMS (ESI^+^): m/z (M + Na)^+^ calculated for C_30_H_29_N_3_O_5_Na^+^, 534.1999; found. 534.1983.

*1.2.13 Ethyl-2-amino-6-diazo-5-oxohexanoate (****5a****).*

**4a** (1.00 g, 2.37 mmol) was dissolved in anhydrous DCM (10 mL) followed by the addition of piperidine (590 μL, 5.93 mmol) and the reaction mixture was stirred at room temperature for 4 h. The solvent was evaporated. The crude product was purified by silica gel chromatography to afford **5a** as a yellow oil at 0.31 g, yield 66.0%. HRMS (ESI^+^): m/z (M + H)^+^ calculated for C_8_H_14_N_3_O_3_^+^, 200.1030; found, 200.1025.

*1.2.14 Isopropyl-2-amino-6-diazo-5-oxohexanoate (****5b****).*

As described in the preparation of **5a**, the intermediate **5b** was prepared by using **4b** instead of **4a**, and the yield was 59.0%. HRMS (ESI^+^): m/z (M + H)^+^ calculated for C_9_H_16_N_3_O_3_^+^, 214.1186; found, 214.1182.

*1.2.15 Cyclohexyl-2-amino-6-diazo-5-oxohexanoate (****5c****).*

As described in the preparation of **5a**, the intermediate **5c** was prepared by using **4c** instead of **4a**, and the yield was 66.0%. HRMS (ESI^+^): m/z (M + H)^+^ calculated for C_13_H_20_N_3_O_3_^+^, 254.1499; found, 254.1481.

*1.2.16 3-Phenylpropyl-2-amino-6-diazo-5-oxohexanoate (****5d****).*

As described in the preparation of **5a**, the intermediate **5d** was prepared by using **4d** instead of **4a**, and the yield was 66.0%. HRMS (ESI^+^): m/z (M + H)^+^ calculated for C_13_H_20_N_3_O_3_^+^, 254.1499; found, 254.1481.

*1.2.17 6-Hydroxy-4,4,5,7,8-pentamethylchroman-2-one (****8a****).*

**6a** (2.00 g, 13.1 mmol) and **7** (1.5 g, 15 mmol) were dissolved in 20 mL of methanesulfonic acid and the reaction mixture was stirred at 85 °C for 3 h under nitrogen. After cooling to room temperature (25 °C), the mixture was poured into 100 mL of ice water. The mixture was extracted with ethyl acetate (3×100 mL) and the combined organic layers were washed with saturated NaHCO_3_ (3×50 mL) and H_2_O (3×50 mL), dried over anhydrous Na_2_SO_4_, filtered, and concentrated in vacuo to afford a yellow solid. The crude product was recrystallized from hexane and ethyl acetate (2:1, v/v) to give **8a** as a white solid at 2.55 g, yield 83.0%. ^1^H NMR (300 MHz, CDCl_3_): *δ* 4.71 (s, 1H), 2.60 (s, 2H), 2.41 (s, 3H), 2.27 (s, 3H), 2.23 (s, 3H), 1.50 (s, 6H) ppm. HRMS (ESI^+^): m/z (M + H)^+^ calculated for C_14_H_19_O_3_^+^, 235.1329; found, 235.1317.

*1.2.18 6-Hydroxy-4,4,7,8-tetramethylchroman-2-one (****8b).***

As described in the preparation of **8a**, the intermediate **8b** was prepared by using **6b** instead of **6a**, and the yield was 72.0%. HRMS (ESI^+^): m/z (M + H)^+^ calculated for C_13_H_17_O_3_^+^, 221.1172; found, 221.1165.

*1.2.19 6-Hydroxy-4,4,5,7-tetramethylchroman-2-one (****8c****).*

As described in the preparation of **8a**, the intermediate **8c** was prepared by using **6c** instead of **6a**, and the yield was 58.0%. ^1^H NMR (300 MHz, DMSO-*d_6_*): *δ* 8.13 (s, 1H), 6.66 (s, 1H), 2.60 (s, 2H), 2.28 (s, 3H), 2.13 (s, 3H), 1.34 (s, 6H) ppm. HRMS (ESI^+^): m/z (M + H)^+^ calculated for C_13_H_17_O_3_^+^, 221.1172; found, 221.1162.

*1.2.20 3-Methyl-3-(2,4,5-trimethyl-3,6-dioxocyclohexa-1,4-dien-1-yl)-butanoic acid (****9a****).*

**8a** (2.5 g, 10.9 mmol) was dissolved in 30 mL of acetonitrile and 5 mL of H_2_O followed by the addition of *N*-bromosuccinimide (2.0 g, 11.5 mmol). The reaction mixture was stirred at room temperature for 1 h. The organic solvent was concentrated in vacuo and the residue was extracted with DCM (3 × 30 mL). The combined organic layers were dried over anhydrous Na_2_SO_4_, filtered, and concentrated in vacuo to give **9a** as a yellow solid at 1.54 g, yield 58.0%. ^1^H NMR (300 MHz, CDCl_3_): *δ* 3.07 (s, 2H), 2.19 (s, 3H), 2.00 (s, 3H), 1.97 (s, 3H), 1.48 (s, 6H) ppm. HRMS (ESI^+^): m/z (M + H)^+^ calculated for C_14_H_18_O_4_^+^, 250.1205; found, 250.1198.

*1.2.21 3-(4,5-Dimethyl-3,6-dioxocyclohexa-1,4-dien-1-yl)-3-methylbutanoic acid (****9b****).*

As described in the preparation of **9a**, the intermediate **9b** was prepared by using **8b** instead of **8a**, and the yield was 42.0%. HRMS (ESI^+^): m/z (M + H)^+^ calculated for C_13_H_16_O_4_^+^, 236.1049; found, 236.1033.

*1.2.22 3-(2,4-Dimethyl-3,6-dioxocyclohexa-1,4-dien-1-yl)-3-methylbutanoic acid (****9c****).*

As described in the preparation of **9a**, the intermediate **9c** was prepared by using **8c** instead of **8a**, and the yield was 46.0%. ^1^H NMR (300 MHz, CDCl_3_): *δ* 6.50 (s, 1H), 3.09 (s, 2H), 2.22 (s, 3H), 2.04 (s, 3H), 1.48 (s, 6H) ppm. HRMS (ESI^+^): m/z (M + H)^+^ calculated for C_13_H_16_O_4_^+^, 236.1049; found, 236.1037.

*1.2.23 3-(4-Bromo-2,5-dimethyl-3,6-dioxocyclohexa-1,4-dien-1-yl)-3-methylbutanoic acid (****9d****).*

As described in the preparation of **9c**, the intermediate **9d** was prepared by using liquid bromine instead of *N*-bromosuccinimide, and the yield was 23.0%. HRMS (ESI^+^): m/z (M + H)^+^ calculated for C_13_H_15_BrO_4_^+^, 314.0154; found, 314.0138.

*1.2.24 Ethyl-6-diazo-2-(3-methyl-3-(2,4,5-trimethyl-3,6-dioxocyclohexa-1,4-dien-1-yl)-butanamido)-5-oxohexanoate (****10a****).*

**9a** (1 g,3.99 mmol) was dissolved in 10 mL of DCM followed by the addition of HATU (1 g, 3.99 mmol) and DIPEA (1.83 g, 14.08 mmol) and the mixture was stirred at room temperature for 0.5 h. **5a** (0.80 g, 4.0 mmol) was added slowly and the reaction was stirred for another 4h. The solvent was concentrated and the residue was purified by silica gel chromatography to afford **10a** as a yellow solid at 1.07 g, yield 62.0%. mp 107−109 °C. ^1^H NMR (300 MHz, CDCl_3_): *δ* 6.28 (d, 1H, *J*=6.0 Hz), 5.30−5.26 (m, 1H), 4.47−4.40 (m, 1H), 4.18−4.11 (m, 2H), 2.83 (s, 2H), 2.36−2.33 (m, 2H), 2.11 (s, 3H), 1.95 (d, 6H, *J* = 6.0 Hz), 1.42 (s, 6H), 1.26 (t, 3H, *J* = 6.0 Hz) ppm. ^13^C NMR (75MHz, CDCl_3_): *δ* = 193.74, 191.11, 187.56, 171.90, 171.68, 153.05, 143.42, 137.99, 137.88, 125.23, 69.2, 66.2, 61.62, 51.59, 48.96, 38.28, 36.49, 28.92, 28.80, 14.11, 12.70, 12.13 ppm. HRMS (ESI^+^): m/z (M + H)^+^ calculated for C_22_H_30_N_3_O_6_^+^, 432.2129; found, 432.2177.

*1.2.25 Ethyl-6-diazo-2-(3-(4,5-dimethyl-3,6-dioxocyclohexa-1,4-dien-1-yl)-3-methylbutanamido)-5-oxohexanoate (****10b****).*

As described in the preparation of **10a**, compound **10b** was prepared by using **9b** instead of **9a**, and the yield was 51.0%. ^1^H NMR (300 MHz, CDCl_3_) *δ* 6.48 (s, 1H), 6.23 (d, 1H, *J* = 9.0 Hz), 5.24 (s, 1H), 4.44−4.37 (m, 1H), 4.17−4.10 (m, 2H), 2.86−2.73 (m, 2H), 2.36−2.31 (m, 2H), 2.13 (s, 1H), 2.04−1.98 (m, 7H), 1.61 (s, 6H), 1.32−1.24 (m, 3H) ppm. HRMS (ESI^+^): m/z (M + H)^+^ calculated for C_21_H_28_N_3_O_6_^+^, 418.1973; found, 418.1965.

*1.2.26 Ethyl-6-diazo-2-(3-(2,4-dimethyl-3,6-dioxocyclohexa-1,4-dien-1-yl)-3-methylbutanamido)-5-oxohexanoate (****10c****).*

**9d** (0.94 g, 3.99 mmol) was dissolved in 10 mL of DCM prior to the addition of EDCI (0.89 g, 4.69 mmol) and DMAP (0.05 g, 0.4 mmol) and the mixture was stirred at room temperature for 0.5 h. **5a** (0.80 g, 4.0 mmol) was added slowly and the reaction was stirred for another 4 h. The solvent was concentrated and the residue was purified by silica gel chromatography to afford **10d** as a brown solid at 0.64 g, yield 38.0%. mp 108−109 °C. ^1^H NMR (300 MHz, CDCl_3_): *δ* 6.31 (d, 1H, *J* = 9.0 Hz), 5.36 (s, 1H), 4.54−4.47 (m, 1H), 4.24−4.17 (m, 2H), 3.19−3.14 (m, 1H), 2.85 (s, 2H), 2.75−2.70 (m, 1H), 2.45−2.39 (m, 2H), 2.21−2.15 (m, 7H), 2.09−2.02 (m, 1H), 1.49 (d, 6H, *J* = 9.0 Hz), 1.33−1.27 (m, 3H) ppm. ^13^C NMR (75MHz, CDCl_3_): *δ* = 193.95, 184.70, 182.46, 171.72, 171.68, 153.86, 143.74, 138.09, 137.03, 136.52, 125.23, 61.66, 55.18, 51.66, 49.01, 38.82, 38.61, 36.58, 28.90, 28.44, 27. 10, 16.68, 14.44, 14.11 ppm. HRMS (ESI^+^): m/z (M + H)^+^ calculated for C_21_H_28_N_3_O_6_^+^, 418.1973; found, 418.1965.

*1.2.27 2-(3-(5-bromo-2,4-dimethyl-3,6-dioxocyclohexa-1,4-dien-1-yl)-3-methylbutanamido)-6-diazo-5-oxohexanoate (****10d****).*

**9c** (1.25 g, 3.99 mmol) was dissolved in 13 mL of DCM followed by the addition of EDCI (0.89 g, 4.69 mmol) and HOBT (0.62 g, 4.69 mmol) and the mixture was stirred at room temperature for 0.5 h. **5a** (0.80 g, 4.0 mmol) was added slowly and the reaction was stirred for another 4 h. Then the solvent was concentrated and the residue was purified by silica gel chromatography to afford **10c** as a brown solid at 0.66g, yield 33.0%. mp 115−117 °C. ^1^H NMR (300 MHz, CDCl_3_): *δ* 6.27 (d, 1H, *J* = 9.0 Hz), 5.31 (d, 1H, *J* = 6.0 Hz), 4.49−4.42 (m, 1H), 4.19−4.12 (m, 2H), 3.14−3.09 (m, 1H), 2.80 (s, 1H), 2.70−2.65 (m, 1H), 2.37−2.34 (m, 2H), 2.16 (s, 6H), 2.04−1.94 (m, 2H), 1.44 (d, 6H, *J* = 9.0 Hz), 1.27−1.25 (m, 3H) ppm. HRMS (ESI^+^): m/z (M + H)^+^ calculated for C_21_H_27_BrN_3_O_6_^+^, 496.1078; found. 496.1077.

*1.2.28 Isopropyl-6-diazo-2-(3-methyl-3-(2,4,5-trimethyl-3,6-dioxocyclohexa-1,4-dien-1-yl)-butanamido)-5-oxohexanoate (****10e****).*

As described in the preparation of **10a**, compound **10e** was prepared by using **5b** instead of **5a**, and the yield was 57%. mp 110−112 °C. ^1^H NMR (300 MHz, CDCl_3_): *δ* 6.23 (d, 1H, *J* = 6.0 Hz), 5.25 (s, 1H), 5.05−4.93 (m, 1H), 4.44−4.37 (m, 1H), 2.96−2.78 (m, 2H), 2.35−2.31 (m, 2H), 2.11 (s, 3H), 1.95 (d, 7H, *J* = 6.0 Hz), 1.43 (d, 6H, *J* = 3.0 Hz), 1.22 (d, 3H, *J* = 6.0 Hz), 1.22 (d, 3H, *J* = 6.0 Hz) ppm. HRMS (ESI^+^): m/z (M + Na)^+^ calculated for C_23_H_31_N_3_O_6_Na^+^, 468.2111; found, 468.2174.

*1.2.29 Isopropyl-6-diazo-2-(3-(2,4-dimethyl-3,6-dioxocyclohexa-1,4-dien-1-yl)-3-methylbutanamido)-5-oxohexanoate (****10f****).*

As described in the preparation of **10a**, compound **10f** was prepared by using **9c** and **5b** instead of **9a** and **5a**, respectively, and the yield was 53%. mp 111−112 °C. ^1^H NMR (300 MHz, DMSO-*d_6_*): *δ* 8.17 (d, 1H, *J* = 9.0 Hz), 6.55 (s, 1H), 6.01 (s, 1H), 4.80−4.76 (m, 1H), 4.04−3.96 (m, 1H), 2.66−2.62 (m, 2H), 2.29 (s, 2H), 1.98 (s, 4H), 1.85 (s, 4H), 1.29 (d, 6H, *J* = 6.0 Hz), 1.08 (d, 3H, *J* = 6.0 Hz), 1.08 (d, 3H, *J* = 6.0 Hz) ppm. ^13^C NMR (75MHz, CDCl_3_): *δ* 194.43, 189.96, 188.27, 171.81, 171.21, 152.05, 143.47, 139.16, 135.19, 128.18, 125.23, 69.44, 66.28, 51.66, 49.19, 38.65, 29.30, 29.04, 21.69, 15.62, 14.37, 12.11 ppm. HRMS (ESI^+^): m/z (M + H)^+^ calculated for C_22_H_30_N_3_O_6_^+^, 432.2129; found, 432.2214.

*1.2.30 Cyclohexyl-6-diazo-2-(3-methyl-3-(2,4,5-trimethyl-3,6-dioxocyclohexa-1,4-dien-1-yl)-butanamido)-5-oxohexanoate (****10g****).*

As described in the preparation of **10c**, compound **10g** was prepared by using **9a** and **5c** instead of **9d** and **5a**, respectively, and the yield was 46.0%. mp 122−124 °C. ^1^H NMR (300 MHz, CDCl_3_): *δ* 4.83−4.75 (m, 1H), 4.51−4.45 (m, 1H), 2.85 (s, 6H), 2.48−2.31 (m, 2H), 2.16 (s, 3H), 2.01−2.00 (m, 6H), 1.87−1.84 (m, 2H), 1.75−1.70 (m, 5H), 1.48 (s, 3H), 1.47 (s, 3H) ppm. ^13^C NMR (75MHz, CDCl_3_): *δ* 171.87, 171.13, 153.12, 143.48, 137.96, 137.85, 137.04, 128.18, 125.24, 74.20, 69.22, 66.28, 51.67, 49.00, 38.61, 38.28, 31.40, 28.92, 28.78, 25.20, 23.57,14.14, 12.71, 12.13 ppm. HRMS (ESI^+^): m/z (M + H)^+^ calculated for C_26_H_35_N_3_O_6_^+^, 486.2599; found, 486.2694.

*1.2.31 3-Phenylpropyl-6-diazo-2-(3-methyl-3-(2,4,5-trimethyl-3,6-dioxocyclohexa-1,4-dien-1-yl)-butanamido)-5-oxohexanoate (****10h****).*

As described in the preparation of **10d**, compound **10h** was prepared by using **9a** and **5d** instead of **9c** and **5a**, respectively, and the yield was 35.0%. mp 125−127 °C. ^1^H NMR (300 MHz, CDCl_3_): *δ* 7.24−7.19 (m, 5H), 4.53−4.50 (m, 1H), 4.07−4.03 (m, 2H), 3.77−3.73 (m, 2H), 2.40−2.38 (m, 2H), 2.34 (s, 9H), 2.23−2.10 (m, 4H), 1.95−1.91 (m, 2H), 1.24 (s, 6H) ppm. HRMS (ESI^+^): m/z (M + Na)^+^ calculated for C_29_H_35_N_3_O_6_Na^+^, 544.2524; found, 544.2500.

2. Clone formation assay.

Cancer cells were inoculated at 500 cells per well in six-well plates and cultured in 5% CO_2_ at 37 °C. The compounds were formulated into a 10^4^ μM master mix with DMSO and diluted to the appropriate concentration with the corresponding medium containing 10% FBS. Cells were replaced with fresh drug-containing medium about one week after inoculation, and incubated at 5% CO_2_, 37 ℃. After incubation, the cells were washed with PBS, 1 mL of methanol was added to each well to fix the cells for 30 min. Then, the methanol was discarded, and the cells were stained by adding 1 mL of crystal violet staining solution to each well for 30 min, washed with PBS, poured out minus the water, and then air-dried to be photographed and counted.

3. Analysis of siRNA.

siRNA (GenePharma) were transfected using the transfection agent jetPRIME® (Polypuls) according to the manufacturer's instructions. Gene knockdown was detected by RT-qPCR or Western Blot analysis after transfection for 48-72 h. A siRNA with a scrambled sequence served as the negative control. The sequences of synthetic siRNAs were listed in Extended Data Table 6.

4. Cell Counting Kit-8 assay (CCK8).

For analysis of cell cytotoxicity, cancer cells were seeded in 96-well plates with 100 μL/well and incubated at 5% CO_2_, 37 ℃ for 24 h. Then, cells were treated with drugs for 72 h. After that, 20 μL CCK8 solution was added to each well, and the absorbance (OD450) was measured at 450 nm after 2 h of incubation. For analysis of cell proliferation, cells were inoculated in 96-well plates and incubated for 0, 24, 48, 72, and 96 h. After reaching the indicated time, 20 μL CCK8 was added to each well, and the absorbance (OD450) was separately measured at 450 nm. The relevant formula was calculated as: cell viability = [(As-Ab) / (Ac-Ab)] × 100%, As: absorbance of experimental wells, Ac: absorbance of control wells, Ab: absorbance of blank wells.

5. Transwell.

In order to detect the ability of cells to migrate and invade, Matrigel matrix (Corning) was diluted with serum-free medium at 4 ℃, and the upper surface of PC membrane was permeate with or without matrigel matrix. After drying, Cells were seeded into 6.5 mm transwell chamber with 8.0 μm pore polycarbonate membrane insert (PC, CLS3422). Medium containing 10% FBS was used in the lower chamber, and cells were cultured with serum-free medium for 24 h in the transwell chamber. The cells were fixed with 4% paraformaldehyde and stained with crystal violet solution. The migrated and invaded cells were photographed by inverted microscope (Nikon ECLIPSE Ts2).

6. ROS assay.

Cells were seeded in the plates, cultured until adherent, and treated with drugs for 24 h. Fluorescence probe DCFH-DA was loaded in situ using the Reactive Oxygen Species Assay Kit (Beyotime Biotechnology) according to manufacturer's instructions, incubated in the dark for 30 min, and cells were washed with serum-free medium. Random fields were photographed by inverted microscope (Nikon ECLIPSE Ts2), and fluorescence intensity was detected by fluorescent microplate reader (Molecular Devices). ROS content was measured by flow cytometry (FACSCelesta) in parallel group.

7. Mitochondrial membrane potential assay.

Cells were seeded in the plates, cultured until adherent, and treated with drugs for 24 h. Using the Mitochondrial Membrane Potential Assay Kit with fluorescence probe JC-10 (Boxbio) according to the manufacturer's instructions, JC-10 probe was incubated for 30 min, and cells were washed with JC-10 staining buffer. Random fields were photographed by inverted microscope (Nikon ECLIPSE Ts2), and fluorescence intensity was detected by fluorescent microplate reader (Molecular Devices).

8. Metabolomics.

For the analysis of metabolomics, samples were prepared and analyzed using Agilent 1290 Infinity LC ultra-high performance Liquid Chromatography (UHPLC) system coupled with an AB 6500+ QTRAP Mass Spectrometer (AB SCIEX) for metabolite profiling. Metabolic separation was performed using an ACQUITY UPLC BEH Amide column (2.1×100 mm, 1.7 µm, Waters) and an ACQUITY UPLC BEH C18 column (2.1×100 mm, 1.7 µm, Waters). ESI source related parameters were set as follows: source temperature, 580 ℃; ion source gas1 (GS1), 45 psi; ion source gas 2 (GS2), 60 psi; curtain gas (CUR), 35 psi; ion spray voltage (IS): +4500 V(ESI+) or -4500V(ESI-); MRM mode monitoring. QC samples were inserted into the sample queue to monitor and evaluate the stability of the system and the reliability of the experimental data. After the samples were thawed slowly at 4 ℃, an appropriate amount of samples was added to pre-cooled methanol/acetonitrile/water solution (2:2:1, v/v/v), vortexed and mixed, sonicated at low temperature for 30 min, left to stand at -20 ℃ for 10 min, and then centrifuged at 14,000 g at 4 ℃ for 20 min, and the supernatant was dried in vacuum, and then added to 100μL of acetonitrile and water solution (acetonitrile : water=1:1, v/v) to re-dissolve it for mass spectrometry, vortexed, and then centrifuged at 14,000 g at 4 ℃ for 15 min. For LC-MS analysis, add 100 μL of acetonitrile aqueous solution (acetonitrile : water=1:1, v/v), vortex, centrifuge at 14,000 g at 4 ℃ for 15 min, and then take the supernatant to analyze.

9. In vitro NQO1 reduction assay.

Compound 10a-10h were monitored as NQO1 substrates using an NADPH recycling assay. NADPH oxidation to NADP+ was monitored by absorbance (A340nm) on a Multiskan Sky (Thermo Scientific). Compounds were dissolved in DMSO (10 mM) and NQO1 (1.4μg/mL) in 50 mM potassium phosphate buffer (pH 7.2-7.4) were added into each well (198μL). It was shaken for 2 min and placed into the incubator at 37 °C for 3 min before initiating the reaction with NADPH solution (400μM), and the absorbance change at 340 nm was recorded at 2 s intervals for 2 min at room temperature. The linear portion of the absorbance versus time graphs (the first 20 s to 1 min) were fitted and the slopes were calculated (velocity). Initial velocities were calculated and results expressed as μmol NADPH oxidized/min/μmol protein.

10. Plasma stability and intestinal stability.

Compounds (0.1 mg) were added into 1 mL of human or rat plasma and the mixture was shaken at 37 °C for 1 h. Compounds (0.1 mg) were added to 1 mL of artificial intestinal fluid containing potassium dihydrogen phosphate and pancreatin (PH = 6.8) and the mixture was shaken at 37 °C for 1 h. The biological reaction was terminated with three times the volume of cold acetonitrile. The suspensions were filtered and determined by HPLC (Agilent 1260 infinity) in 254 nm. Quantification of each compound was calibrated by comparison with linear standard curve established from standard solution in acetonitrile. Determination was performed in duplicate.

11. Stability assay and the activation on enzyme level.

10e was diluted to 20μM with PBS (0.05 M, pH = 7.2-7.4) which was incubated with NQO1 enzyme (14 µg/mL) and NADPH (100μM) for 1 h at 37 ℃. The reaction was terminated by adding three times volume of cold acetonitrile, and the supernatants were collected by centrifugating in a refrigerated centrifuge at 12500 rpm at 4 ℃ for 25 min. Then, the release of the compound and metabolites was detected with HPLC (Agilent 1260 infinity) and mass spectrometry (Agilent G6520B).

12. Human tumor cell-to-plasma partitioning assays.

Tumor cells in the logarithmic growth phase were collected, prepared into cell suspensions and washed , and cells were collected by centrifugation at 1000 rpm at 25 ℃ for 5 min, washed with 20 mL PBS, following which cells were resuspended in human plasma (Innovative Research) to obtain a cell density of 10 million cells/mL. For analysis of cell partitioning, 1 mL of the cell-plasma suspension was preincubated at 37 °C for 5 min, following which the 20μM prodrug was added and re-incubated at 37 °C for 1 h. After incubation, the cell suspension was centrifuged at 1000 rpm at 4 °C for 10 min and supernatant plasma was collected and stored at -80 °C for bioanalysis. The cell pellet was washed once with ice-cold PBS, followed by centrifugation and stored at -80 °C for prodrug and/or DON bioanalysis.

13. Bioanalysis of DON.

DON was extracted from samples (50 mL/mg) with 250μL methanol by vortexing in low retention tubes. Samples were centrifuged at 16,000 g for 5 min to precipitate proteins. Supernatants (200μL) were moved to new tubes and dried at nitrogen atmosphere for 1 h. To each tube, 50μL of 0.2 M NaHCO_3_ buffer (pH 9.0) and 100μL of 10 mM dabsyl chloride in acetone were added. After vortexing, samples were incubated at 60 °C for 15 min to derivatize. To extract the derivative, the mixture was added into 300μL ethyl acetate, shaken violently and centrifuged at 12000 rpm for at 4 °C 10 min, while the 200μL collected supernatant dried with nitrogen. Finally, residue was redissolved in methanol with 10 ng/mL diazepam. Samples (10μL) were injected and separated on a SHIMADZU LCMS-8045.


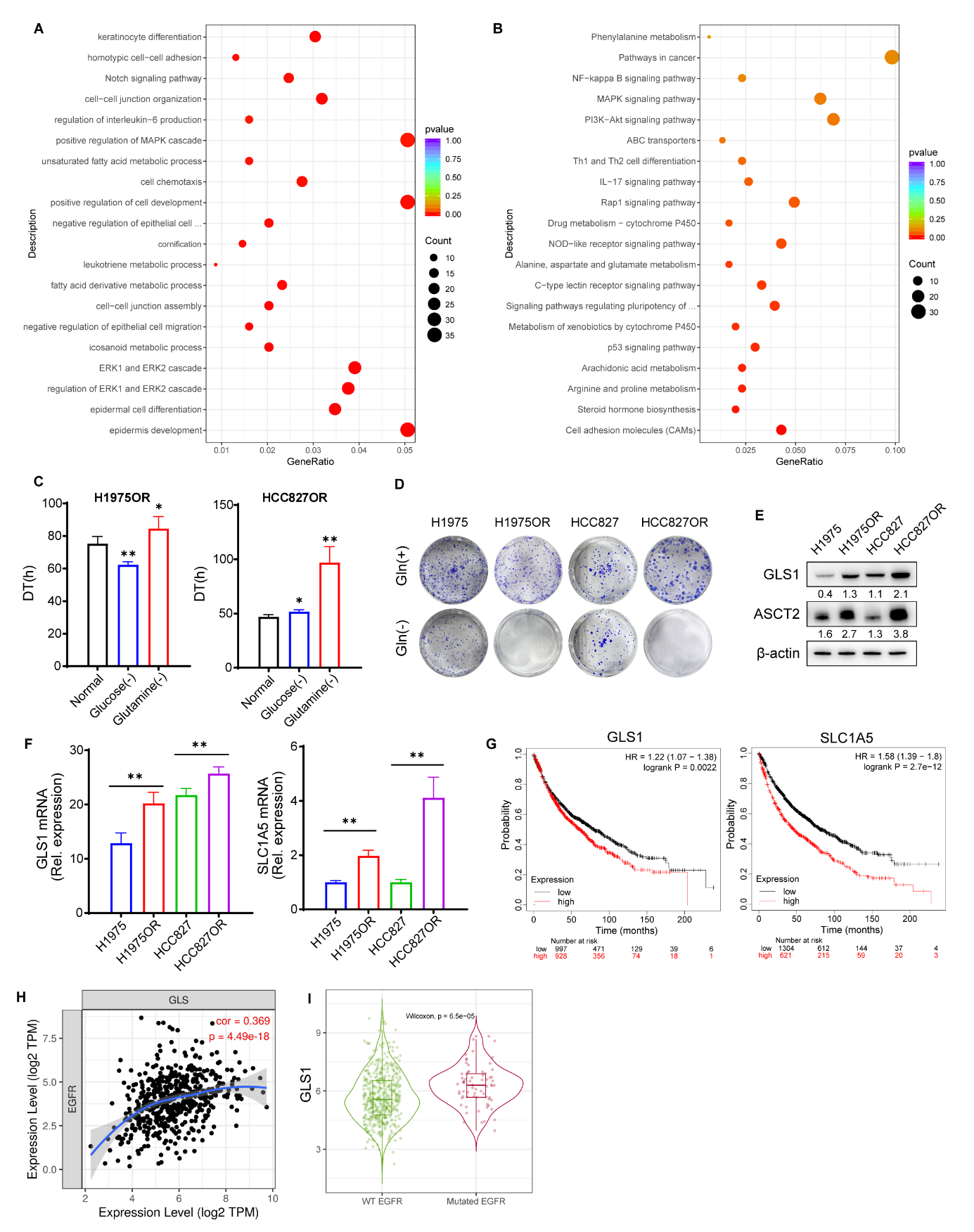


**Figure S1**: Reprogramming of glutamine metabolism in cells resistant to Osimertinib.

A, B) KEGG pathway enrichment analysis (A) and GO functional enrichment analysis (B) of upregulated gene modules in H1975OR cells (n=3 per group). C) Quantification of doubling time (DT) of Osimertinib-resistant cells in standard, glucose-deprived and glutamine-deprived culture conditions (n=6 per group). D) Colony formation of H1975, H1975OR, HCC827, and HCC827OR cells in standard and glutamine-deprived culture conditions. E) Protein expression of GLS1 and ASCT2 in H1975, H1975OR, HCC827, and HCC827OR cells determined by Western Blot. β-Actin served as a loading control. F) mRNA expression of GLS1 and SLC1A5 in H1975, H1975OR, HCC827, and HCC827OR cells determined by qPCR analysis (n=6 per group). G) Kaplan-Meier curve showing overall survival of lung cancer patients with high or low GLS and SLC1A5 expression (Source: OncoLnc, http://www.oncolnc.org/). H) Correlation analysis of GLS and EGFR in lung cancer (Source: TIMER, https://cistrome.shinyapps.io/timer/). I) GLS gene levels in both wild-type EGFR and mutated EGFR lung cancer (Source: TIMER, https://cistrome.shinyapps.io/timer/). Data are presented as mean ± SD, calculated using two-sided unpaired Student’s t-test, * *P* < 0.05, ** *P* < 0.01.


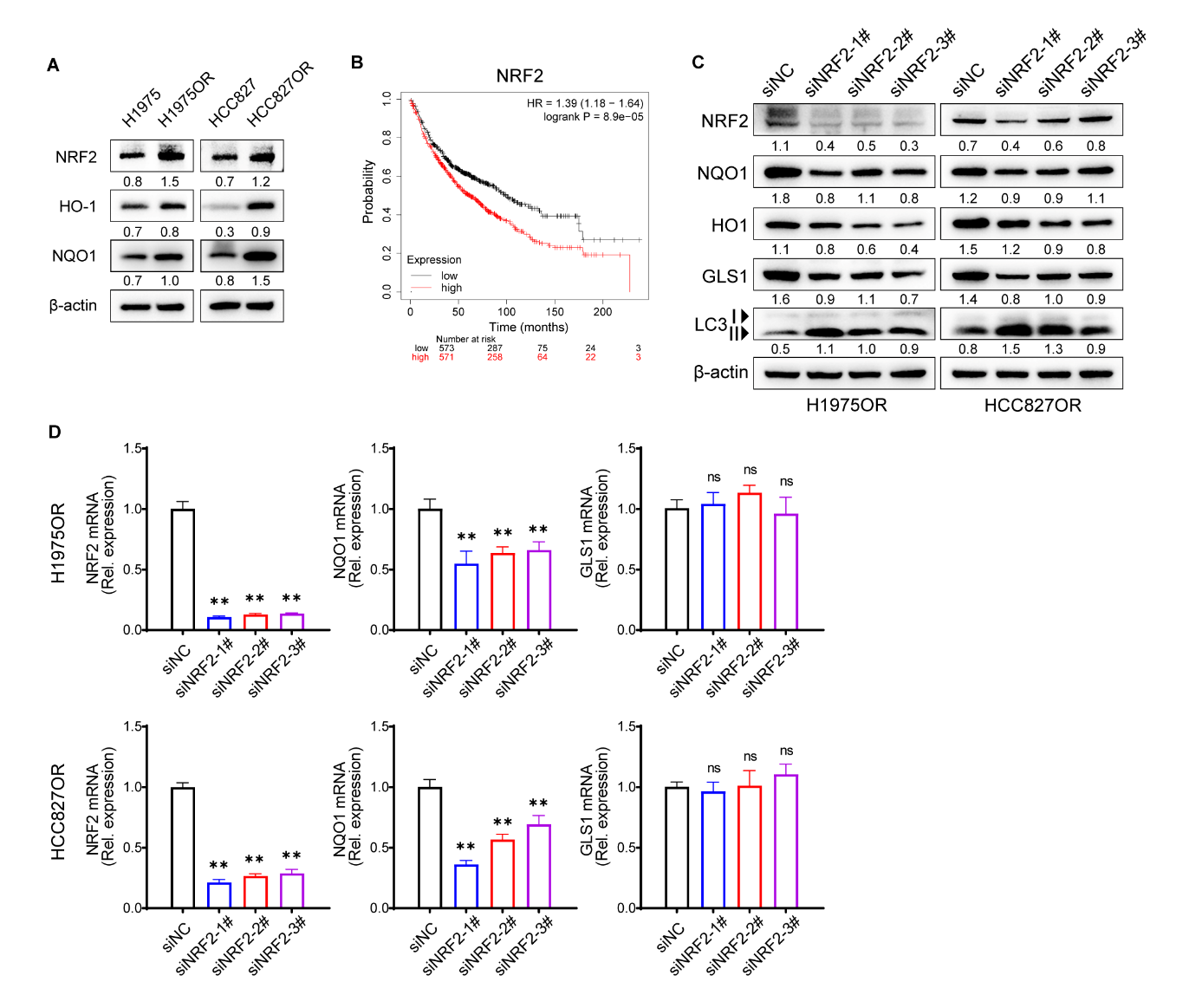


**Figure S2**: Correlation between NRF2 and GLS1 protein.

A) Protein expression of NRF2, HO-1 and NQO1 in H1975, H1975OR, HCC827, and HCC827OR cells determined by Western Blot. β-Actin served as a loading control. B) Kaplan-Meier curve showing overall survival of lung cancer patients with high or low NRF2 expression (Source: OncoLnc, http://www.oncolnc.org/). C) Western Blot analysis of relevant proteins in Osimertinib-resistant cells following siNC, siNRF2-1#, siNRF2-2#, siNRF2-3# treatments. D) qPCR analysis of relevant genes in Osimertinib-resistant cells following siNC, siNRF2-1#, siNRF2-2#, siNRF2-3# treatments (n=6 per group). Data are presented as mean ± SD, calculated using two-sided unpaired Student’s t-test, * *P* < 0.05, ** *P* < 0.01, ns not significant, *P* > 0.05.


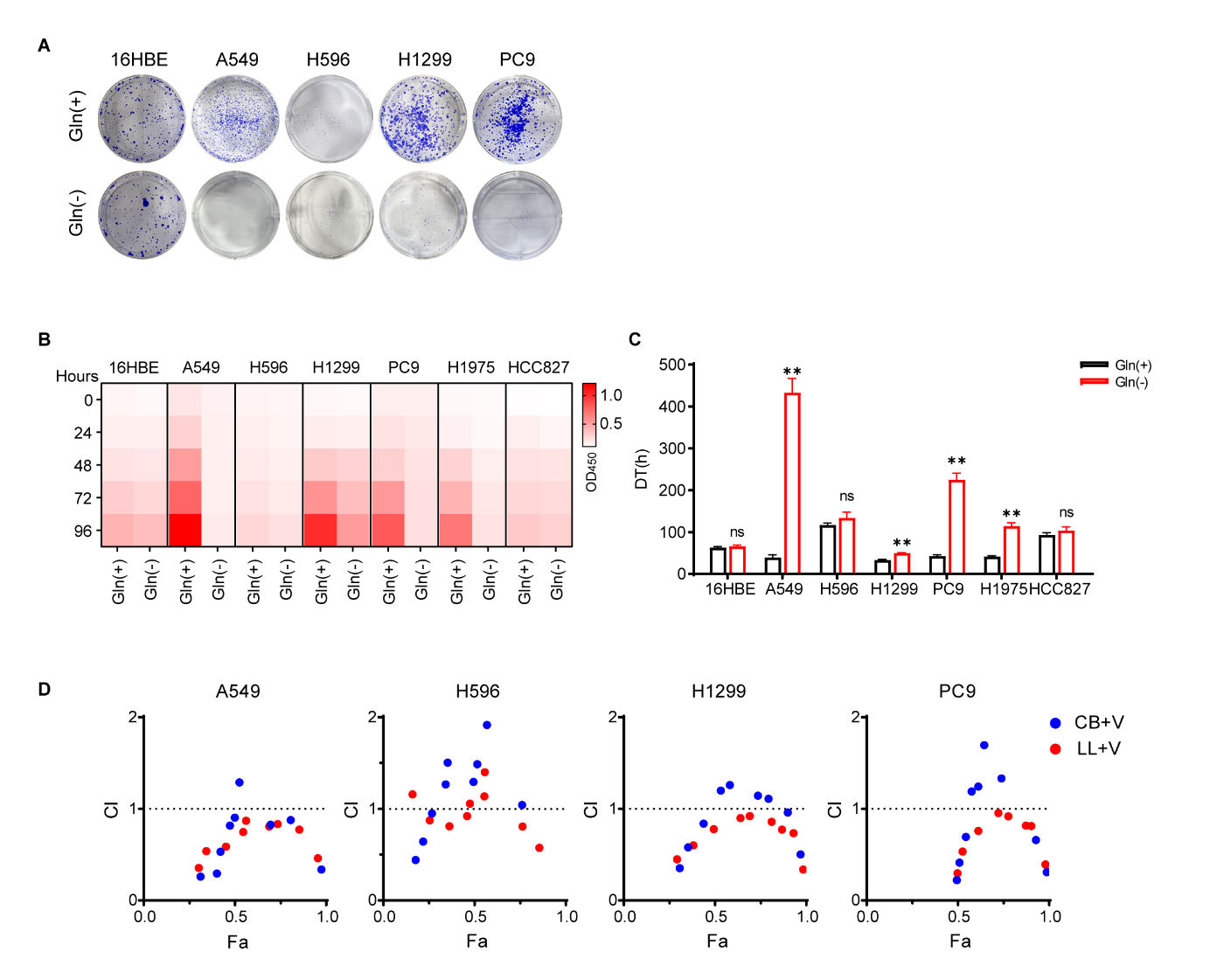


**Figure S3**: Detection of glutamine deprivation and inhibition in lung cancer cells.

A) Colony formation of lung cancer cells in standard and glutamine-deprived culture conditions. B, C) CCK8 assay showing cell activity in normal, glucose-deprived and glutamine-deprived cultures (B), and quantification of DT (C) (n=4 per group). D) Synergistic effect of GLS1 inhibitor with ASCT2 inhibitor on lung cancer cells (n=9 per group). Data are presented as mean ± SD, calculated using two-sided unpaired Student’s t-test, * *P* < 0.05, ** *P* < 0.01, ns not significant, *P* > 0.05.


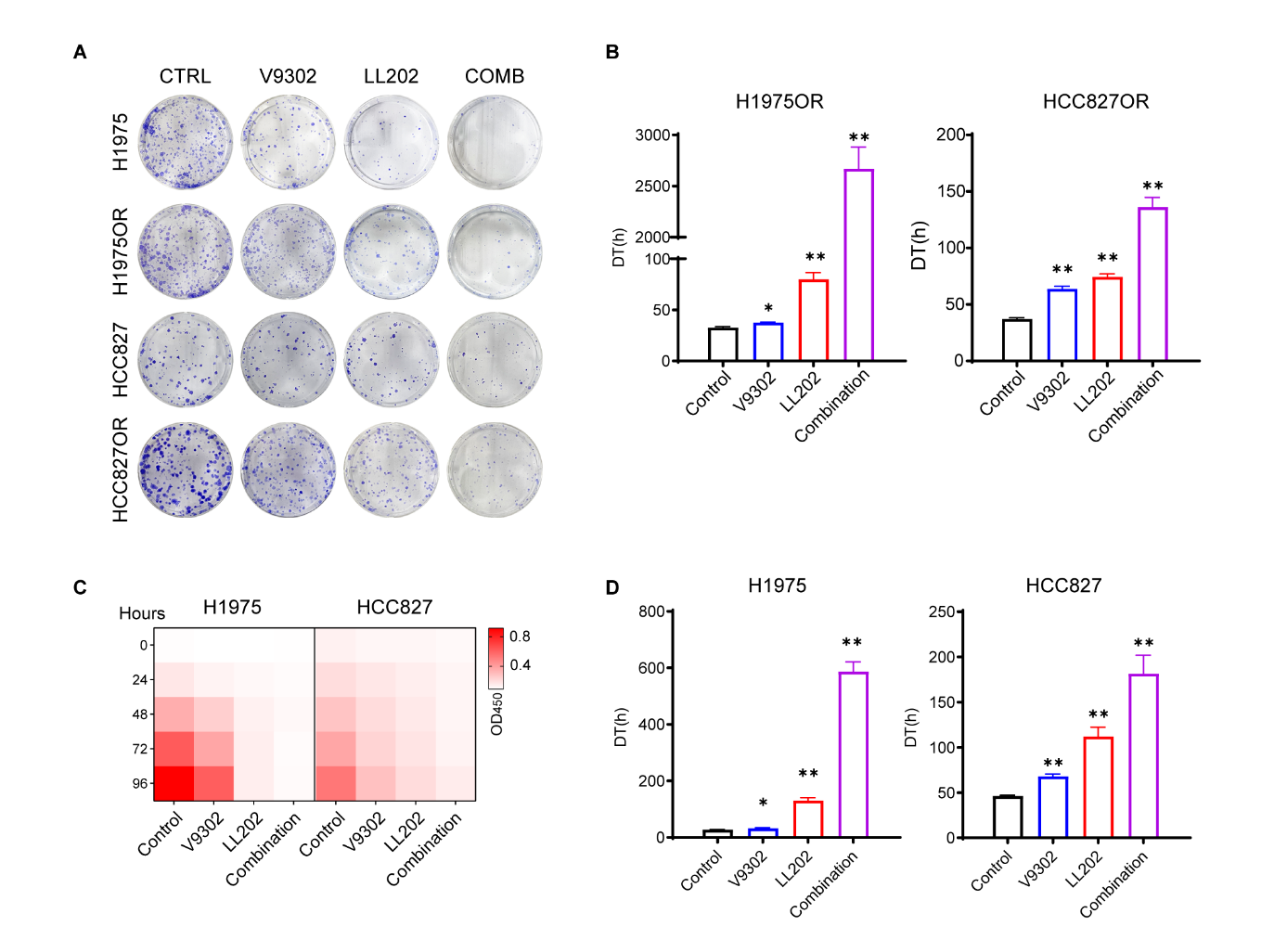


**Figure S4**: Enhanced inhibitory effect of combination therapy LL202 plus V9302 on lung cancer cells.

A) Colony formation of H1975, H1975OR, HCC827, HCC827OR cells following administration of combination therapy. B) Quantification of Osimertinib-resistant cells DT following administration of combination therapy (n=3 per group). C, D) CCK8 assay showing H1975, HCC827 cell activity following administration of combination therapy (C), quantification of DT (D) (n=3 per group). Data are presented as mean ± SD, calculated using two-sided unpaired Student’s t-test, * *P* < 0.05, ** *P* < 0.01.


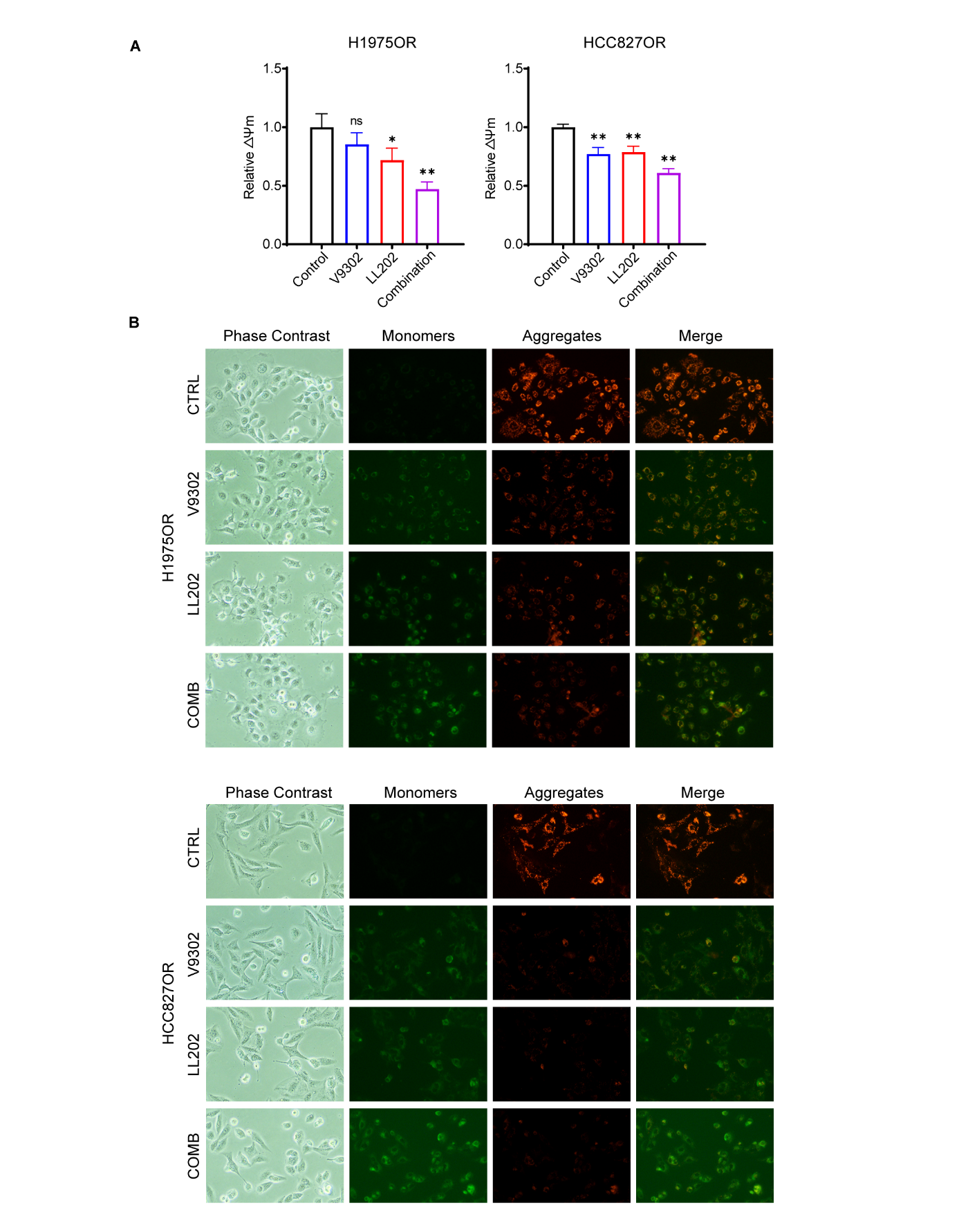


**Figure S5**: The combination therapy elicits apoptosis in cells resistant to Osimertinib.

A, B) Multifunctional microplate reader (A) and fluorescence microscope (scale bars, 500μm) (B) shows mitochondrial membrane potential (△Ψm) in Osimertinib-resistant cells following administration of combination therapy by JC-10 staining (n=3 per group). Data are presented as mean ± SD, calculated using two-sided unpaired Student’s t-test, * *P* < 0.05, ** *P* < 0.01.


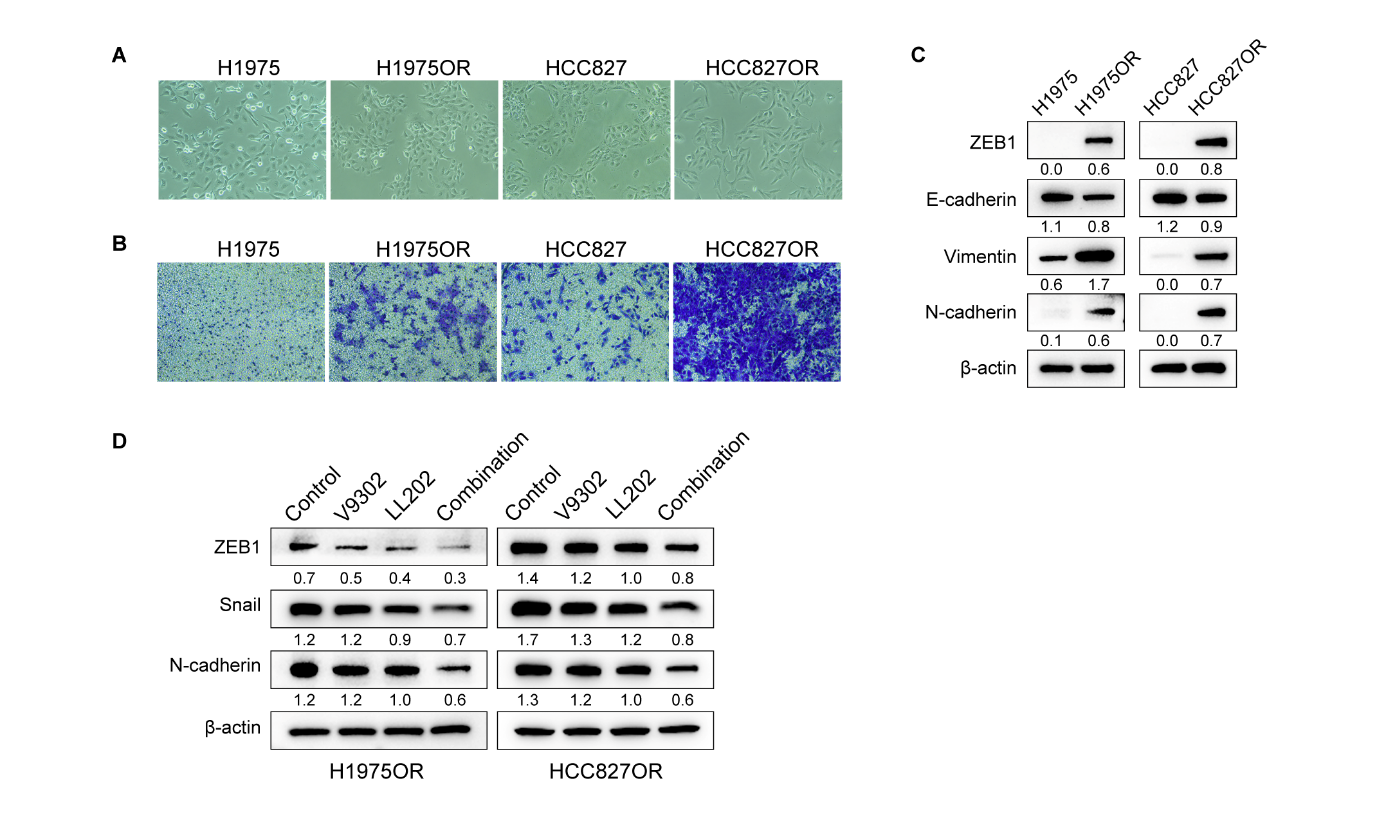


**Figure S6**: Combination therapy suppresses epithelial-mesenchymal transition in Osimertinib-resistant cells.

A, B) The cellular morphology (A) and cell migration (B) of H1975, H1975OR, HCC827, and HCC827OR cells were examined using microscope and transwell assay (scale bars, 100μm). C, D) Western Blot analysis for proteins in H1975, H1975OR, HCC827, HCC827OR cells (C), as well as the changes in protein expression in Osimertinib-resistant cells following administration of combination therapy (D).


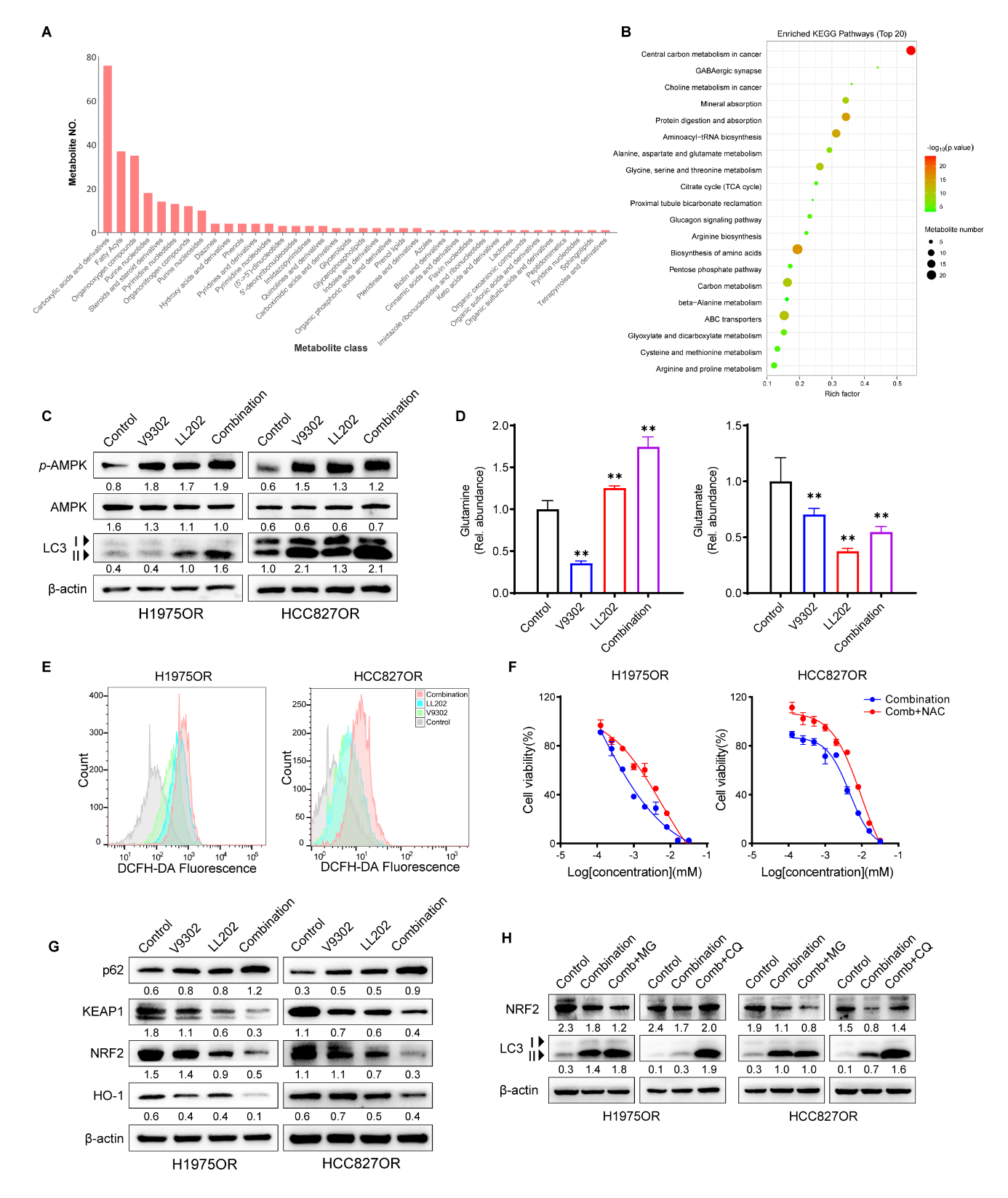


**Figure S7**: The combination therapy perturbs the redox homeostasis and disrupts the energy balance.

A, B) The number of metabolites identified in each chemical classification (each column in the figure represents a different chemical classification attribution entry) was counted after treatment of Osimertinib-resistant cells with V9302 and LL202 alone or in combination (A), the metabolic pathways that were significantly up-regulated were then analyzed by KEGG enrichment (B). C, G) Western Blot analysis for proteins in Osimertinib-resistant cells following administration of combination therapy. D, E) Quantification of glutamine and glutamate levels (D) in Osimertinib-resistant cells following administration of combination therapy, flow cytometry analysis for cell apoptosis (E) (n=3 per group). F) CCK8 assay showing cell activity of Osimertinib-resistant cells following administration of combination therapy with or without NAC (n=3 per group). G, H) Western Blot analysis for NRF2 and NQO1 protein following time-dependent combination treatment (G), NRF2 and LC3 proteins in cells treated with combination therapy, with or without the action of MG132 and CQ (H). Data are presented as mean ± SD, calculated using one-way analysis of variance (ANOVA), followed by Dunnett’s multiple comparison test. * *P* < 0.05, ** *P* < 0.01.


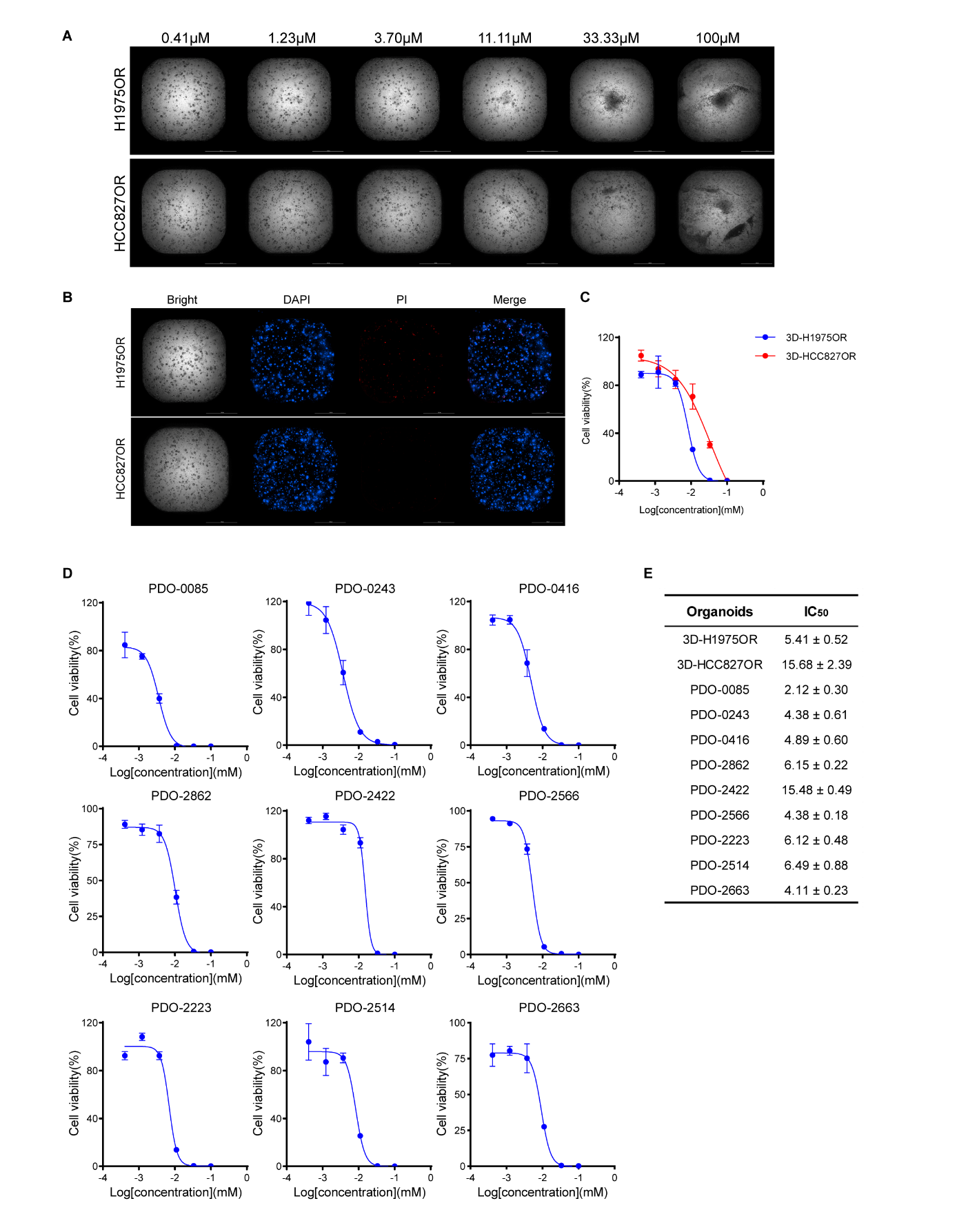


**Figure S8**: Enhanced inhibitory effect of LL202 plus V9302 on lung cancer PDOs.

A) Microscope showing the morphology of 3D-H1975OR and 3D-HCC827OR cells after time-dependent concentration of combination treatment. B) Fluorescence microscope showing apoptosis of 3D-H1975OR and 3D-HCC827OR cells after combination treatment. C-E) Cell viability of 3D cells (C) and PDOs (D) were measured after treating with various concentrations combination treatment, and calculated IC_50_ value (E) (n=3 per group). Data are presented as mean ± SD.


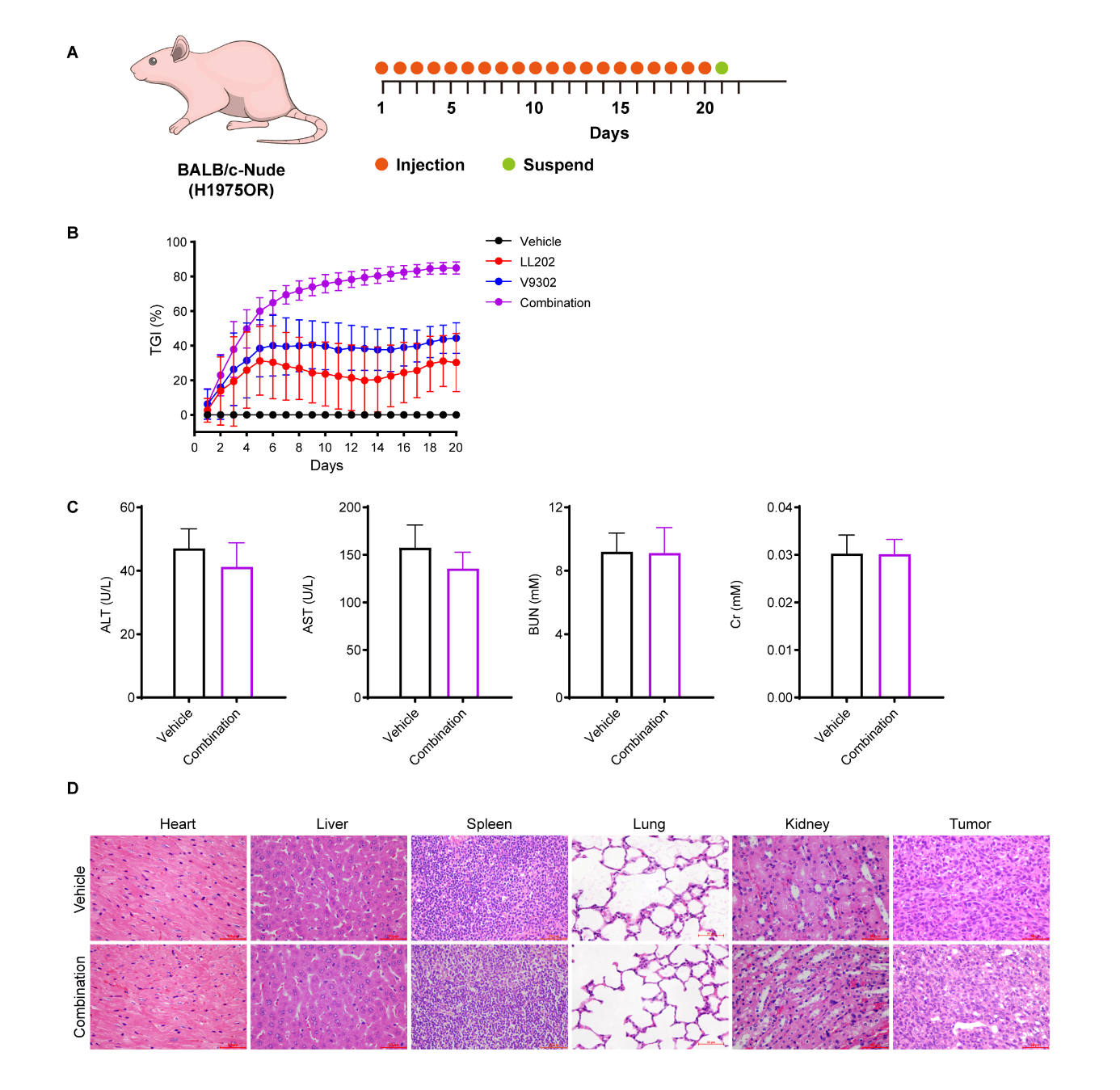


**Figure S9**: Enhanced inhibitory effect of LL202 plus V9302 on H1975OR tumor-bearing mice.

A-D) H1975OR-tumor bearing mice were treated with normal saline (Vehicle), LL202 (30 mg/kg), V9302 (30 mg/kg) and LL202+V9302 (30 mg/kg). BALB/c-Nude mice were inoculated with 1×10^7^ H1975OR cells, group administration according to the time points shown in the schematic diagram (A), quantification of TGI (B), ALT, AST, BUN and Cr (C), representative H&E images of the major organs and tumor (scale bars, 50μm) (D) (n=10 per group). Data are presented as mean ± SD.


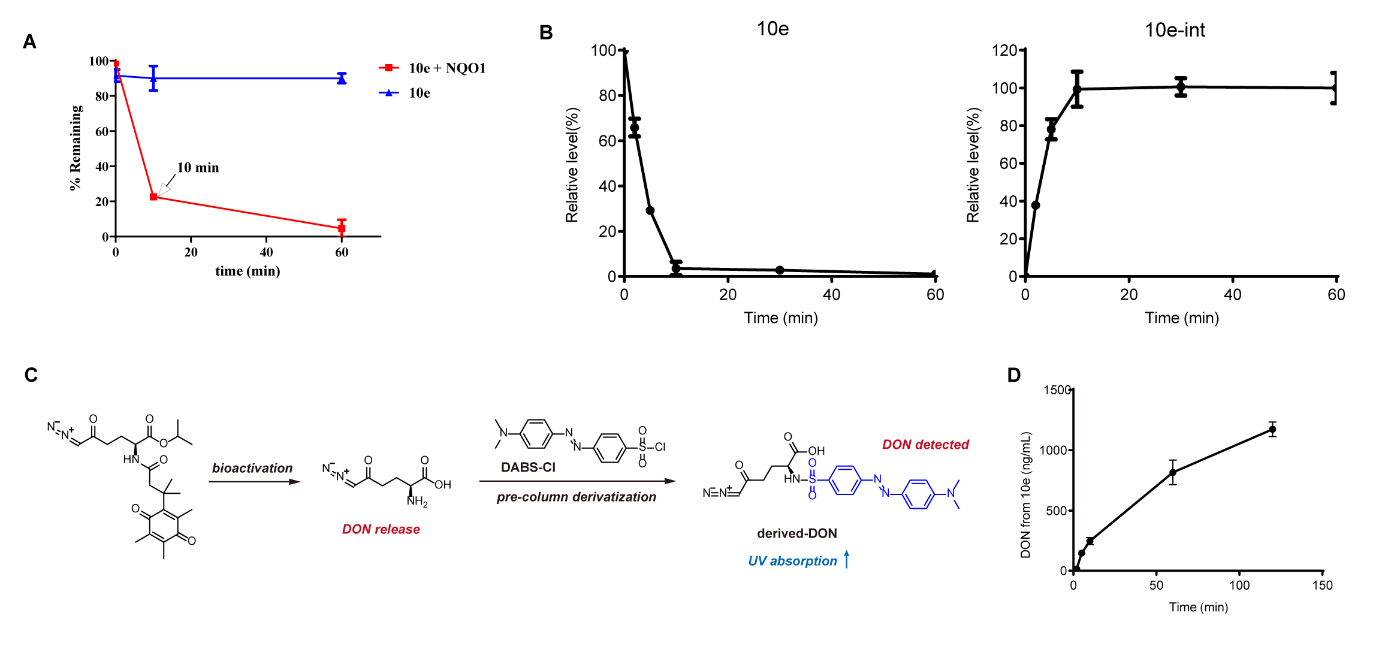


**Figure S10**: Pharmacokinetic analysis of 10e.

A) HRMS analysis for prodrug 10e in the presence of NQO1 after 1 h incubation (n=3 per group). B) After incubating 10e with rodent plasma for 0-60 minutes, the levels of 10e and its metabolic intermediate 10e-int were quantified using HPLC and HRMS, respectively (n=3 per group). C, D) 10e releases DON in response to NQO1 and carboxylesterase (n=3 per group). Data are presented as mean ± SD.


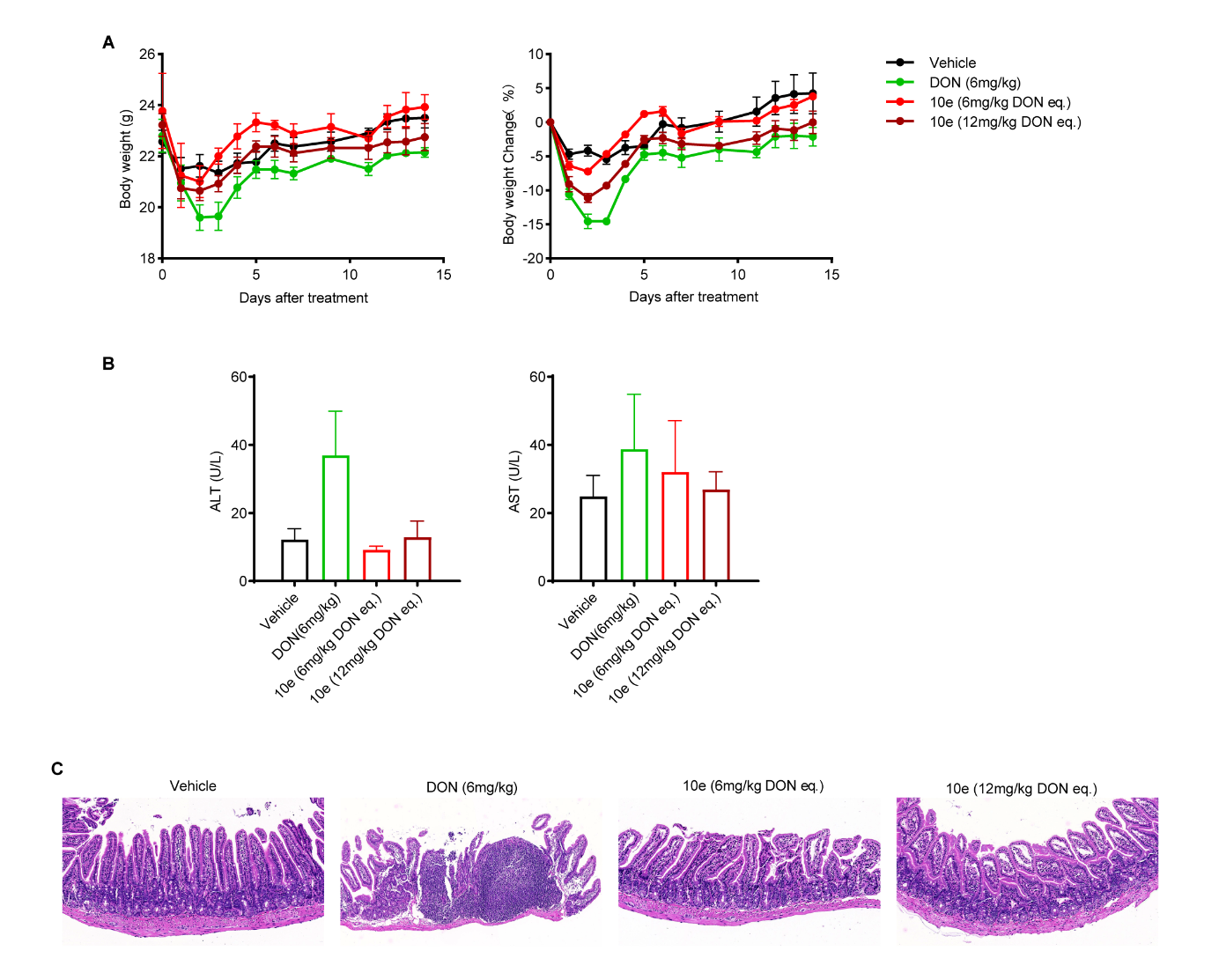


**Figure S11**: Acute toxicity assessment of the optimal prodrug 10e.

A-C) C57BL mice were treated with normal saline (Vehicle), DON (6 mg/kg), 10e (6 mg/kg DON eq.) and 10e (12 mg/kg DON eq.), and their body weight and changes in body weight (A) were continuously monitored over a 2-week period, the experiment was concluded after 2 weeks for the assessment of ALT, AST levels (B), and HE staining of the intestines (C) (n= 4 per group). Data are presented as mean ± SD.


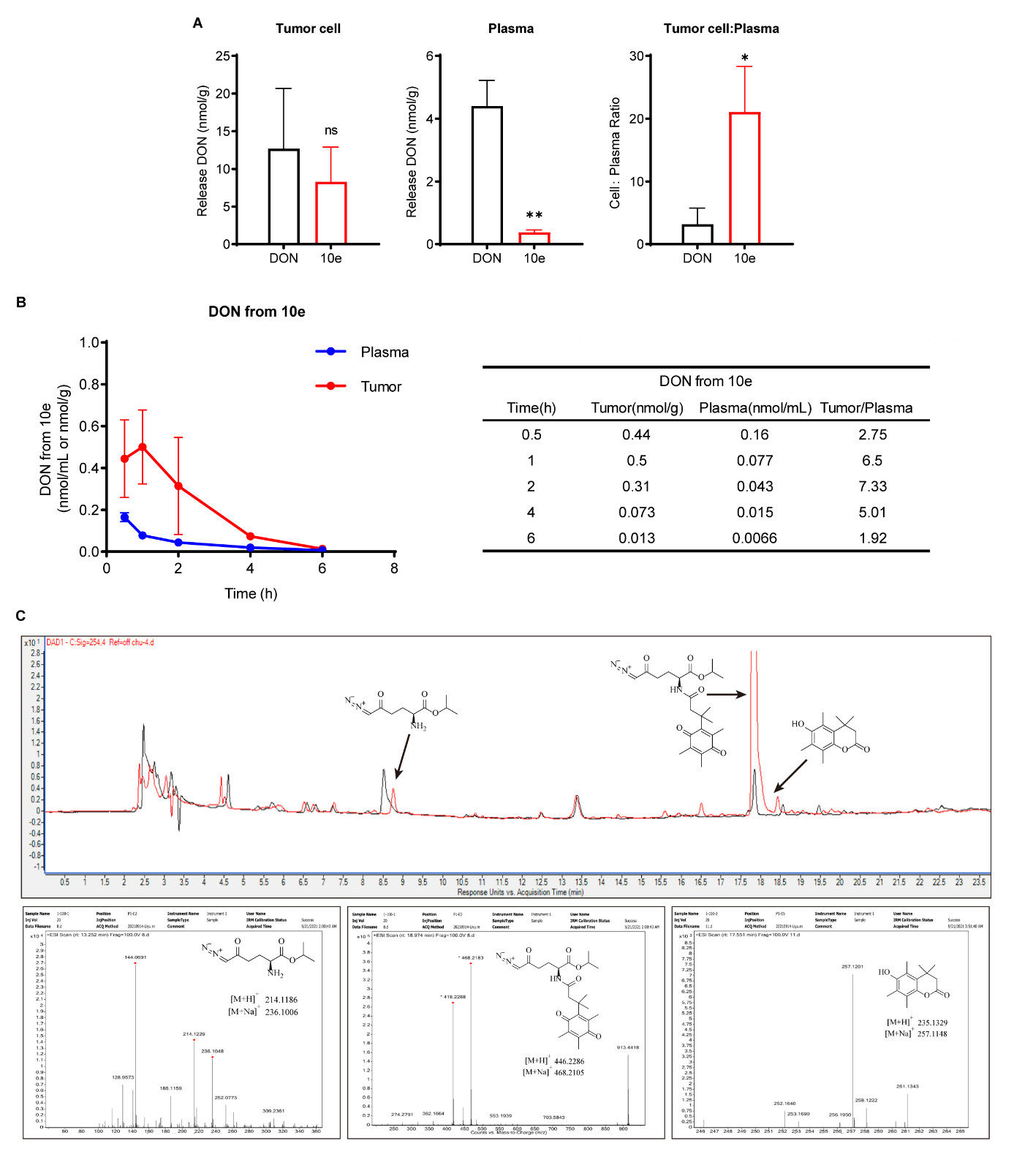


**Figure S12**: The tumor targeting assay of the optimal prodrug 10e.

A) After incubation for 1h in human tumor cells suspended in human plasma, both 10e and DON exhibited similar release of DON in tumor cells, with minimal release in human plasma. The partitioning ratio of DON from tumor cells to plasma was 3.18, while the ratio for 10e was 21.04 (n= 4 per group). B) Pharmacokinetic profile of DON release from 10e in tumor-bearing BALB/c mice (n= 4 per group). C) Metabolic identification (MET ID) of 10e in tumor cells, superimposed chromatograms and mass spectrogram of 10e and its metabolic intermediates. Data are presented as mean ± SD, calculated using two-sided unpaired Student’s t-test. * *P* < 0.05, ** *P* < 0.01, ns not significant, *P* > 0.05.


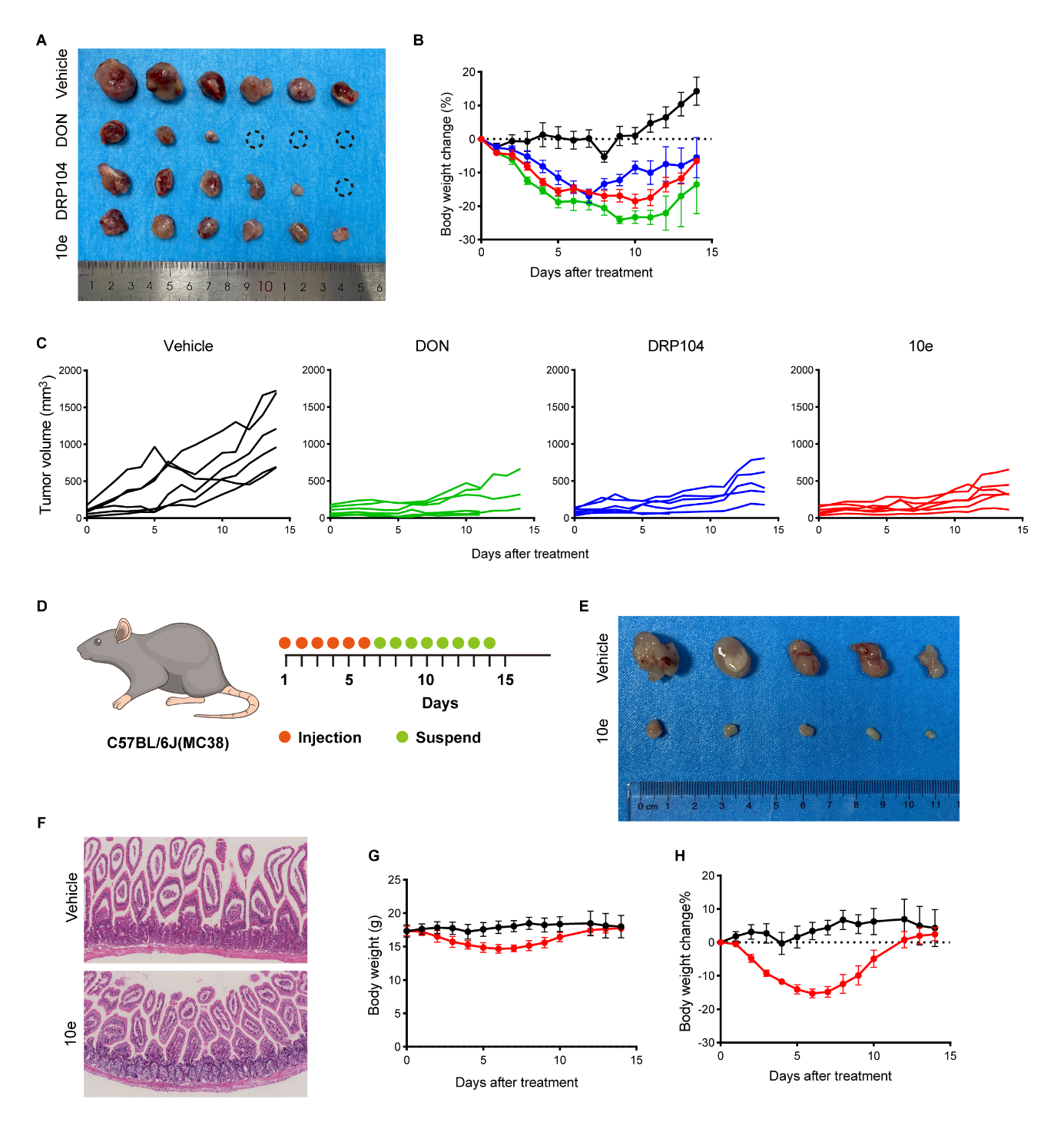


**Figure S13**: Assessment of the efficacy and safety of 10e in murine models bearing LLC and MC38 tumors.

A-C) LLC-tumor bearing mice were treated with normal saline (Vehicle), DON, DRP104 and 10e (0.5 mg/kg DON eq.). Tumors collected at the conclusion of the specified treatments, representative image depicting the tumors at the end of the treatment period is presented (A), quantification of body weight change (B) and tumor volume (C) throughout the specified treatments period (n= 6 per group). D-H) MC38-tumor bearing mice were treated with normal saline (Vehicle), and 10e (0.5 mg/kg DON eq.). C57BL mice were inoculated with MC38 cells, group administration according to the time points shown in the schematic diagram (D), tumor images (E) and intestinal HE-stained images (original magnification, ×20) (F) obtained at the conclusion of the indicated therapy, quantification of body weight (G) and body weight change (H) throughout the specified treatments period (n= 5 per group). Data are presented as mean ± SD.


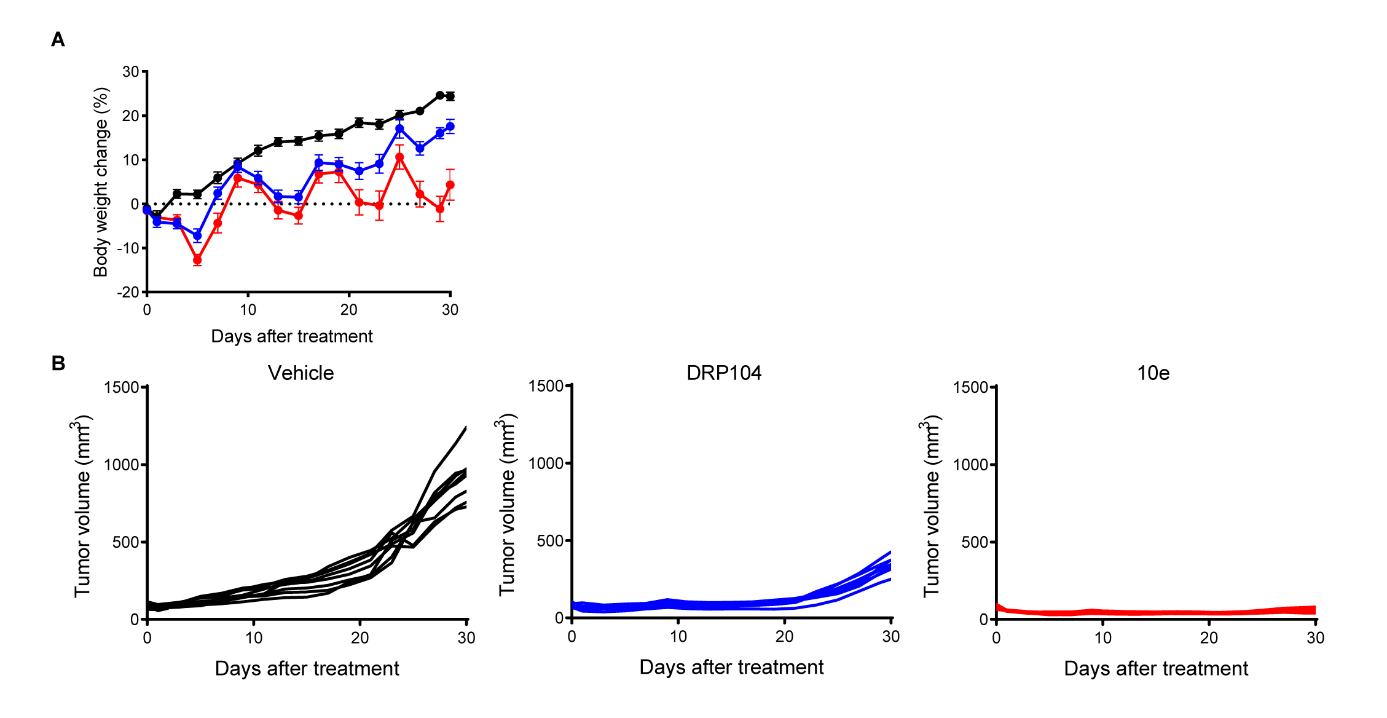


**Figure S14**: Analysis of efficacy and safety of 10e in the HCC827OR tumor mouse model.

A, B) HCC827OR-tumor bearing mice were treated with normal saline (Vehicle), DON, DRP104 and 10e (0.4 mg/kg DON eq.). Quantification of body weight change (A) and tumor volume (B) during the specified treatments period (n= 8 per group). Data are presented as mean ± SD.


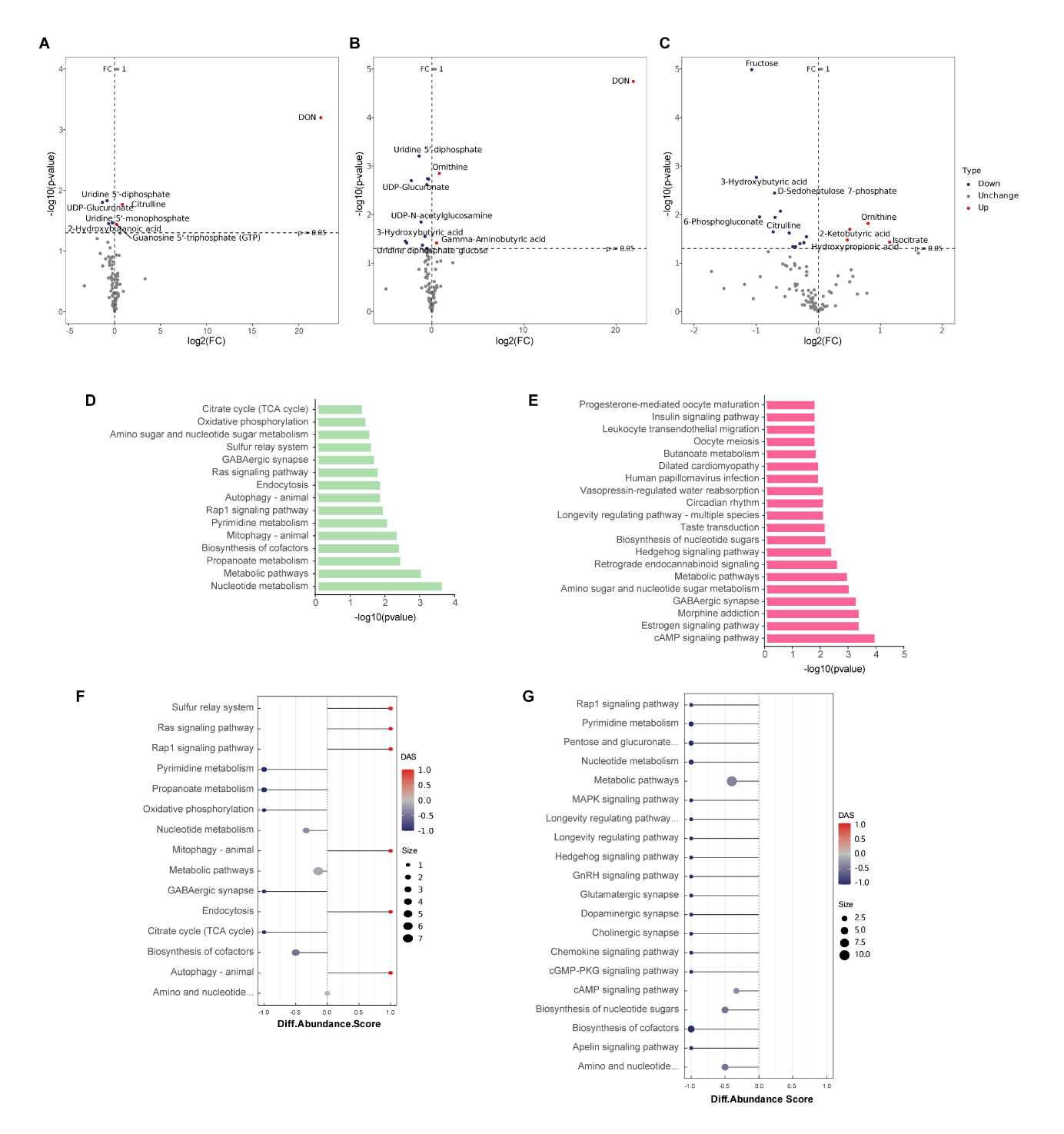


**Figure S15**: Analysis of metabolomics of 10e in the HCC827OR tumor mouse model.

A-G) HCC827OR-tumor bearing mice were treated with normal saline (Vehicle), DON, DRP104 and 10e (0.4 mg/kg DON eq.). Volcano plots depicting the differentially energy metabolites among DRP104 and Vehicle (A), 10e and Vehicle (B), as well as 10e and DRP104 (C). KEGG enrichment pathway map: DRP104 vs. Vehicle (D), 10e vs. Vehicle (E), DAS plots of all enriched metabolic pathways: DRP104 vs. Vehicle (F), 10e vs. Vehicle (G) (n= 4 per group).


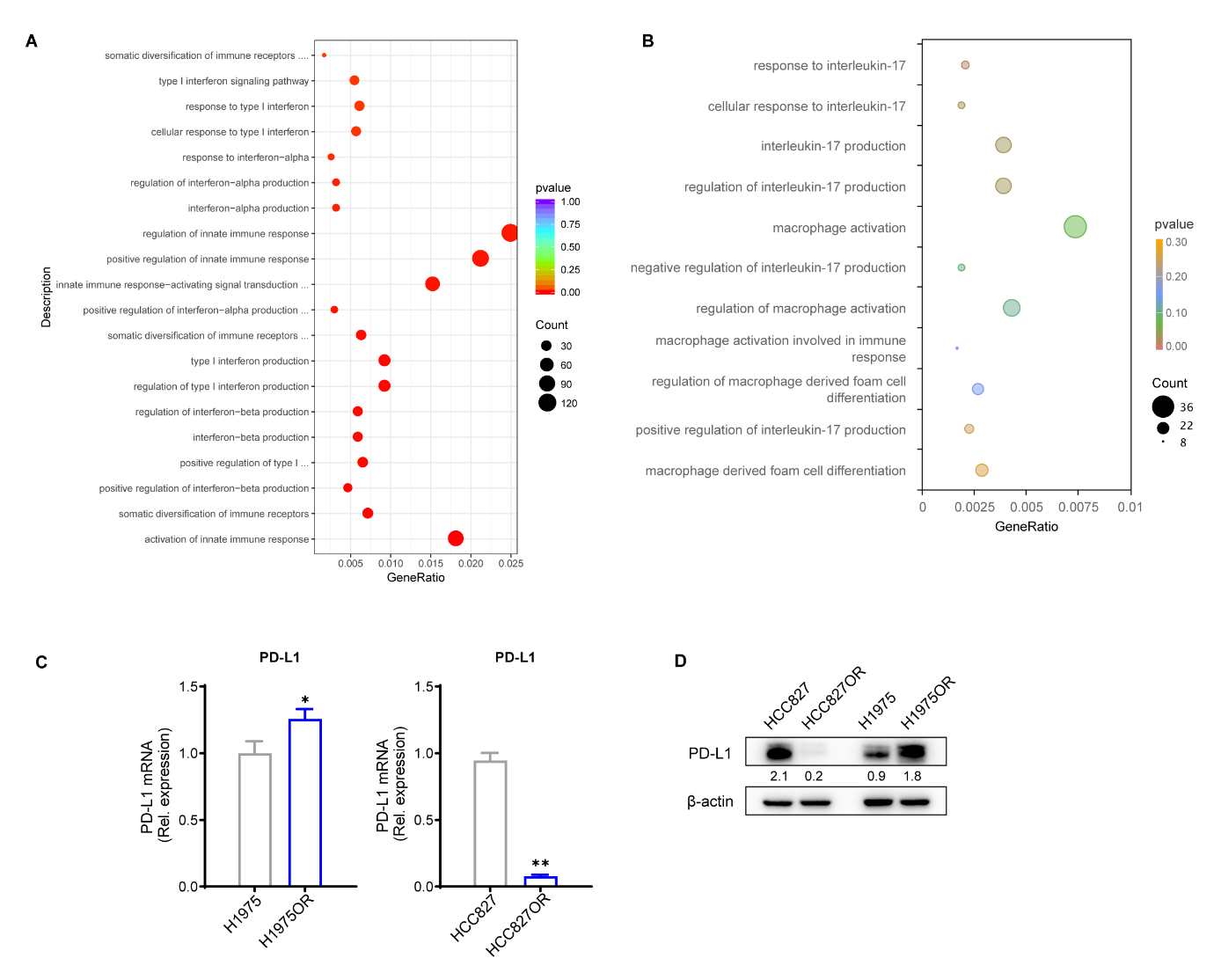


**Figure S16**: Immunoassay analysis of the HCC827OR model.

A, B) GO functional enrichment analysis of downregulated immune-related gene modules (A) and upregulated tumor-associated macrophage-related gene modules (B) in HCC827OR cells (n= 4 per group). C, D) Discrepancies in PD-L1 gene and protein expression between Osimertinib-resistant and parental cells (n= 3 per group). Data are presented as mean ± SD, calculated using two-sided unpaired Student’s t-test. * *P* < 0.05, ** *P* < 0.01.


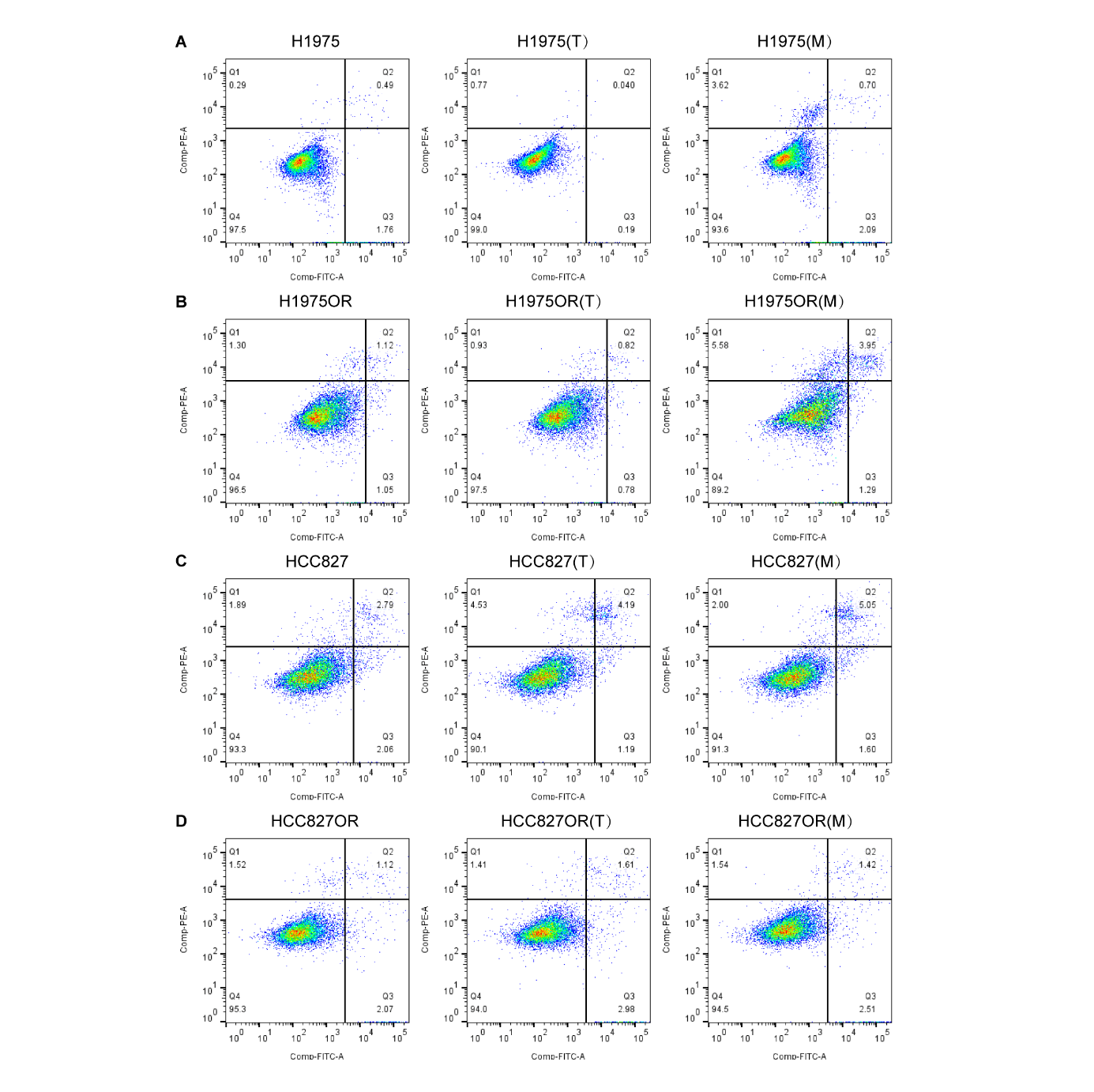


**Figure S17**: Flow cytometric analysis of apoptosis in lung cancer cells following co-culture with immune cells.

A-D) Co-cultures of H1975 (A), H1975OR (B), HCC827 (C), HCC827OR (D) and immune cells (T: T cells, M: macrophages) were subjected to treatment with 10e, representative flow cytometry plots showing tumor cells apoptosis.


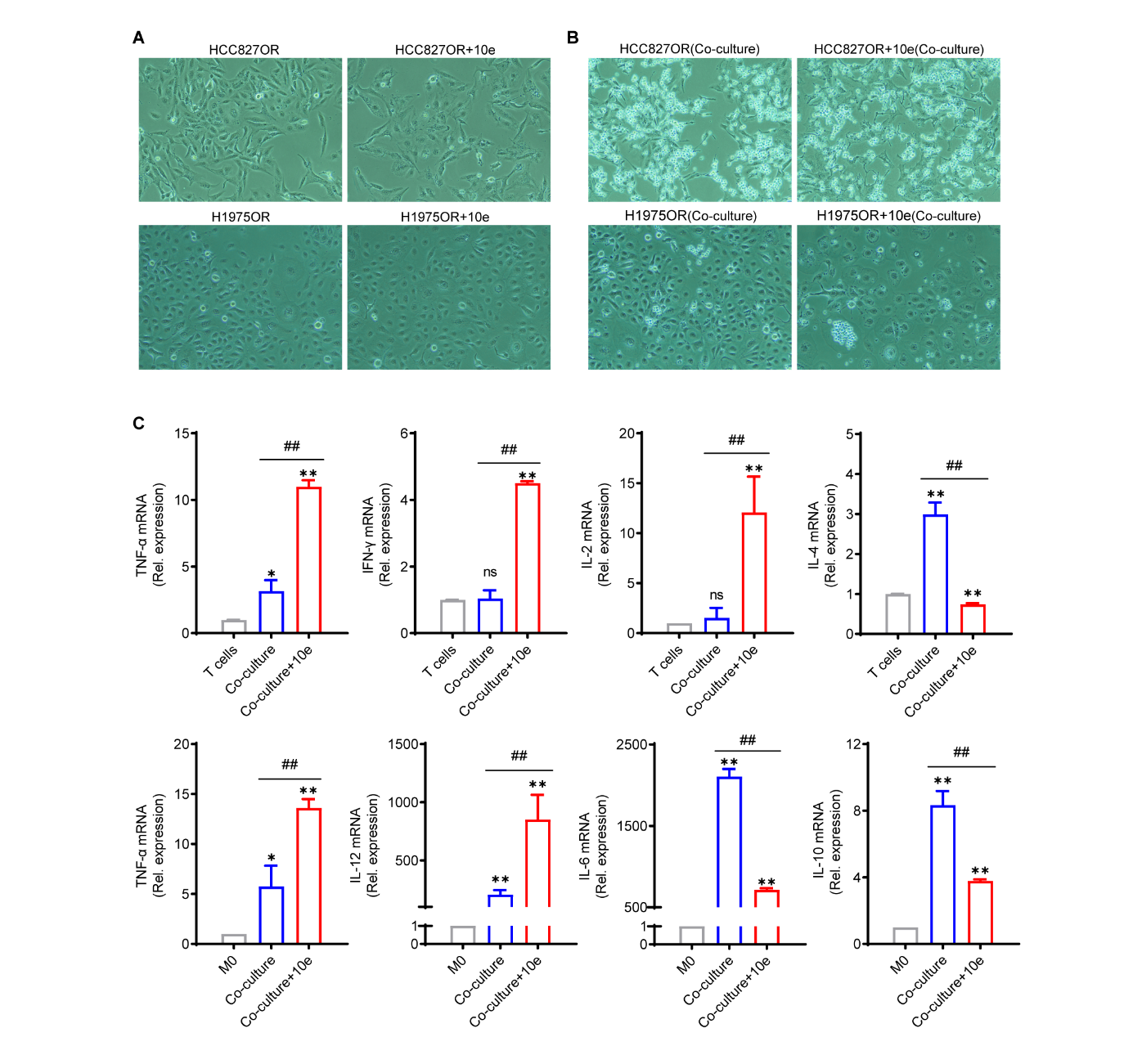


**Figure S18**: Alterations related to tumor cell phenotype and function following co-culture with immune cells.

A, B) Microscopic showing H1975OR (A) and HCC827OR (B) cell morphology following standard culture, treatment with 10e, co-culture system, and co-culture system combined with 10e treatment (scale bars, 100μm). C) Co-cultures of HCC827OR and immune cells were subjected to treatment with 10e (T: T cells, M: macrophages), changes in associated cytokines detected by qPCR (n= 3 per group). Data are presented as mean ± SD, calculated using two-sided unpaired Student’s t-test. * *P* < 0.05, ** *P* < 0.01, ^##^ *P* < 0.01, ns not significant, *P* > 0.05.


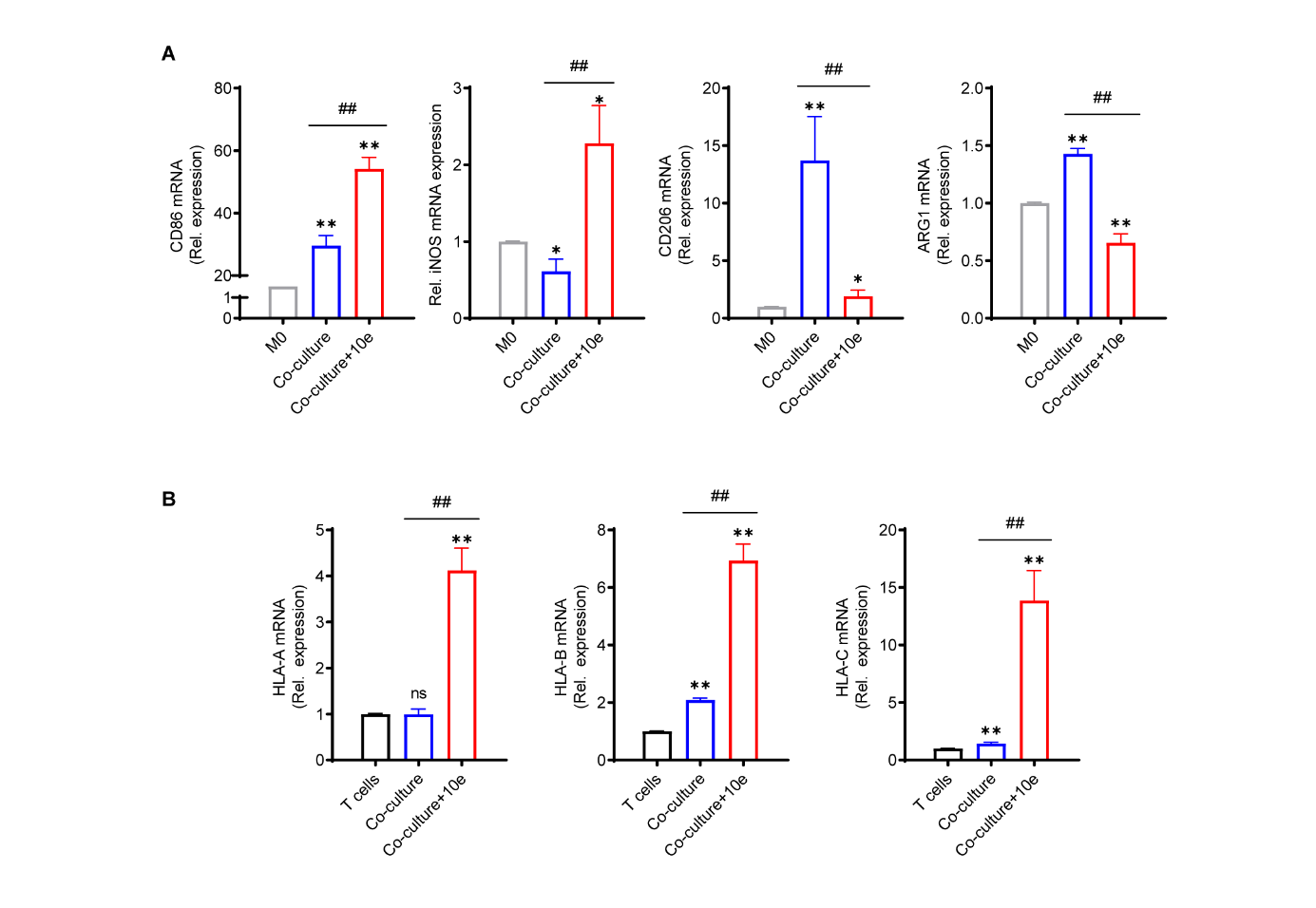


**Figure S19**: Alterations in immune cell-related markers identified through qPCR subsequent to co-culture with HCC827OR cells.

A, B) Co-cultures of HCC827OR and immune cells were subjected to treatment with 10e, changes in immune cell-related markers identified through qPCR (n= 3 per group). Data are presented as mean ± SD, calculated using two-sided unpaired Student’s t-test. * *P* < 0.05, ** *P* < 0.01, ^##^ *P* < 0.01, ns not significant, *P* > 0.05.


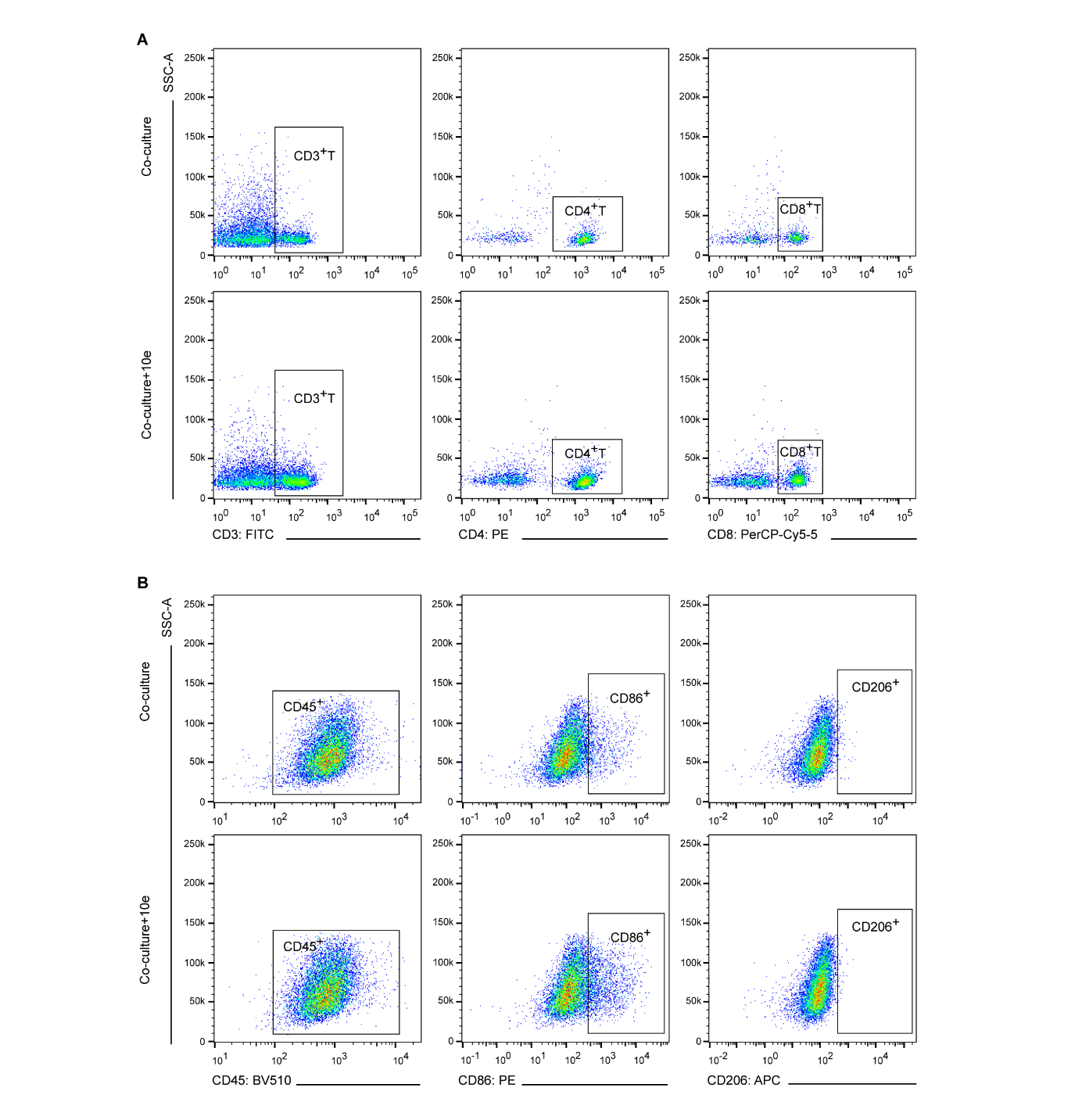


**Figure S20**: Flow cytometric analysis of immune cell typing following co-culture with HCC827OR cells.

A, B) Co-cultures of HCC827OR and immune cells were subjected to treatment with 10e. Tumor infiltrating lymphocyte (A) and macrophages (B) were characterized by flow cytometry analysis (n= 3 per group).


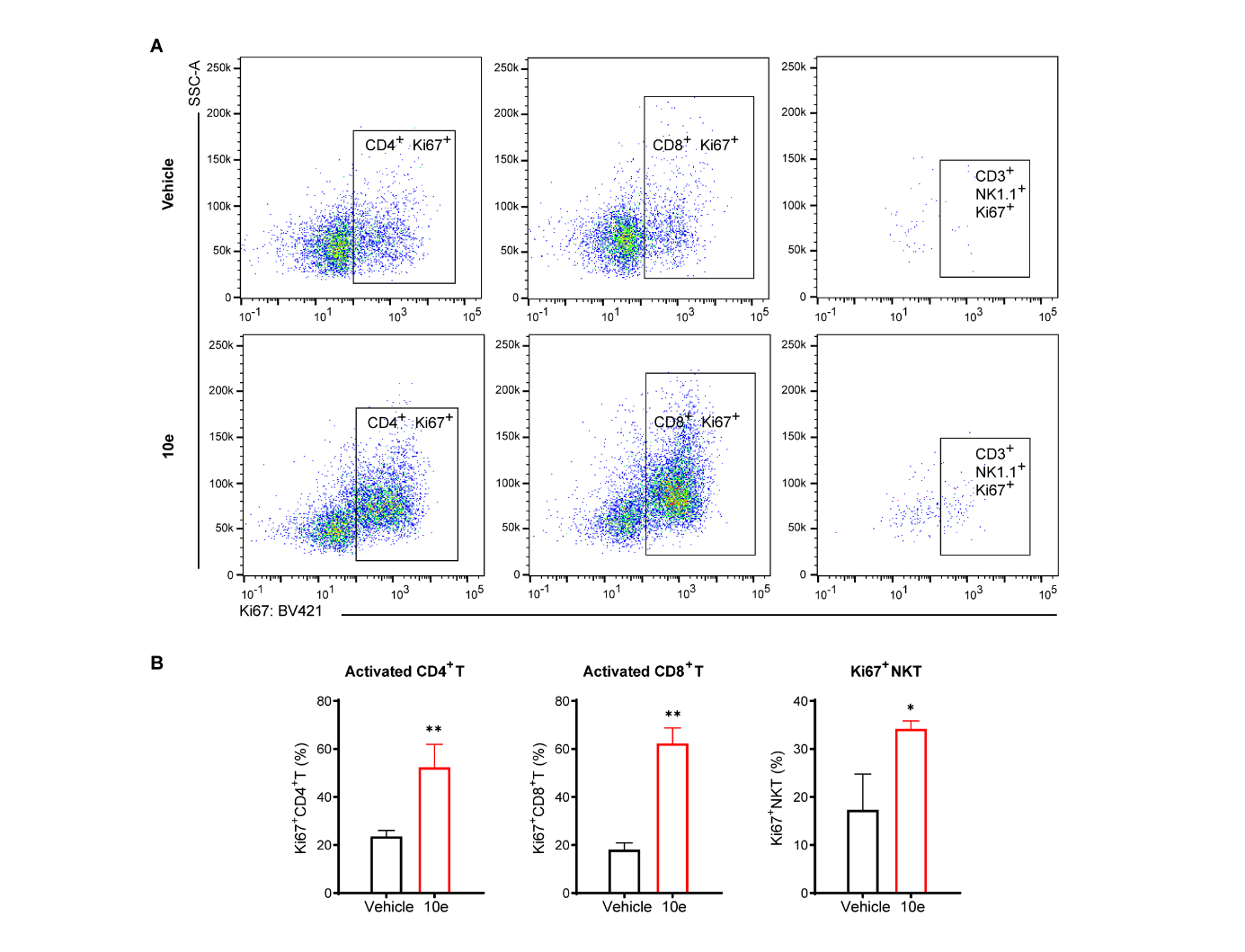


**Figure S21**: 10e treatment promotes an anti-tumor microenvironment in MC38-bearing mice.

A, B) MC38-bearing mice treated with 10e (0.5 mg/kg DON equivalent, subcutaneously, 6 days), representative flow cytometry plots (A) and data charts (B) showing activated CD4^+^T, activated CD8^+^T, and activated NKT cells subsets and ratios (n = 3 per group). Data are presented as mean ± SD, calculated using two-sided unpaired Student’s t-test. * *P* < 0.05, ** *P* < 0.01.


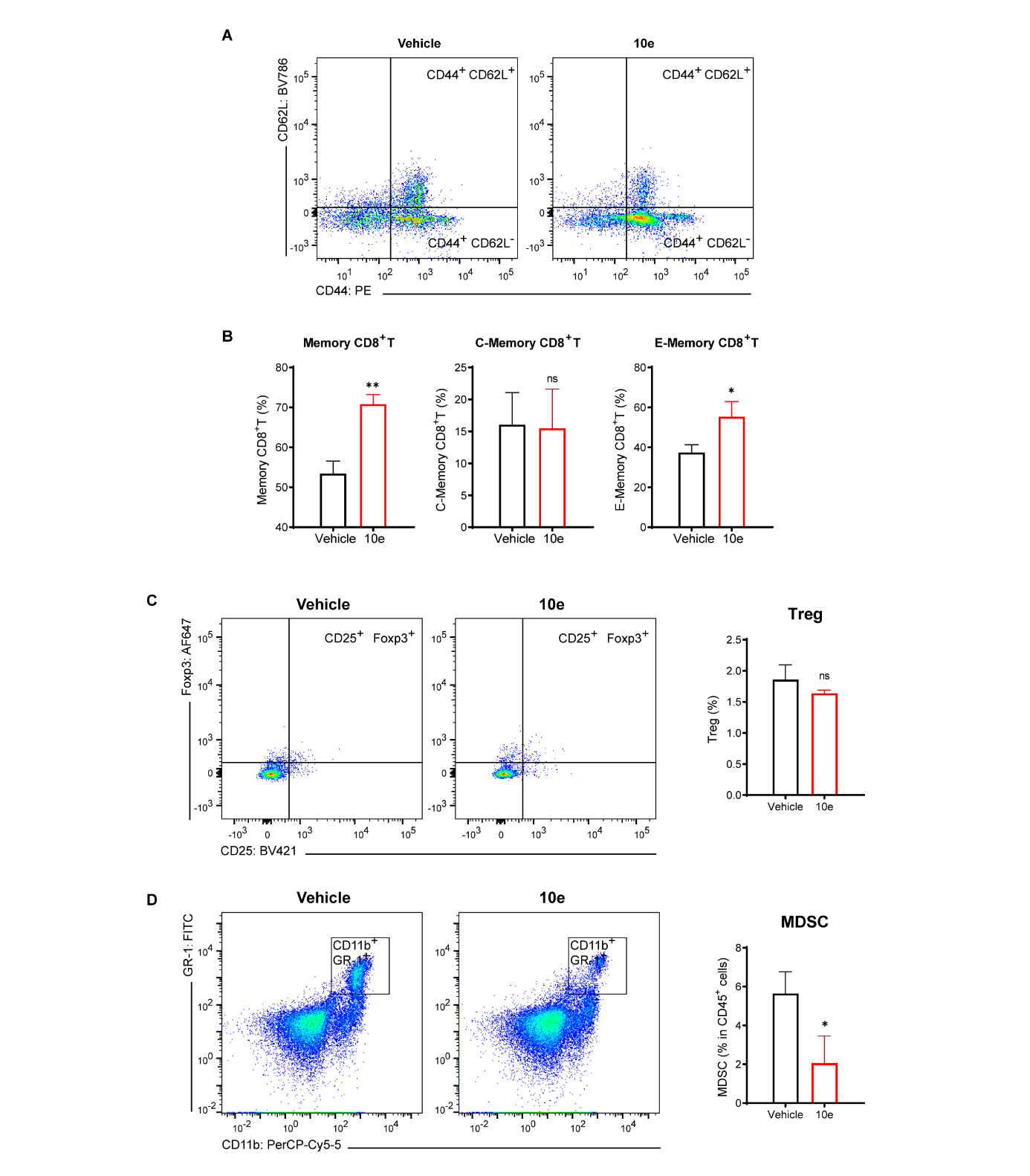


**Figure S22**: 10e treatment promotes an anti-tumor microenvironment in MC38-bearing mice.

A-D) MC38-bearing mice treated with 10e (0.5 mg/kg DON equivalent, subcutaneously, 6 days), representative flow cytometry plots (A) and data charts (B) showing memory CD8^+^T, central memory CD8^+^T, and effector memory CD8^+^T cells subsets and ratios, representative flow cytometry plots and data charts showing memory Treg (C) and MDSC (D) cells subsets and ratios (n = 3 per group). Data are presented as mean ± SD, calculated using two-sided unpaired Student’s t-test. * *P* < 0.05, ** *P* < 0.01, ns not significant, *P* > 0.05.


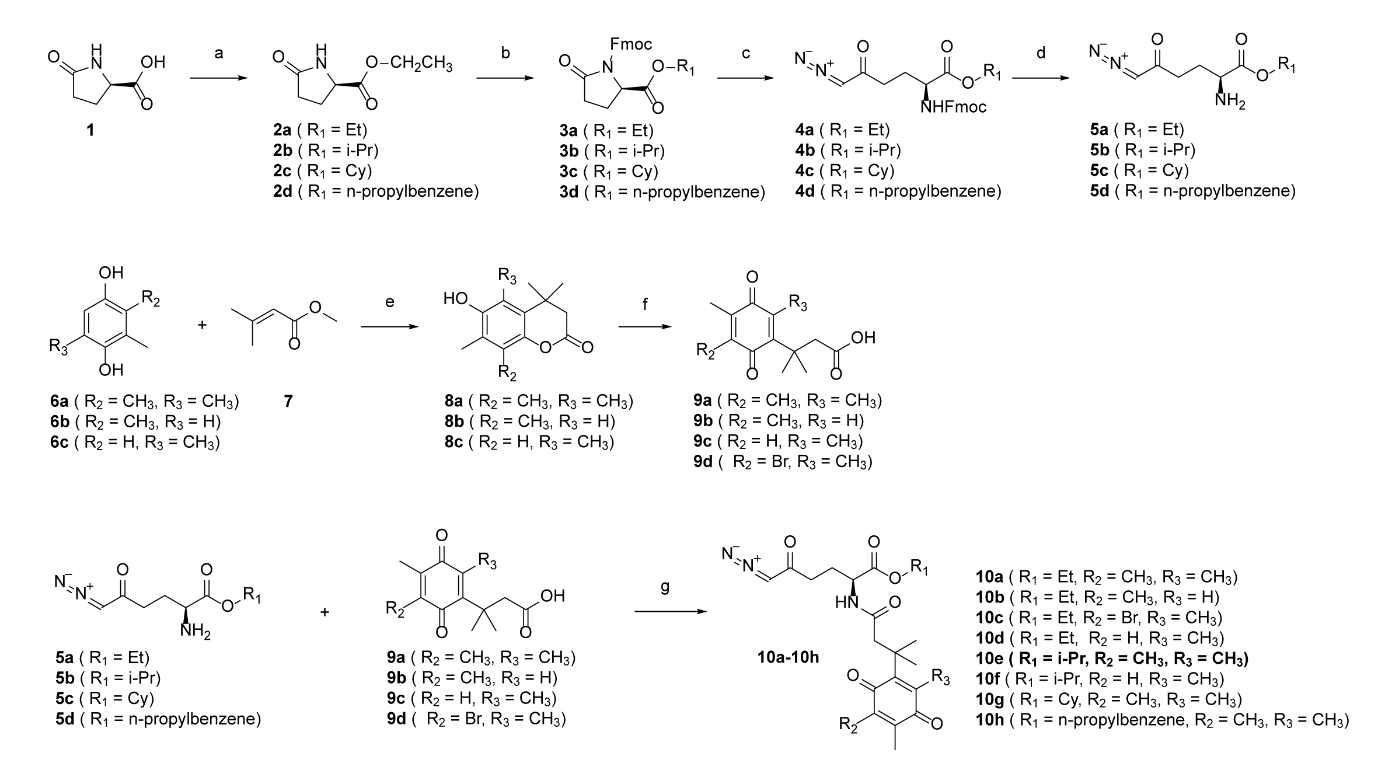


**Scheme S1** Synthesis of NQO1-responsive prodrugs of DON.

**Table S1**. IC_50_ value of V9302, CB839, LL202, CB839 plus V9302, LL202 plus V9302 on lung cancer cell lines.


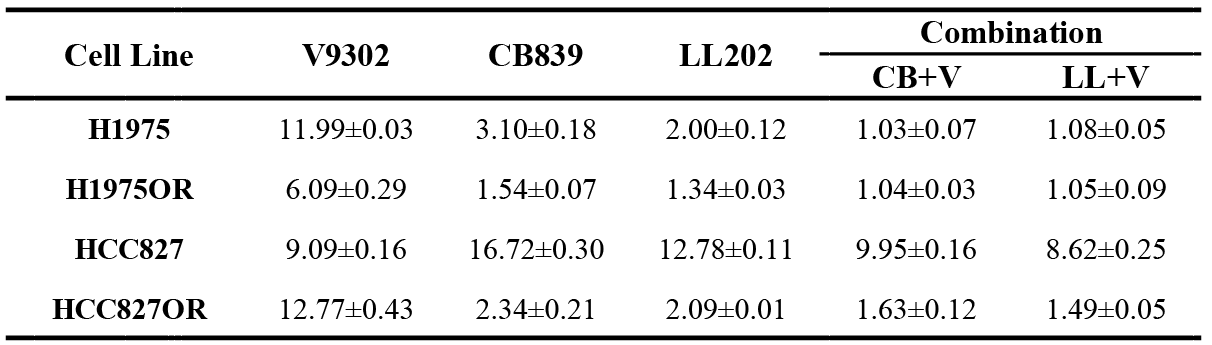


**Table S2**. CI value and Total dose of drug combo with different dose ratios.


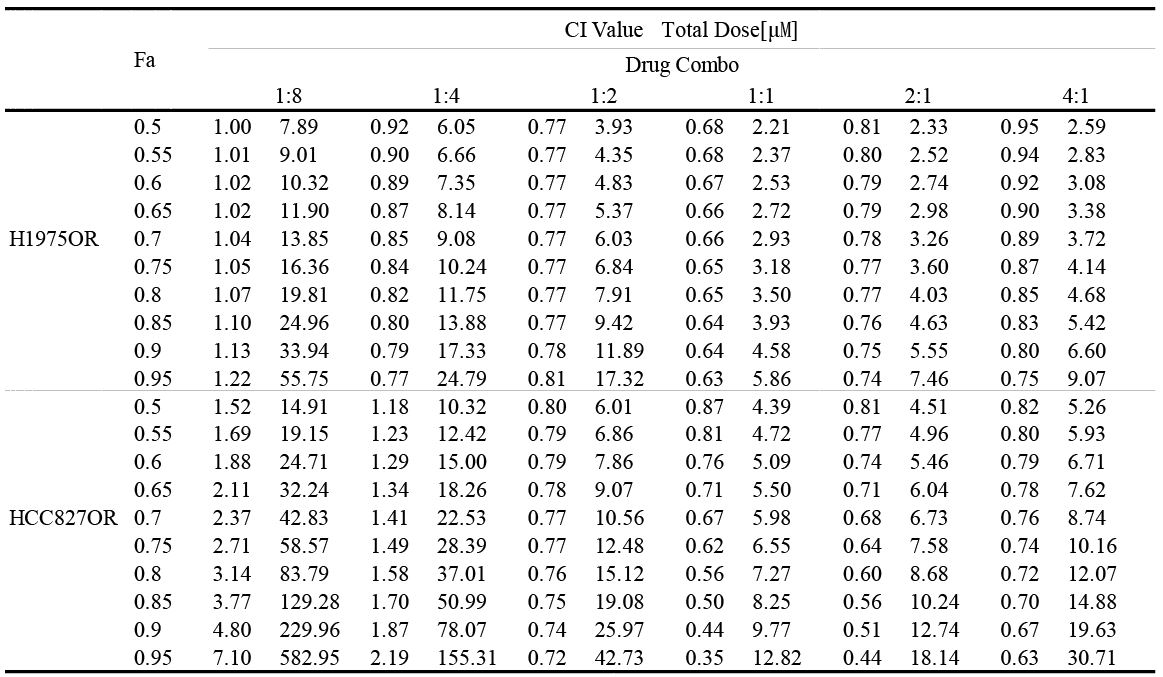


**Table S3**. CI value of GLS1 inhibition with ASCT2 inhibition on lung cancer cell lines.


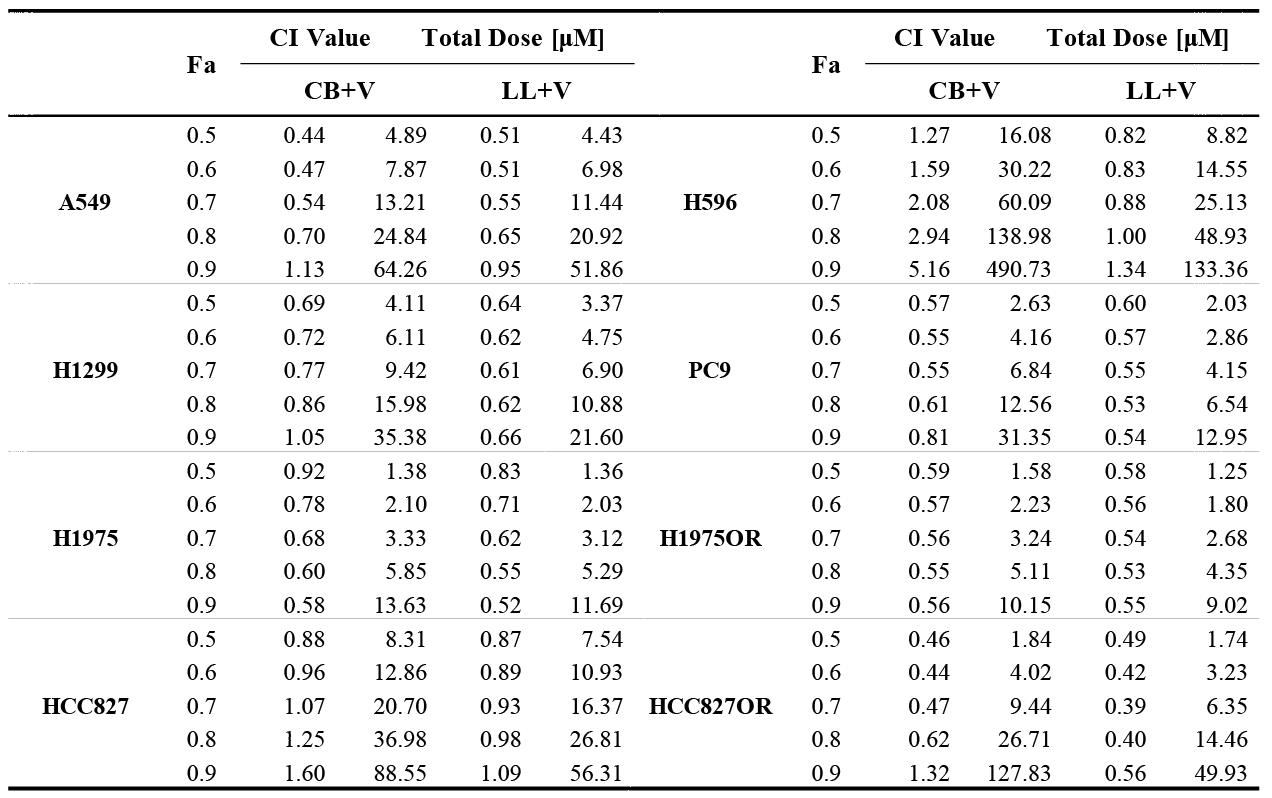


**Table S4**. Basic information on organoid samples.


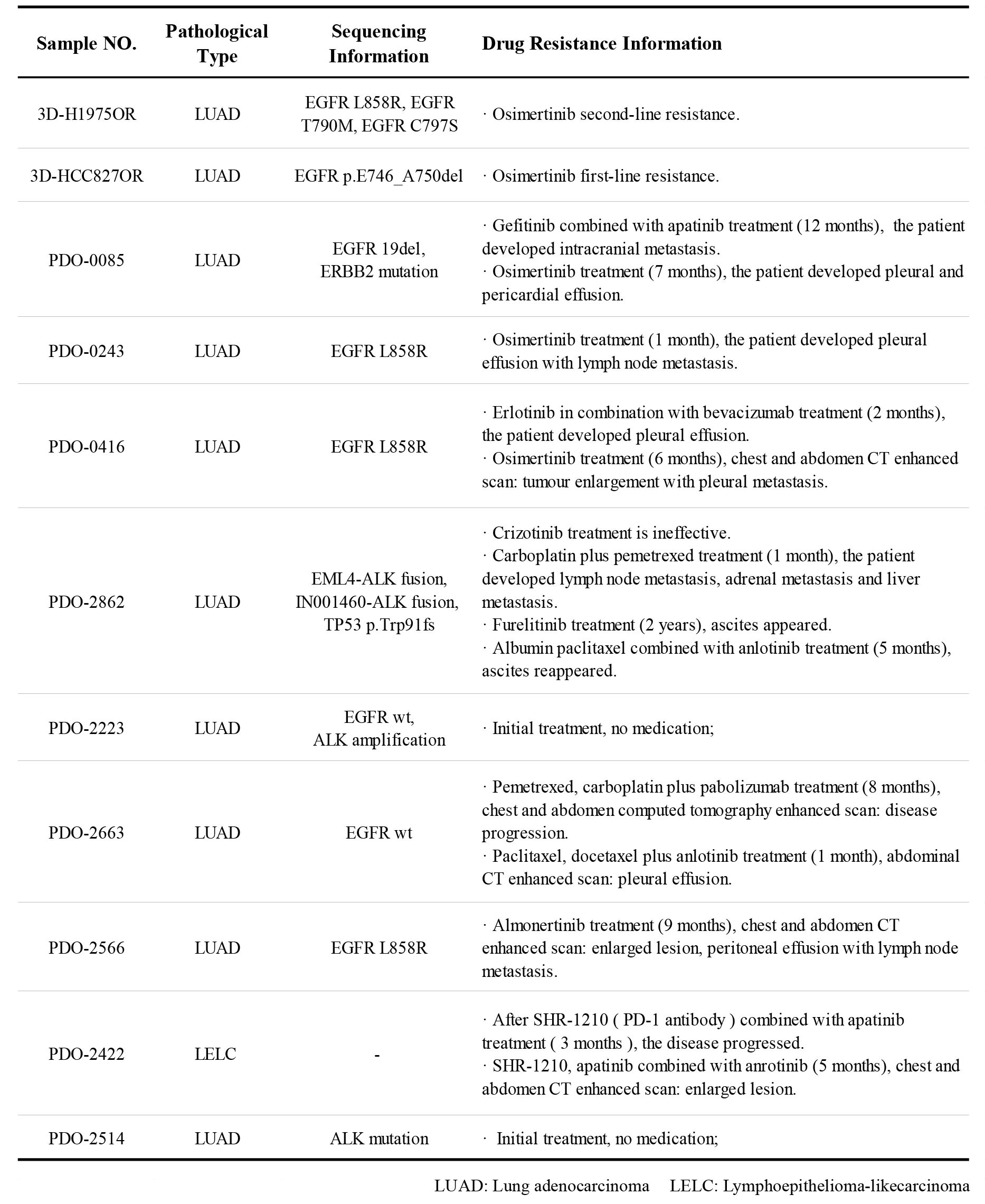


**Table S5**. Metabolic stability studies and metabolism by NQO1 of prodrugs 10a-10h in vitro.


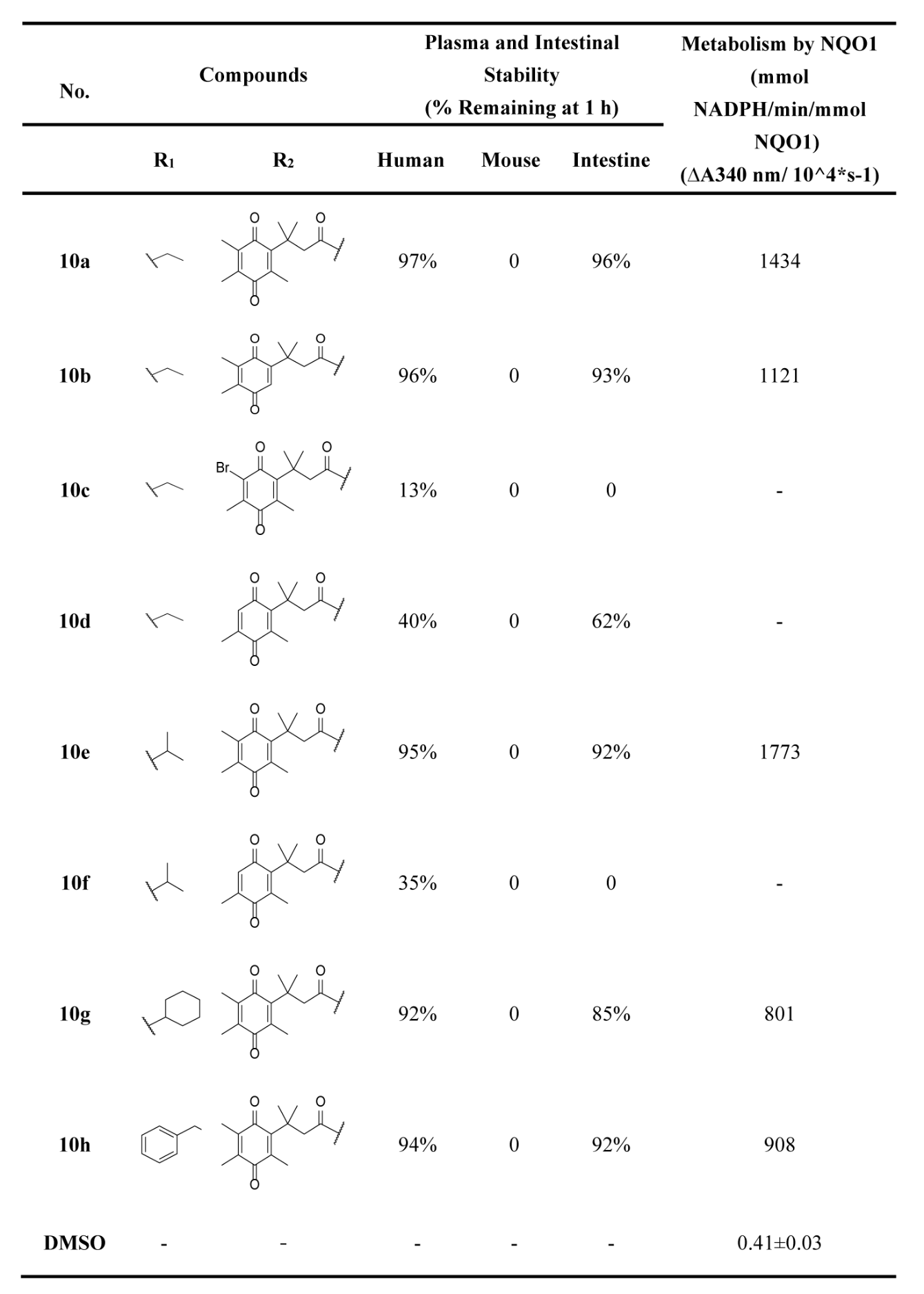


**Table S6**. Sequences of synthetic siRNAs.


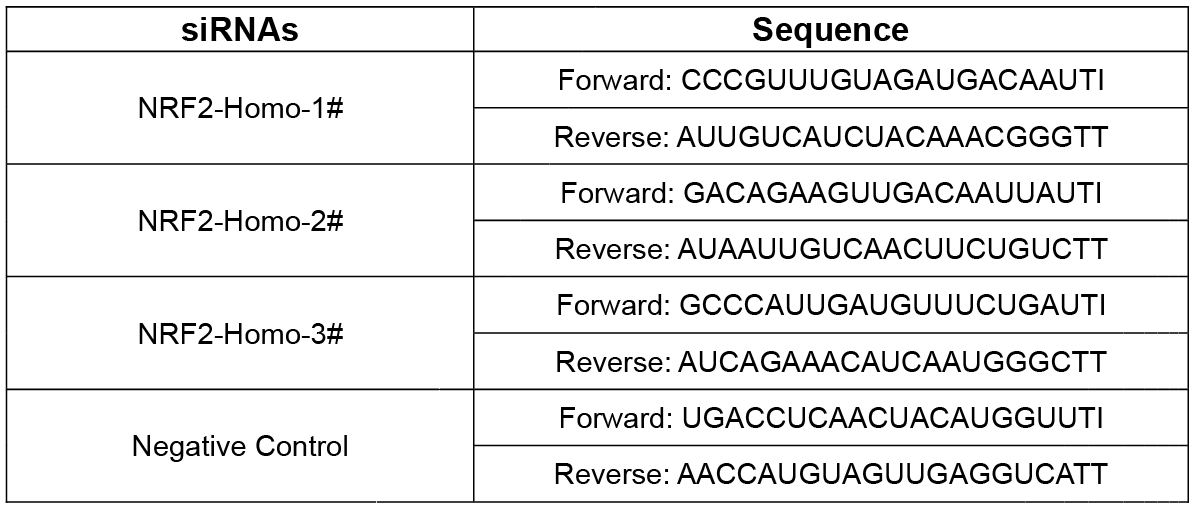


**Table S7**. Primary antibodies used for Western Blot in this study.


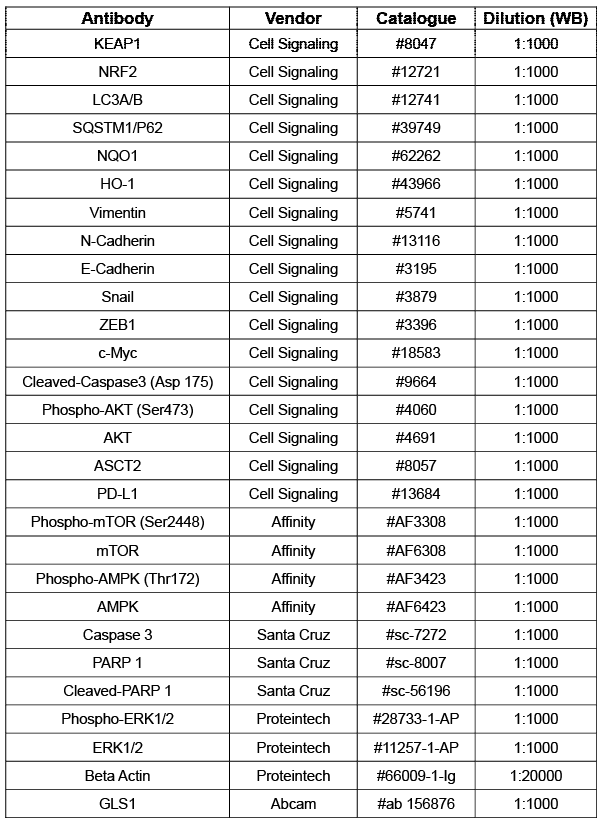


**Table S8**. Primer sequences of RT-qPCR used in this study.


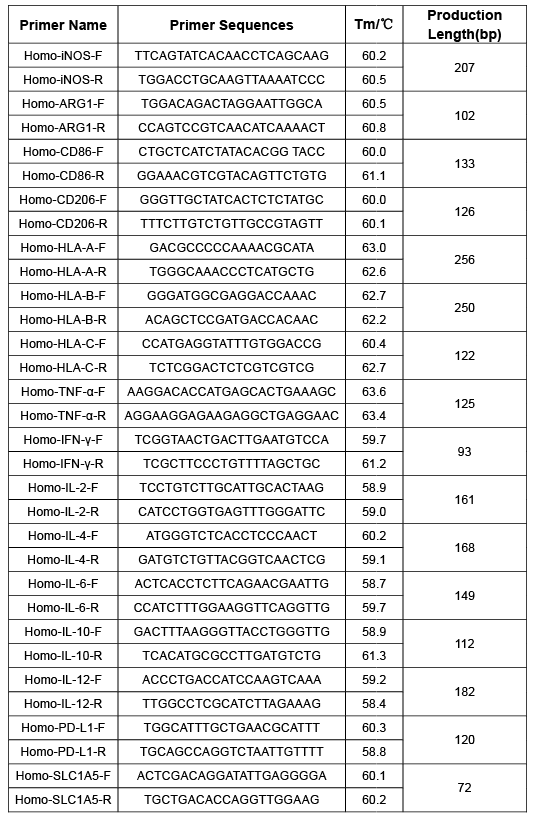


**Table S9**. Antibodies used for flow cytometry in this study.


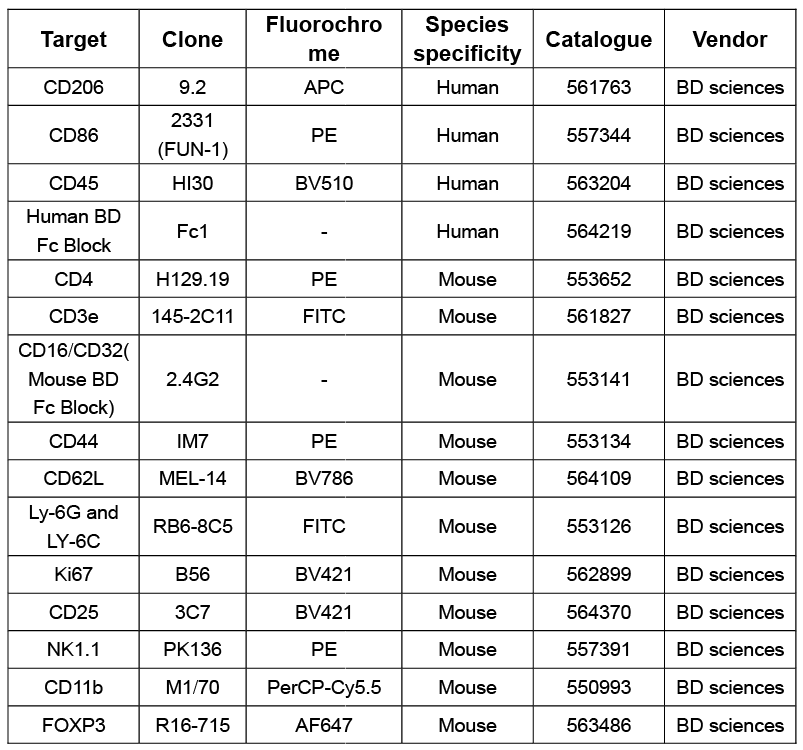

Supplement: Supplementary file 1 — Supporting Information [file ADVS-12-2411479-s001.docx]
